# Genome-Wide Association Studies Provide Insights into the Genetic Determination of Flower and Leaf Traits of *Actinidia eriantha*

**DOI:** 10.3389/fpls.2021.730890

**Published:** 2021-08-20

**Authors:** Guanglian Liao, Min Zhong, Zhiqiang Jiang, Junjie Tao, Dongfeng Jia, Xueyan Qu, Chunhui Huang, Qing Liu, Xiaobiao Xu

**Affiliations:** College of Agronomy, Jiangxi Agricultural University/Kiwifruit institute of Jiangxi Agricultural University, Nanchang, China

**Keywords:** *Actinidia eriantha*, flower, leaf, male vines, SNPs, GWAS

## Abstract

Kiwifruit (*Actinidia eriantha*) is a dioecious vine, and the pollen of its male cultivars has a direct effect on the quality of its fruits. In this study, to facilitate molecular breeding and gene identification, we performed genome-wide association studies (GWAS) on 11 traits of flower and leaf. A total of 946,337 highly consistent SNP markers were obtained in the whole genome. Phylogenetic tree analysis and population structure analysis showed that the 143 germplasms can be divided into two groups. The linkage disequilibrium analysis showed that *A. eriantha* have a relatively fast attenuation rate, and that the average attenuation distance of LD was 0.1–0.3 Kb. The MLM (QK) model was determined as best for correlation analysis, and eight and three SNPs associated with flower- and leaf-related traits were identified, respectively, at 0.01 significance level. However, SNP markers associated with stamen number per flower, pollen viability, total chlorophyll content, and total flavonoid content were not identified at the 0.01 significant level, although it is worth noting that one, one, five, and two SNPs were identified to be associated with these traits at the 0.05 significant level. This study provides insights into the complex flower- and leaf-related biology, and identifies genes controlling important traits in *A. eriantha* through GWAS, which extends the genetic resources and basis for facilitating molecular breeding in kiwifruits.

## Highlight

- Simplified genome sequencing technology was used for the first time to mine genes related to some important traits in 143 male *Actinidia eriantha*.

## Introduction

In China, *Actinidia eriantha* is a unique source of kiwifruit germplasm. It is mainly distributed in the Yangtze River basin, the Yunnan-Guizhou plateau, and the Sichuan basin at altitudes of 200–2,000 m (Liao et al., 2021). Fruits of *A. eriantha* are well known for their high nutrient content, especially ascorbic acid (AsA). In addition, because its flowers look good, *A. eriantha* is used as an ornament in parks and corridors. Furthermore, *A. eriantha* fruits can be easily peeled.

As we all know, kiwifruit is a dioecious vine. The pollen of its male cultivars has a direct effect on fruit quality. Therefore, in order to improve the yield and fruit quality of kiwifruit in the commercial production process, it is particularly important to breed superior pollination cultivars. There have been a lot of studies on the effect of pollen on fruit quality (Wu, 2015; Chen et al., 2019), but other research studies on male kiwifruit have been neglected, especially on the localization and identification of gene-related pollen, flower, and leaf. The team of the authors has been paying attention to this problem for a long time and has carried out a lot of research, such as investigation of wild kiwifruit germplasm resources (Liao et al., 2019a), relationship between ploidy and pollen traits (Liao et al., 2018b), variation in pollen morphology and flower characters (Zhong et al., 2016), and genetic diversity of wild male *A. eriantha* (Zhong et al., 2018, 2019). All these laid a foundation for this study to identify gene-related flower and leaf.

As for male kiwifruit, it is important to study variation of flower and leaf, which can reveal the genetic regular mechanism, and has important guiding significance for the exploration and utilization of existing resources, germplasm innovation and breed excellent parent materials, and can further improve the breeding efficiency (Mitchell-Olds et al., 2009). As genomes of more and more species are published, whole-genome association analysis (WGAS) has become an important way of mining some key regulating genes. For example, in tomato, 305 significant association sites related to fruit sugar, acid, amino acid, and flavor-related volatile content were determined through GWAS (Zhao et al., 2019). In pear (*Pyrus pyrifolia*), a total of 37 quantitative trait loci (QTLs) related to eight fruit quality traits and five QTLs related to three fruit phenological stage traits were detected through GWAS analysis (Zhang et al., 2021). However, there are few reports on GWAS analysis on *A. eriantha*, and there are no reports on male *A. eriantha*. This may be related to the late publication of the *A. eriantha* genome (Tang et al., 2019). It is worth mentioning that with the publication of the *A. eriantha* genome, this will be a hot spot for future research. In this study, 143 male germplasm resources were collected in Jiangxi province and selected as materials, 11 quantitative characters related to flower and leaf were determined. Then, the genome-wide SNP markers of the test materials were developed by the RAD-Seq technique, and the genetic diversity, population structure, and linkage imbalance in male germplasm of wild *A. eriantha* were analyzed. Finally, based on the SNP markers, GWAS analysis was carried out to mine the SNPs related to flower and leaf. this study can mine SNPs and key candidate genes that are significantly associated with target traits, and provide theoretical basis for the breeding of new cultivars and germplasm innovation of *A. eriantha*.

## Materials and Methods

### Materials

The test materials were collected from Mugu mountain (MGS), Jinggang mountain (JGS), Wugong mountain (WGS), and Wufu mountain (WFS) in Jiangxi Province, China (Table 1). Based on the previous information of locations, we collected the corresponding flower and leaf at the full flowering stage in 2019. All the samples were diploid (Liao et al., 2018a).

**Table 1:** Sample information on male *Actinidia eriantha*.

**Sample name**	**Number**	**Sampling region**	**Geographical location**	**Altitude (m)**
MGS	105	Magushan, Fuzhou city, Jiangxi Province	E 116 31′58.90″-116°33′12.26″	668–961
			N 27°31′08.35″-27°32′02.96″	
JGS	13	Jinggangshan, Ji'an city, Jiangxi Province	E 114°09′01.34″-114°11′26.32″	798–913
			N 26°32′47.63″-26°36′55.13″	
WGS	14	Wugongshan, Pingxiang city, Jiangxi Province	E 114°06′52.11″-114°07'20.87″	308–368
			N 27°32′29.42″-27°33'05.53″	
WFS	11	Wufushan, Shangrao city, Jiangxi Province	E 118°01′16.59″-118°04′35.08″	650–770
			N 28°00′27.53″-28°01'57.20″	

### Determined Characters of Flower and Leaves

Fifteen male flowers at the popcorn stage just prior to opening were randomly selected from each male sample, and the stamens were peeled off to calculate its number. The number of petals and stamens in a single flower was counted. Corolla diameter was measured with a vernier caliper. Pollen viability was measured by an *in vitro* germination methodology (Seyrek et al., 2016), and pollen quantity was measured with a blood cell counting plate (Wang et al., 2017). Then, the petals were frozen in liquid nitrogen and stored in a refrigerator at −80°C for subsequent determination of anthocyanin content, which was measured using a differential pH method (Liu et al., 2009).

Five mature leaves in the middle of 1-year-old canes were collected, and the veins were removed. Then, they were also frozen in liquid nitrogen and stored in a refrigerator at −80°C for subsequent determination of relative quantitative characters. The AsA content was determined by 2, 6-dichloroindigophenol titration (Gao, 2006). The Folin–Ciocalteu method was used to determine the total phenol content (Pastrana-Bonilla et al., 2003). The total flavonoid and total chlorophyll contents were determined by aluminum nitrate colorimetry (Jia et al., 1999), and with a spectrophotometer (Li, 2007), respectively. A completely randomized design with three replicates was used.

### Construction of RAD Library and High-Throughput Sequencing

Genomic DNA was extracted using the Magen Hipure Plant DNA Mini Kit (Guangzhou, China), and the quality was tested according to previous methods (Liao et al., 2019b). The genome of *A. eriantha* was selected as reference (Tang et al., 2019), and the RAD library was constructed using an EcoRI restriction enzyme (GAATTC) (Gao et al., 2017). The constructed RAD library was sequenced with Illumina HiSeq^TM^ 2000 (Illumina, San Diego, CA, United States). Library construction and computer sequencing were completed by Gene Denovo Co. (Guangzhou, China). After the removal of low-quality reads, clean reads were mapped to the reference genome using the BWA v0.7.12 software (Li and Durbin, 2009). Then, population SNP detection was performed using Genome Analysis Toolkit (GATK) (Mckenna et al., 2010). A Bayesian model was used to detect polymorphic loci in the population, and SNPs with deletion rate <0.5, heterozygosity rate <0.8, and minimum allele frequency >0.05 were filtered out to obtain high-quality population SNP markers (Alexander et al., 2010). Finally, high quality SNPs were analyzed for linkage disequilibrium and genetic diversity (Francis, 2017). The data were uploaded to NCBI, and the ID is PRJNA742212.

### GWAS Analysis

The genome-wide association study was analyzed for 11 quantitative traits with the GEMMA V0.98.1 software (Zhou and Stephens, 2012). In order to compare the effects of different correlation models on the correlation results, four different correlation models were used in this study, they are general linear model (GLM), GLM (Q), MLM (K), and MLM (QK) models. The Admixture V1.3 software was used to calculate the population structure matrix Q, and the GCTA V1.93.2 software was used to calculate the relationship matrix K among the samples. Finally, an association value (*P*) was obtained for each SNP locus, and the significant *P* value was corrected according to the previous methods (Zheng et al., 2012; Fadista et al., 2016). The two *P* values were displayed by Manhattan plot and Quantile-by-quantile plot after taking –log10. The genes near significant SNP locus 50 Kb were selected as candidate genes and annotated by the KGD database (http://kiwifruitgenome.org/).

### DNA Extraction and PCR Verification

Leaves were used for DNA extraction, and quality assessment was performed following a previous study (Liao et al., 2019b). The content of DNA was diluted to 150 ng/μl and stored at −80°C. We selected two SNP sites related to leaf AsA content for verification, and designed forward and reverse primers at the first and the last 150 bp of the SNP site (Supplementary Material 1). The PCR reaction system also referred to the previous method (Liao et al., 2019b). Sequencing was performed by TSINGKE Biotechnology Co., Ltd.

### Data Analysis

Microsoft Excel 2016 was used to conduct flower- and leaf-related trait data statistics. Significant analysis and regression analysis were performed using IBM SPSS Statistics 22.0.

## Results

### Statistical Results of Flower- and Leaf-Related Traits

**Figure 1:**
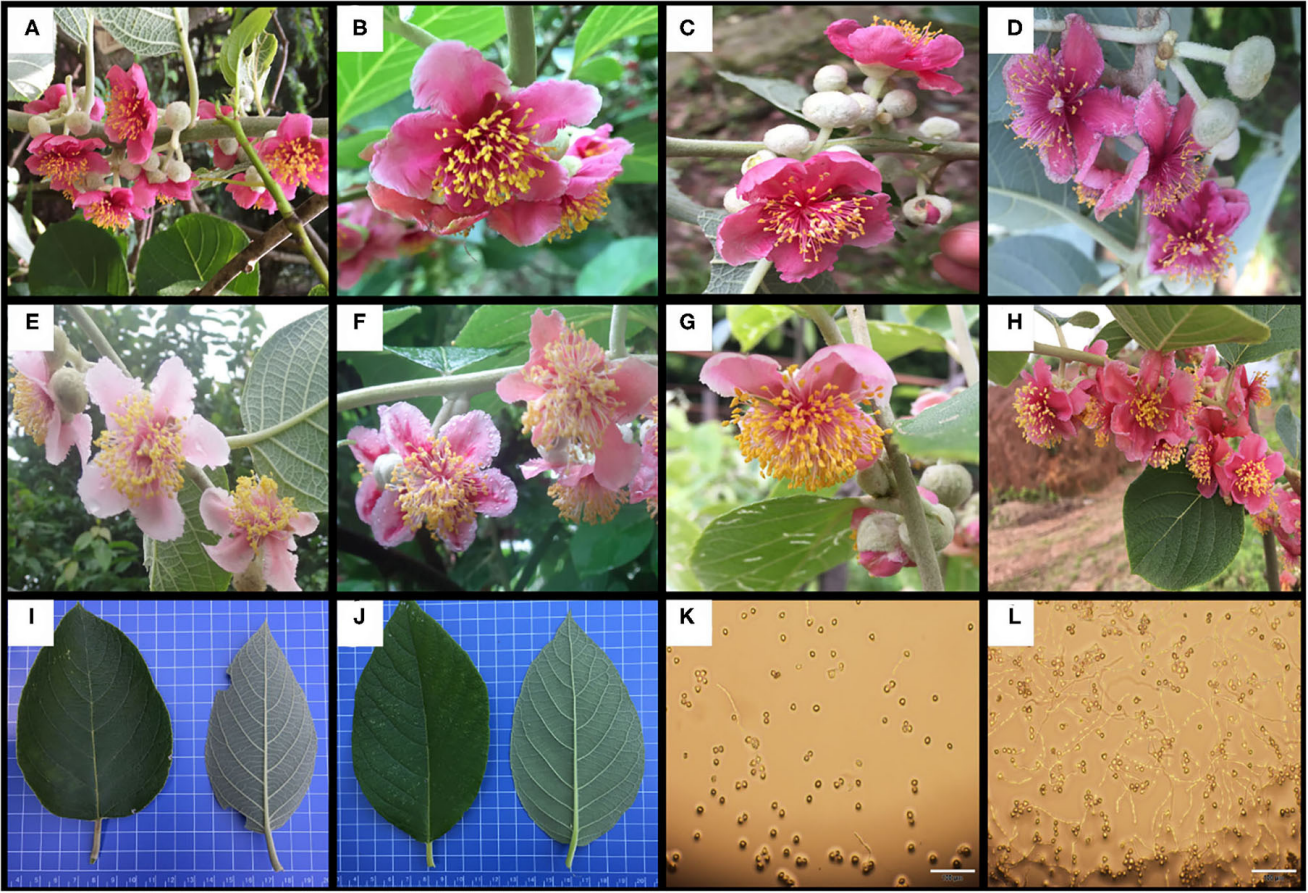
Some germplasm with specific traits. **(A–D)** the petals have higher anthocyanin content; **(E–H)** the flower contains more stamens; **(I, J)** different leaf shapes, the indicators on the leaf are mainly some quality indicators; **(K)** low pollen viability type; **(L)** high pollen viability type.

The number of petals per flower (NPF), number of stamens per flower (NSF), corolla diameter (CD), amount of pollen per flower (APF), amount of pollen per anther (APA), pollen viability (PV), anthocyanin content in petals (ACP) in flower and AsA content (AL), total chlorophyll content (TCL), total phenols content (TPL), and total flavonoids content (TFL) in leaf of test materials were determined statistically (Supplementary Material 2). In the flower, the ranges in variation of NPF, NSF, APF, PV, and ACP were 4.67–6.5 petals/flower, 75.67–294.25 stamens/flower, 3.18 × 10^5^-3.20 × 10^6^ pollen/flower, 20.7–95.56%, and 12.08–95.09 mg/100g, respectively. In the leaf, the AL, TCL, TPL and TFL were 43.33–365.9, 31.09–142.12, 1.21 × 10^4^-4.84 × 10^4^, and 50.13–80.49 mg/100 g, respectively (Table 2 and Figure 1).

**Table 2:** Statistical analysis of the 11 flower and leaf related traits.

**Sample name**	**Min**	**Max**	**Mean**
NPF	4.67	6.5	5.44
NSF	75.67	294.25	154.53
CD (mm)	22.71	45.43	33.27
PV (%)	20.7	95.56	67.4
APA	2.13 ×10^3^	1.74 ×10^4^	7.73 ×10^3^
APF	3.18 ×10^5^	3.20 ×10^6^	1.20 ×10^6^
ACP (mg/100 g)	12.08	95.09	36.34
AL (mg/100 g)	43.33	365.9	149.26
TPL (mg/100 g)	12106.6	48779.46	31644.21
TFL (mg/100 g)	50.13	380.49	153.07
TCL (mg/100 g)	31.09	142.12	90.19

*NPF, number of petal per flower; NSF, number of stamen per flower; CD, corolla diameter; PV, pollen viability; APA, amount of pollen per anther; APF, amount of pollen per flower; ACP, anthocyanin content of petal; AL, ascorbic acid (AsA) content of leaf; TPL, total phenol content of leaf; TFL, total flavonoid content of leaf; TCL, total chlorophyll content of leaf*.

### Sequencing Data Statistics and Quality Assessment

A total of 164.84 Gb data were obtained by sequencing, with an average of 1.15 Gb per sample. Q20 and Q30 of sequencing data were 96.94 and 90.12%, respectively. The mapping rate to the reference genome was between 76.9 and 97.25%, and the sequencing depth was between 4.90 and 11.11 X (Table 3).

**Table 3:** Sequencing data statistics and quality assessment.

	**Raw data (bp)**	**Clean data (bp)**	**Q20 (%)**	**Q30 (%)**	**Mapping ratio (%)**	**Average depth (X)**
Max	2,006,749,560	1,964,916,453	98.54	94.97	97.25	11.11
Min	701,083,240	686,192,708	96.94	90.12	76.90	4.90
Means	1,152,748,675	1,126,907,787	97.59	92.55	95.70	7.38

### SNP Statistics

A total of 6,022,072 SNP markers were detected by the GATK software, and 946,337 high-quality SNP markers were obtained after filtration, distributed on different chromosomes, among which most SNP markers were 45,123 on Chr03 (Table 4).

**Table 4:** Distribution of single nucleotide polymorphisms (SNPs) on each chromosome.

**Chromosome**	**Before filter SNPs**	**After filter SNPs**	**Chromosome**	**Before filter SNPs**	**After filter SNPs**
Chr 01	218,168	35,125	Chr 16	227,118	35,097
Chr 02	174,177	27,259	Chr 17	200,319	30,192
Chr 03	273,926	45,123	Chr 18	237,113	36,942
Chr 04	148,268	22,563	Chr 19	230,817	37,660
Chr 05	196,176	30,355	Chr 20	203,448	32,171
Chr 06	199,907	31,468	Chr 21	187,357	28,484
Chr 07	227,064	35,424	Chr 22	196,149	31,300
Chr 08	281,171	43,892	Chr 23	229,604	38,883
Chr 09	179,448	27,421	Chr 24	202,240	31,274
Chr 10	206,191	30,623	Chr 25	188,817	30,129
Chr 11	175,706	25,632	Chr 26	198,839	33,195
Chr 12	214,479	32,969	Chr 27	199,960	31,608
Chr 13	211,390	34,560	Chr 28	195,438	30,724
Chr 14	197,789	31,056	Chr 29	193,872	29,301
Chr 15	191,824	30,700	Chr 00	35,297	5,207

### Evolutionary Analysis

**Figure 2:**
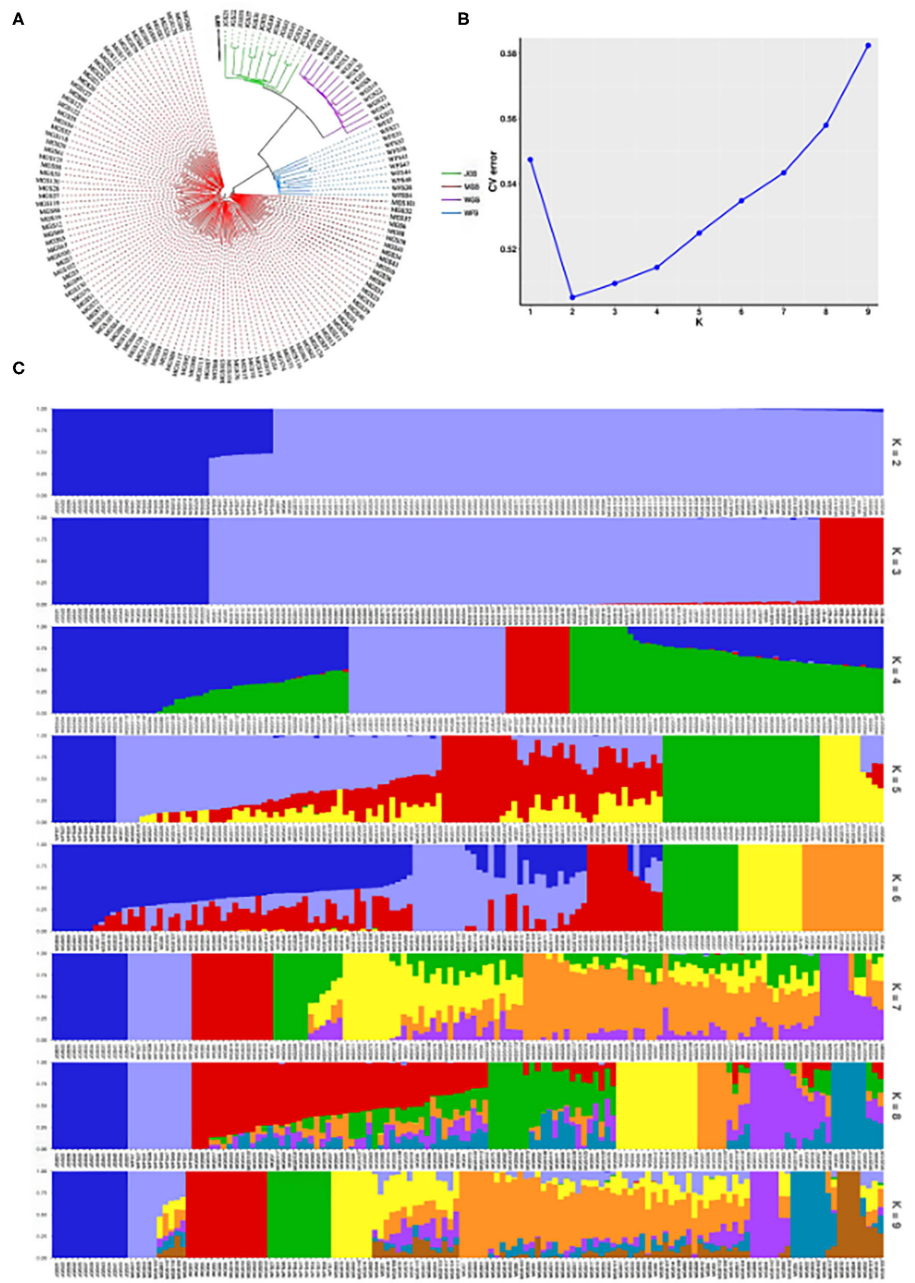
Evolutionary analysis. **(A)** Phylogenetic tree of the 143 male simples; red, blue, purple and green represent the MGS, WFS, WGS, and JGS populations, respectively; **(B)** cross-validation error analysis; **(C)** genetic structure map, the horizontal axis shows the different germplasm numbers, the vertical axis shows the probability of germplasm being grouped at a certain K value, different colors represent different groups.

A phylogenetic tree was constructed based on high-quality SNP markers obtained after filtration. The phylogenetic tree showed that there were some genetic differences among the samples, so the test materials were divided into two groups: 105 germplasm from the MGS group and 11 germplasm from the WFS group, 14 germplasm from the WGS group and 13 germplasm from the JGS group (Figure 2A). The inference results of the group structure obtained using the Admixture V1.3 software were consistent with the results of cluster analysis. According to the principle of minimum error rate of cross validation, the optimal number of groups was determined to be 2 (Figures 2A,B). When K = 2, the 143 male vines in the genetic structure map were divided into two groups. With the increase in K value, the increase in the number of ancestor groups indicated that more subgroups could be separated from each other. When K = 4, the WGS and JGS groups can also be separated (Figure 2C).

### Attenuation Analysis of Linkage Disequilibrium (LD)

**Figure 3:**
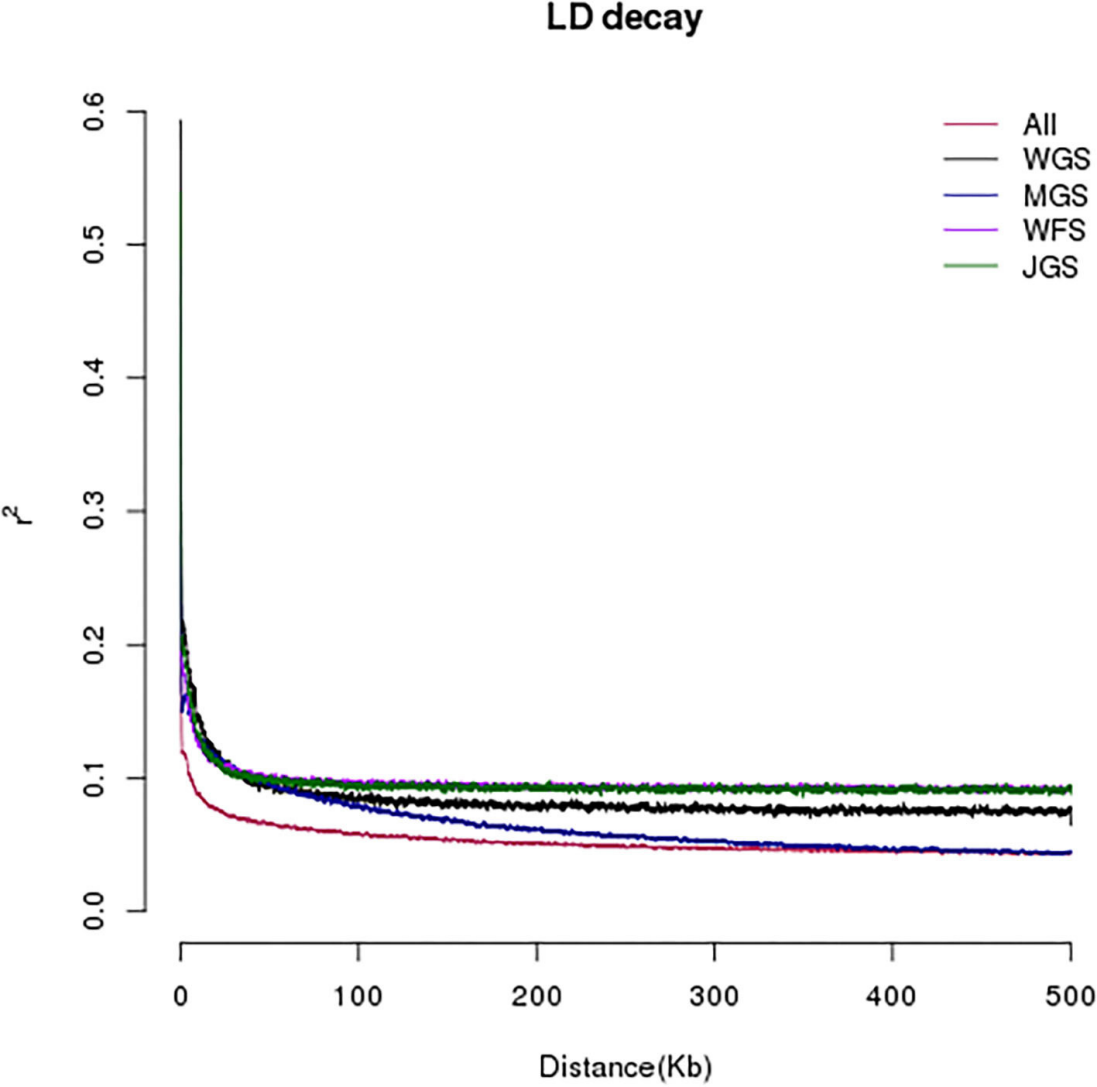
Model of linkage disequilibrium (LD) decay of the 143 male *Actinidia eriantha*.

In order to develop highly consistent single nucleotide polymorphism markers, the linkage situation of single nucleotide polymorphisms in the all samples was analyzed, which was represented by R^2^. Generally, the attenuation distance of linkage imbalance was taken as the distance traveled when R^2^ dropped to half of the maximum value. As can be seen from Figure 3, the LD attenuation distance of the four group populations of *A. eriantha* from different geographical sources is between 0.1 and 0.3 Kb.

### Comparison of Different Correlation Models

**Figure 4:**
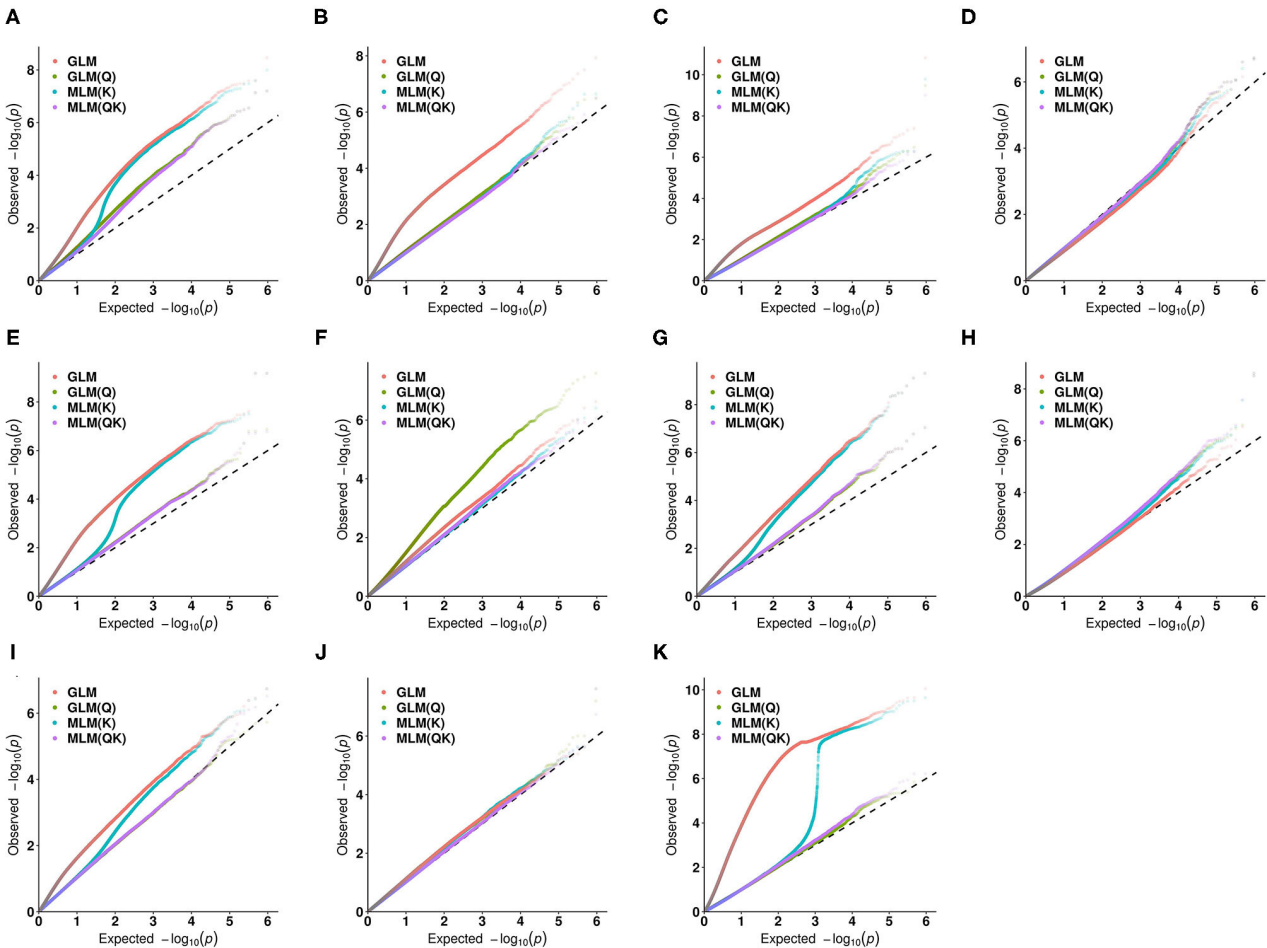
Quantile-quantile (QQ) plots for the 11 quantitative traits under different association models. **(A)** the amount of pollen per flower; **(B)** the number of stamens per flower; **(C)** the corolla diameter; **(D)** the amount of pollen per flower; **(E)** the amount of pollen per anther; **(F)** pollen viability (PV); **(G)** the anthocyanin content of petal (ACP); **(H)** the ascorbic acid content of leaf (AL); **(I)** the total chlorophyll content of leaf (TCL); **(J)** the total phenol content of leaf (TPL); **(K)** the total flavonoid content of leaf (TFL).

In this study, in order to select the optimal association model, the number of significant single nucleotide polymorphisms (SNPs) for the 11 quantitative traits under different association models was compared. Data analysis showed that the GLM model was associated with the largest number of SNPs (Supplementary Material 3), followed by the MLM (K) model, indicating that the false positive rate was higher under these two models. Since the MLM (QK) model takes into account the dual effects of population structure and relatedness, it was adopted as the optimal model in the subsequent association analysis (Figure 4).

### Identification of Key Gene-Related Flowers and Leaves

**Figure 5:**
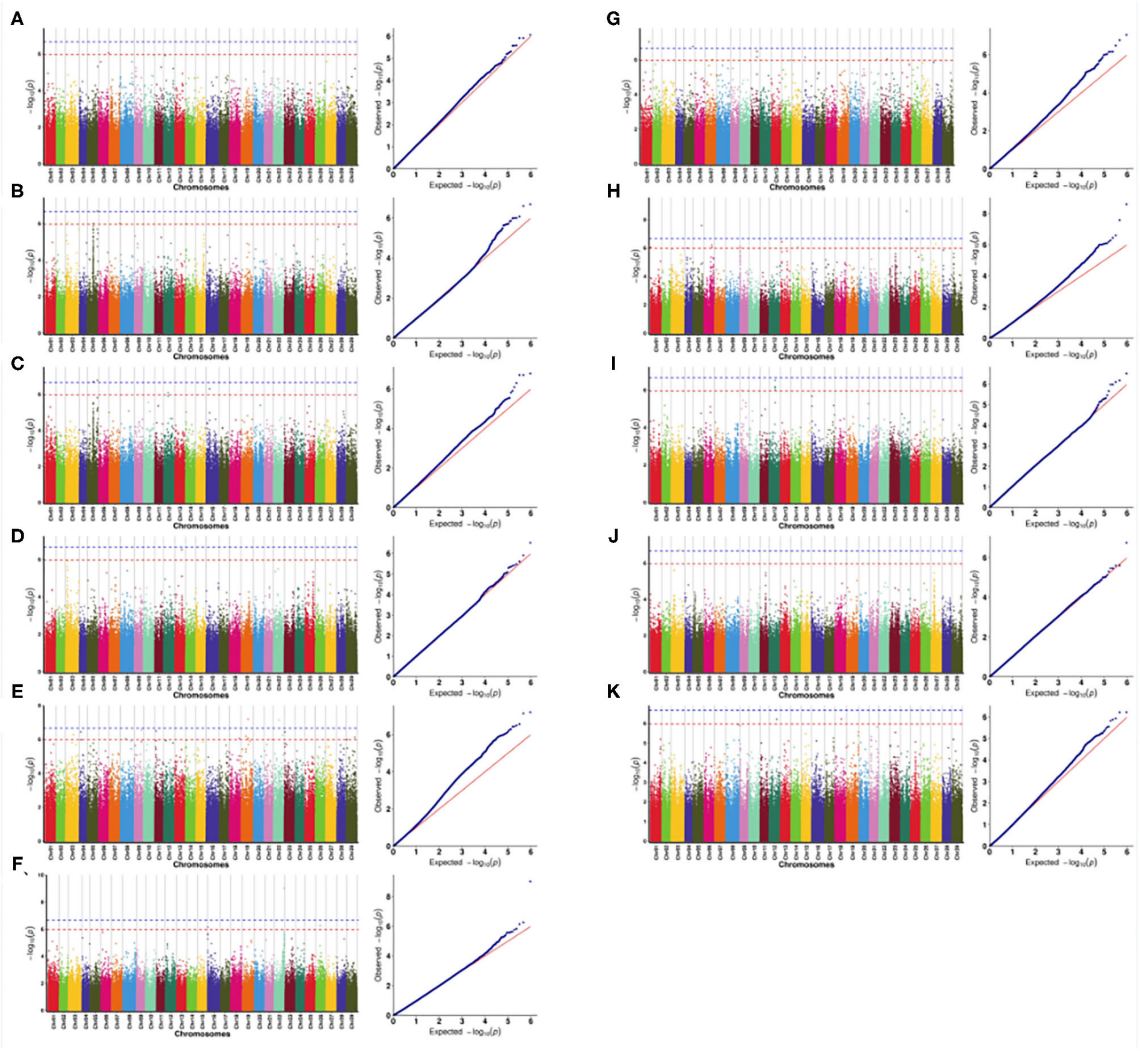
Manhattan and QQ plots of the genome-wide association study (GWAS). **(A)** pollen viability; **(B)** the amount of pollen per flower; **(C)** the amount of pollen per anther; **(D)** the number of stamens per flower; **(E)** the amount of pollen per flower; **(F)** corolla diameter; **(G)** the anthocyanin content of petal; **(H)** the ascorbic acid content of leaf; **(I)** the total chlorophyll content of leaf; **(J)** the total phenol content of leaf; **(K)** the total flavonoid content of leaf. The blue dotted line represents a level of 0.01, and the red dotted line represents a level of 0.05.

The correlation analysis results of pollination traits (pollen viability, amount of pollen per flower, amount of pollen per anther, and number of stamen per flower) of male *A. eriantha* are shown in Figures 5A–D. No SNPs significantly associated with PV and NSF were detected at the 0.01 significant level [–log10(*P*) > 6.67], but there was an SNP significantly associated with PV and NSF at the 0.05 significant level [–log10(*P*) > 5.97], located on chromosomes 6 and 13. At the 0.01 significant level [–log10(*P*) > 6.67], two SNPs significantly associated with APA were detected, all of which were located on chromosome 5. Among the two SNPs associated with APA, one of them was also associated with APF at the 0.01 level. Subsequently, a total of 24 candidate functional genes that may be related to pollination traits were found at five SNPs, and 14, four and six candidate genes related to APA, PV, and NSF, respectively, were found (Supplementary Material 4). As for petal traits, at the 0.01 significant level [–log10(*P*) > 6.67], a total of two SNPs significantly associated with NPF were detected on chromosomes 19 and 22, respectively (Figure 5E). One SNP significantly associated with CD was detected on chromosome 22 (Figure 5F). Two SNPs significantly associated with ACP were detected on chromosomes 1 and 5 (Figure 5G). A total of 35 candidate functional genes that may be related to petal traits were found at the above five SNPs, and 15, 10, and 10 candidate genes related to NPF, CD and ACP, respectively, were found (Supplementary Material 4). There were two SNPs significantly associated with AL detected on chromosomes 5 and 24, respectively (Figure 5H). One SNP significantly associated with TPL was detected on chromosome 3 (Figure 5I). No SNPs significantly associated with TCL and TFL were detected at the 0.01 significant level [–log10(*P*) > 6.67], but at the 0.05 significant level [–log10(*P*) > 5.97], there were five and two SNPs significantly associated with TCL and TFL on chromosomes 9, 12, and 18 (Figures 5J,K). A total of 44 candidate functional genes that may be related to petal traits were found at the above 10 SNPs, and 14, 17, 11, and two candidate genes related to AL, TCL, TFL, and TPL, respectively, were found (Supplementary Material 4).

### PCR Verification

**Figure 6:**
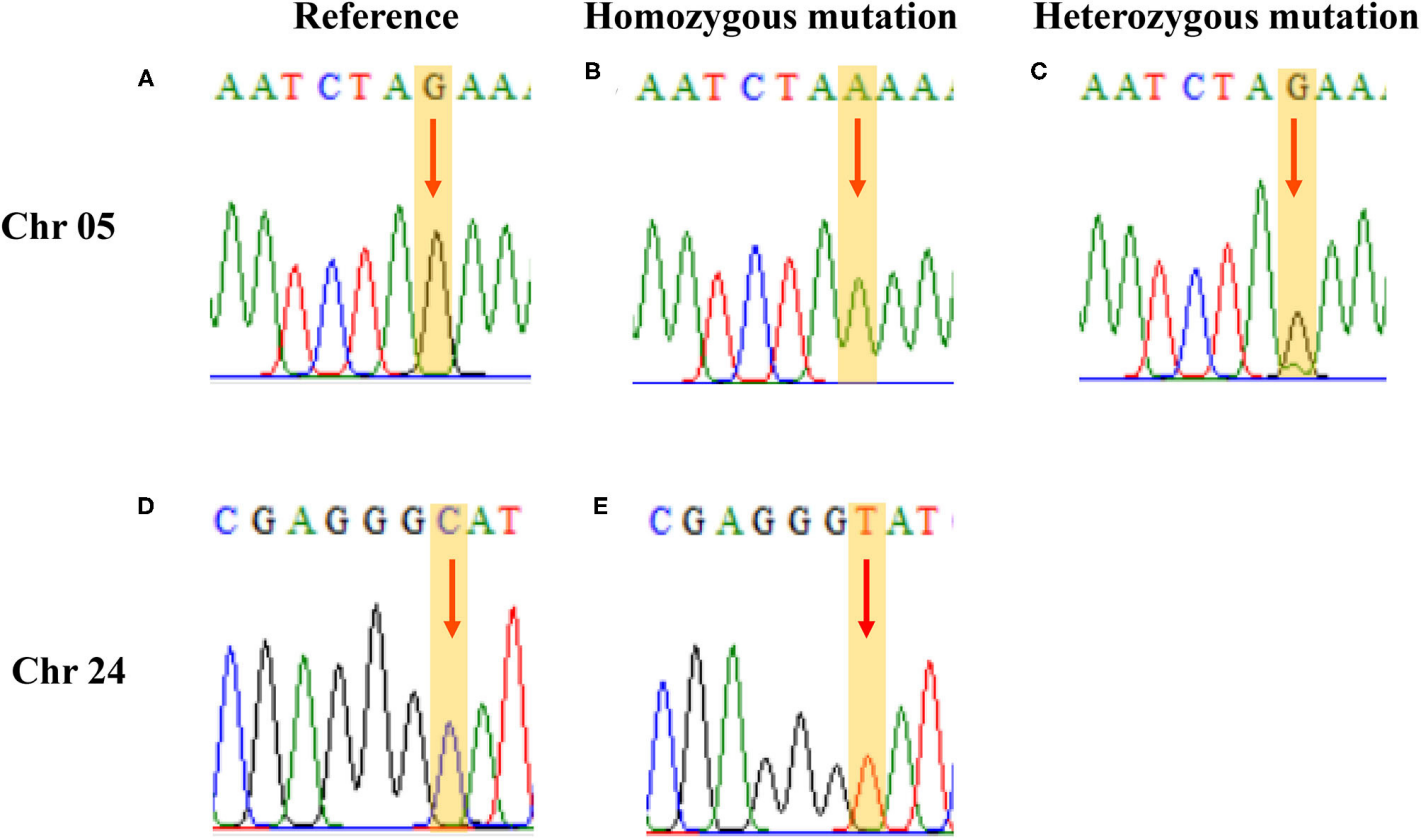
PCR verification results. **(A, D)** the same as the reference; **(B, E)** homozygous mutation; and **(C)** heterozygous mutation.

Two single nucleotide polymorphism sites related to ascorbic acid content in the leaf were selected for verification. Based on the re-sequencing data and SNP positions, primers were designed for verification. The verification results of Sanger sequencing and the re-sequencing screening results have a matching rate of 98.95% (Figure 6 and Supplementary Material 1). Re-sequencing results showed that three of the SNP sites on chromosome 24 were heterozygous mutations, but Sanger sequencing results showed that these sites were consistent with the reference genome and were not heterozygous mutations.

## Discussion

Genetic diversity analysis usually includes phenotypic and molecular diversity, and phenotypic diversity is usually influenced by environment and genes, so molecular diversity analysis results are often more reliable. In this study, the results of molecular clustering analysis based on SNP sites were consistent with the results of geographical distribution. This was also consistent with the expected results. In addition, the genetic diversity and evolution of male and female *A. eriantha* were studied by ISSR, SSR, and other molecular marker techniques (Tang et al., 2014a; Zhong et al., 2018; Liao et al., 2019b). However, because of the few markers, the research results are not in-depth and accurate, and compared with grape, apple, strawberry, banana, and citrus, the development of high-throughput sequencing and large-scale SNP markers in *Actinidia* started late (Zhang et al., 2015). Especially, it was not reported in *A. eriantha*, which greatly restricted the development and utilization of SNP markers in *A. eriantha*. This study enriched the SNP study on *A. eriantha*.

The linkage disequilibrium decay distance in the germplasm material determines the marker density in genome-wide association study analysis. Based on the value of LD attenuation distances, *A. eriantha* has a relatively fast attenuation rate, but is slightly lower than that of grape (10 Kb) (Myles et al., 2011), cassava (3 Kb) (Ramu et al., 2017), and potato (1 Kb) (Simko, 2004). Because of short attenuation distance, it is difficult for low-density markers to cover most alleles. Therefore, the high-density SNP developed in this study based on simplified genome sequencing will be a powerful tool for mining superior alleles. *A. eriantha* has about 691 million bases, and a total of 946,337 SNPs were developed in this study, with about one SNP per 0.73 Kb DNA sequence length, which could be used for subsequent genetic evolution and association analysis. Similar to results reported previously (Lorenzis et al., 2015; Liao et al., 2019b), the materials in this study were closely related to each other, but their phenotypic traits were significantly different. Therefore, the selected germplasm materials meet the requirements of GWAS analysis, and relatively good results are expected to be obtained.

The team of the authors has previously performed a lot of association analyses between traits and markers in *A. eriantha*, and has identified many analytical markers for related traits, which also indicates that part of the phenotypic quantitative traits in *A. eriantha* may be co-regulated by multiple genes (Tang et al., 2014b; Zhong, 2018; Liao et al., 2019b). The results of this study were consistent with this, and the key genes related to flowers and leaves were identified. However, the specific effects of these genes on traits need to be further researched. In addition, in this study, SNPs that had no significant association with some traits at the 0.01 significant level, and it may be that the samples we selected have high genetic diversity, rich variation, and more SNP variation, which may be filtered out because of low rate in the process of sequence alignment, resulting in loss of some SNPs. On the other hand, variation may be filtered out as noise in the screening process because of large differences in various quantitative traits, resulting in loss of association (Zhao, 2018). Because we chose the strict MLM (QK) association model, the population structure and genetic relationship of the sample were considered, and the screening of threshold value was also strict. In addition, the number of significant SNP sites and candidate genes associated was small, and some real sites that reach the threshold value might be missed, resulting in certain false negatives (Alexander et al., 2010; Zhao et al., 2011). The results of this study will provide a theoretical basis for analyzing the regulation mechanism, cultivar improvement, and germplasm innovation of male *A. eriantha-*related traits at the genetic level.

## Data Availability Statement

The original contributions presented in the study are publicly available. This data can be found here: NCBI repository, accession number: PRJNA742212.

## Author Contributions

GL: writing, original draft preparation. MZ, JT, XQ, and XX: collected materials. ZJ: data analysis. QL, DJ, and CH: measured quantitative traits. XX: writing, reviewing and editing. All the authors read and approved the final manuscript.

## Conflict of Interest

The authors declare that the research was conducted in the absence of any commercial or financial relationships that could be construed as a potential conflict of interest.

## Publisher's Note

All claims expressed in this article are solely those of the authors and do not necessarily represent those of their affiliated organizations, or those of the publisher, the editors and the reviewers. Any product that may be evaluated in this article, or claim that may be made by its manufacturer, is not guaranteed or endorsed by the publisher.

## Supplementary Material

Supplementary Material 1 PCR verification primers and the results on 143 *Actinidia eriantha* genotypes.

Supplementary Material 2 Quantitative trait description of 143 *Actinidia eriantha* genotypes used in this study.

Supplementary Material 3 Significant SNP of 11 quantitative traits under different association models.

Supplementary Material 4 SNP and candidate genes significantly associated with flower and leaf traits.

## Abbreviations

ACP: anthocyanin content of petal
AL: ascorbic acid content of leaf
APA: amount of pollen per anther
APF: amount of pollen per flower
AsA: ascorbic acid
CD: corolla diameter
GATK: genome analysis tool kit
GLM: general linear model
GWAS: genome-wide association study
LD: linkage disequilibrium
MLM: mixed linear model
NPF: number of petal per flower
NSF: number of stamen per flower
PV: pollen viability
RAD-seq: restriction-site associated DNA sequencing
SNP: single nucleotide polymorphism
TCL: total chlorophyll content of leaf
TFL: total flavonoid content of leaf
TPL: total phenol content of leaf.
