# Supplementary material for: Genome-Wide Association Studies Provide Insights into the Genetic Determination of Flower and Leaf Traits of Actinidia eriantha
Source: Front Plant Sci. 2021 Aug 20;12:730890. doi: 10.3389/fpls.2021.730890 (PMC8417775; doi:10.3389/fpls.2021.730890)
Supplement: Supplementary Material 2 — Quantitative trait description of 143 Actinidia eriantha genotypes used in this study. [file Table_2.docx]

Supplementary file 2 The table was quantitative traits description of 143 *Actinidia eriantha* genotypes used in this study. The figure shows some germplasm with specific traits, a-d, the petals have higher anthocyanin content; e-h, the flower contains more stamens; the indicators on the leaf are mainly some quality indicators. NPF: the number of petal per flower; NSF: the number of stamen per flower; CD: the corolla diameter; PV: the pollen viability; APA: the amount of pollen per anther; APF: the amount of pollen per flower; ACP: the anthocyanin content of petal; AL: the AsA content of leaf; TPL: the total phenol content of leaf; TFL: the total flavonoid content of leaf; TCL: the total chlorophyll content of leaf. - represents data missing.

| Code | Sample Name | NPF | NSF | CD  (mm) | PV  (%) | APA | APF | ACP  (mg/100g) | AL  (mg/100g) | TPL  (mg/100g) | TFL  (mg/100g) | TCL  (mg/100g) |
| --- | --- | --- | --- | --- | --- | --- | --- | --- | --- | --- | --- | --- |
| 1 | JGS21 | 5.60 | 167.40 | 38.19 | 90.29 | 1.51×10^4^ | 2.53×10^6^ | 53.45 | 128.57 | 3.04×10^4^ | 78.49 | - |
| 2 | JGS22 | 6.00 | 125.40 | 26.71 | 82.82 | 7.92×10^3^ | 9.93×10^5^ | 14.17 | 167.76 | 2.18×10^4^ | 103.88 | 74.66 |
| 3 | JGS26 | 5.17 | 148.00 | 34.59 | 83.66 | 5.63×10^3^ | 8.34×10^5^ | 65.15 | 148.99 | 3.40×10^4^ | 105.64 | 63.96 |
| 4 | JGS31 | 5.80 | 185.50 | 31.50 | - | 1.13×10^4^ | 2.10×10^6^ | 62.25 | 154.28 | 2.28×10^4^ | 68.04 | 87.99 |
| 5 | JGS32 | - | - | - | - | - | - | - | 144.08 | 2.39×10^4^ | 63.72 | 128.43 |
| 6 | JGS33 | 5.00 | 92.00 | 32.84 | - | 6.13×10^3^ | 5.64×10^5^ | 41.07 | 152.76 | 3.56×10^4^ | 117.87 | 91.08 |
| 7 | JGS34 | 5.00 | 153.25 | 30.14 | 22.54 | 4.23×10^3^ | 6.49×10^5^ | 63.54 | 156.90 | 3.94×10^4^ | 155.79 | 74.73 |
| 8 | JGS35 | 5.67 | 125.40 | 28.47 | 89.55 | 1.74×10^4^ | 2.19×10^6^ | 89.53 | 227.74 | 3.18×10^4^ | 102.55 | 124.05 |
| 9 | JGS37 | 6.00 | 154.25 | 31.38 | 81.09 | 3.40×10^3^ | 5.24×10^5^ | 46.53 | 225.48 | 4.84×10^4^ | 127.38 | 84.30 |
| 10 | JGS39 | 5.50 | 157.25 | 29.18 | 93.56 | 4.57×10^3^ | 7.18×10^5^ | 26.71 | 122.79 | 2.52×10^4^ | 50.13 | 51.28 |
| 11 | JGS41 | 5.00 | 156.75 | 33.74 | 89.04 | 6.80×10^3^ | 1.07×10^6^ | 35.71 | 147.81 | 3.14×10^4^ | 113.27 | 82.22 |
| 12 | JGS42 | 5.00 | 154.00 | 31.70 | 85.66 | 5.90×10^3^ | 9.09×10^5^ | 33.80 | 99.93 | 2.80×10^4^ | 97.11 | 83.08 |
| 13 | JGS43 | 5.83 | 180.50 | 30.12 | 20.70 | 1.02×10^4^ | 1.85×10^6^ | 23.20 | 148.68 | 2.42×10^4^ | 75.23 | 101.03 |
| 14 | MGS1 | 5.60 | 177.00 | 34.01 | 75.59 | 1.33×10^4^ | 2.36×10^6^ | 40.38 | 138.51 | 2.72×10^4^ | 126.66 | 110.52 |
| 15 | MGS3 | 5.00 | 186.75 | 45.43 | 85.05 | 1.01×10^4^ | 1.89×10^6^ | 44.17 | 94.76 | 4.10×10^4^ | 161.85 | 96.92 |
| 16 | MGS4 | 5.50 | 218.67 | 31.57 | 85.94 | 5.70×10^3^ | 1.25×10^6^ | 45.08 | 185.63 | 2.89×10^4^ | 99.66 | 102.77 |
| 17 | MGS5 | 5.50 | 167.00 | 36.60 | 85.37 | 8.70×10^3^ | 1.45×10^6^ | 31.53 | 70.07 | 3.28×10^4^ | 120.00 | 102.51 |
| 18 | MGS6 | 5.50 | 183.00 | 38.21 | 93.88 | 7.90×10^3^ | 1.45×10^6^ | 63.48 | 64.61 | 2.63×10^4^ | 95.71 | 72.83 |
| 19 | MGS7 | 5.67 | 229.75 | 35.45 | 68.73 | 1.25×10^4^ | 2.88×10^6^ | 52.54 | 91.75 | 2.43×10^4^ | 93.60 | 73.35 |
| 20 | MGS8 | 5.67 | 98.50 | 37.56 | 90.45 | 1.12×10^4^ | 1.10×10^6^ | 55.83 | 120.90 | 3.90×10^4^ | 160.16 | 72.76 |
| 21 | MGS9 | 5.67 | 162.75 | 34.78 | 89.80 | 8.33×10^3^ | 1.36×10^6^ | 39.36 | 185.08 | 4.88×10^4^ | 215.67 | 96.49 |
| 22 | MGS10 | 6.33 | 210.75 | 31.48 | 94.38 | 5.60×10^3^ | 1.18×10^6^ | 45.14 | 102.94 | 4.01×10^4^ | 152.43 | 111.75 |
| 23 | MGS11 | 4.67 | 216.75 | 31.21 | 95.35 | 6.97×10^3^ | 1.51×10^6^ | 44.47 | 87.23 | 3.82×10^4^ | 140.93 | 78.85 |
| 24 | MGS12 | 5.33 | 144.00 | 29.47 | 90.21 | 8.53×10^3^ | 1.23×10^6^ | 16.24 | 93.33 | 3.07×10^4^ | 174.82 | 74.72 |
| 25 | MGS13 | 5.50 | 294.25 | 38.08 | 86.57 | 5.80×10^3^ | 1.71×10^6^ | 50.60 | 115.69 | 3.17×10^4^ | 156.21 | 82.72 |
| 26 | MGS14 | 5.00 | 129.00 | 32.22 | 78.92 | 3.53×10^3^ | 4.56×10^5^ | 24.73 | 58.85 | 3.51×10^4^ | 155.71 | 31.09 |
| 27 | MGS15 | 5.33 | 229.50 | 33.02 | 90.57 | 1.18×10^4^ | 2.70×10^6^ | 49.46 | 93.97 | 2.86×10^4^ | 209.63 | 94.79 |
| 28 | MGS16 | 5.33 | 128.50 | 32.98 | 83.23 | 1.03×10^4^ | 1.33×10^6^ | 12.08 | 114.65 | 3.04×10^4^ | 127.34 | 72.96 |
| 29 | MGS17 | 5.50 | 165.00 | 32.85 | 81.35 | 5.50×10^3^ | 9.08×10^5^ | 28.73 | 80.50 | 4.24×10^4^ | 279.38 | 85.34 |
| 30 | MGS18 | 5.67 | 149.50 | 30.34 | 86.22 | 7.90×10^3^ | 1.18×10^6^ | 65.94 | 85.36 | 3.29×10^4^ | 213.41 | 87.42 |
| 31 | MGS19 | 5.00 | 180.75 | 31.73 | 85.73 | 8.03×10^3^ | 1.45×10^6^ | 21.88 | 86.69 | 4.26×10^4^ | 160.35 | 76.72 |
| 32 | MGS20 | 5.00 | 138.25 | 29.79 | 78.94 | 1.09×10^4^ | 1.50×10^6^ | 40.01 | 53.65 | 2.87×10^4^ | 74.91 | 74.23 |
| 33 | MGS21 | 5.50 | 135.00 | 25.55 | - | 6.47×10^3^ | 8.73×10^5^ | 35.29 | 139.61 | 3.31×10^4^ | 172.19 | 105.62 |
| 34 | MGS22 | 6.00 | 130.25 | 29.70 | 46.41 | 3.70×10^3^ | 4.82×10^5^ | 55.27 | 165.47 | 4.25×10^4^ | 165.77 | 88.69 |
| 35 | MGS23 | 5.33 | 134.25 | 29.20 | 82.22 | 9.20×10^3^ | 1.24×10^6^ | 31.78 | 184.37 | 4.17×10^4^ | 246.76 | 49.91 |
| 36 | MGS25 | 5.17 | 148.25 | 29.70 | 71.21 | 5.20×10^3^ | 7.71×10^5^ | 60.28 | 58.48 | 3.02×10^4^ | 100.10 | 62.32 |
| 37 | MGS26 | 5.17 | 174.25 | 31.29 | 37.20 | 3.63×10^3^ | 6.33×10^5^ | 64.03 | 145.18 | 2.98×10^4^ | 175.84 | 68.03 |
| 38 | MGS27 | 5.17 | 150.25 | 32.42 | 49.40 | 4.10×10^3^ | 6.16×10^5^ | 51.10 | 104.92 | 3.17×10^4^ | 167.96 | 96.47 |
| 39 | MGS28 | 5.83 | 156.00 | 30.45 | 84.58 | 5.50×10^3^ | 8.58×10^5^ | - | 170.31 | 3.73×10^4^ | 206.81 | 92.50 |
| 40 | MGS29 | 5.00 | 260.75 | 39.62 | 67.86 | 8.37×10^3^ | 2.18×10^6^ | - | 109.12 | 4.86×10^4^ | - | - |
| 41 | MGS30 | 5.67 | 152.67 | 42.34 | 81.12 | 8.97×10^3^ | 1.37×10^6^ | 35.28 | 115.64 | 4.10×10^4^ | 264.08 | 80.27 |
| 42 | MGS31 | 5.33 | 215.00 | 30.08 | 58.05 | 5.07×10^3^ | 1.09×10^6^ | - | 43.33 | 3.69×10^4^ | 73.90 | 97.38 |
| 43 | MGS32 | 4.67 | 180.67 | 45.31 | 51.20 | 9.60×10^3^ | 1.73×10^6^ | 28.08 | 183.54 | 2.66×10^4^ | 148.80 | 62.35 |
| 44 | MGS33 | 5.00 | 109.33 | 22.71 | 47.69 | 3.07×10^3^ | 3.35×10^5^ | - | 61.10 | 1.82×10^4^ | 87.14 | 55.11 |
| 45 | MGS34 | 4.67 | 111.00 | 38.26 | 69.57 | 6.27×10^3^ | 6.96×10^5^ | 19.00 | 236.54 | 2.76×10^4^ | 167.31 | 101.18 |
| 46 | MGS35 | 5.50 | 137.67 | 24.52 | 85.64 | 1.04×10^4^ | 1.43×10^6^ | 21.16 | 296.32 | 3.32×10^4^ | 235.53 | 87.40 |
| 47 | MGS36 | 5.17 | 153.33 | 35.74 | 81.95 | 8.50×10^3^ | 1.30×10^6^ | 31.18 | 179.71 | 2.95×10^4^ | 188.14 | 63.98 |
| 48 | MGS37 | 5.50 | 202.33 | 40.06 | 85.85 | 3.60×10^3^ | 7.28×10^5^ | 22.63 | 365.90 | 4.18×10^4^ | 195.32 | 84.72 |
| 49 | MGS38 | 5.67 | 181.33 | 37.78 | 63.20 | 5.20×10^3^ | 9.43×10^5^ | 36.88 | 224.05 | 3.04×10^4^ | 156.98 | 84.01 |
| 50 | MGS39 | 6.50 | 153.33 | 36.65 | 30.79 | 4.03×10^3^ | 6.18×10^5^ | 27.07 | 170.57 | 2.35×10^4^ | 184.02 | 104.84 |
| 51 | MGS40 | 5.33 | 131.00 | 29.98 | 59.71 | 6.83×10^3^ | 8.95×10^5^ | - | 48.10 | 1.61×10^4^ | 80.47 | 86.62 |
| 52 | MGS41 | 5.00 | 178.67 | 40.88 | 57.84 | 5.13×10^3^ | 9.17×10^5^ | 35.29 | 122.28 | 2.39×10^4^ | 164.82 | 88.14 |
| 53 | MGS43 | 5.00 | 124.00 | 34.95 | 69.23 | 3.60×10^3^ | 4.46×10^5^ | 51.01 | 183.16 | 3.09×10^4^ | 106.20 | 113.48 |
| 54 | MGS44 | 5.33 | 147.00 | 32.79 | 81.81 | 7.00×10^3^ | 1.03×10^6^ | 18.88 | 152.75 | 4.76×10^4^ | 241.45 | 114.05 |
| 55 | MGS51 | 5.67 | 116.00 | 28.89 | 81.53 | 1.04×10^4^ | 1.20×10^6^ | 42.82 | 239.91 | 3.14×10^4^ | 191.08 | 99.42 |
| 56 | MGS52 | 5.67 | 175.25 | 31.95 | 31.93 | - | - | 61.84 | 155.62 | 3.19×10^4^ | 241.54 | 72.23 |
| 57 | MGS53 | 5.67 | 117.50 | 31.25 | 89.03 | 1.01×10^4^ | 1.18×10^6^ | 36.10 | 73.13 | 1.70×10^4^ | 82.08 | 43.36 |
| 58 | MGS54 | 5.17 | 192.00 | 39.83 | 84.84 | 8.63×10^3^ | 1.66×10^6^ | 24.37 | 133.74 | 3.53×10^4^ | 258.31 | 73.85 |
| 59 | MGS55 | 5.00 | 141.50 | 29.97 | 95.56 | 1.29×10^4^ | 1.83×10^6^ | 26.59 | 259.54 | 3.78×10^4^ | 178.28 | 97.56 |
| 60 | MGS57 | 5.67 | 174.00 | 30.41 | 34.14 | 1.22×10^4^ | 2.12×10^6^ | 32.76 | 199.08 | 3.79×10^4^ | 166.57 | 105.00 |
| 61 | MGS58 | 5.33 | 169.75 | 30.81 | 38.52 | 1.25×10^4^ | 2.18×10^6^ | 39.48 | 111.50 | 3.02×10^4^ | 98.99 | 112.07 |
| 62 | MGS59 | 5.67 | 154.00 | 28.20 | 83.08 | 9.33×10^3^ | 1.58×10^6^ | 31.43 | 102.23 | 2.38×10^4^ | 118.41 | 94.76 |
| 63 | MGS60 | 5.50 | 161.00 | 40.30 | 79.90 | 1.14×10^4^ | 1.76×10^6^ | 36.25 | 96.29 | 2.47×10^4^ | 132.12 | 115.67 |
| 64 | MGS61 | 6.00 | 194.00 | 29.86 | 92.68 | 6.27×10^3^ | 1.01×10^6^ | 35.66 | 217.06 | 3.98×10^4^ | 183.19 | 85.60 |
| 65 | MGS62 | 5.83 | 180.00 | 39.34 | 82.96 | 5.33×10^3^ | 1.03×10^6^ | 28.43 | 113.82 | 2.67×10^4^ | 108.19 | 98.49 |
| 66 | MGS63 | 5.67 | 229.25 | 41.69 | 66.57 | 3.97×10^3^ | 7.14×10^5^ | 31.90 | 129.54 | 3.55×10^4^ | 139.68 | 92.37 |
| 67 | MGS64 | 5.67 | 199.00 | 34.53 | 77.91 | 9.73×10^3^ | 1.94×10^6^ | 30.33 | 170.86 | 2.76×10^4^ | 185.07 | 98.00 |
| 68 | MGS65 | 5.83 | 195.50 | 36.75 | 74.81 | 9.60×10^3^ | 1.88×10^6^ | 29.07 | 92.46 | 2.78×10^4^ | 122.76 | 82.55 |
| 69 | MGS66 | 5.50 | 197.75 | 38.10 | 80.25 | 1.24×10^4^ | 2.45×10^6^ | 16.04 | 115.28 | 2.00×10^4^ | 91.56 | 75.72 |
| 70 | MGS67 | 5.33 | 89.50 | 31.43 | 69.32 | 8.17×10^3^ | 7.31×10^5^ | 16.90 | 130.02 | 3.53×10^4^ | 199.46 | 111.45 |
| 71 | MGS68 | 5.33 | 263.67 | 45.42 | 53.81 | 1.18×10^4^ | 3.10×10^6^ | 23.02 | 72.57 | 1.92×10^4^ | 130.54 | 62.47 |
| 72 | MGS69 | 5.60 | 159.33 | 34.15 | 49.06 | 1.01×10^4^ | 1.61×10^6^ | 44.78 | 159.59 | 3.86×10^4^ | 229.54 | 101.91 |
| 73 | MGS70 | 5.83 | 111.00 | - | 54.12 | 2.87×10^3^ | 3.18×10^5^ | 31.98 | 74.23 | 1.21×10^4^ | 130.14 | 60.18 |
| 74 | MGS71 | 5.83 | 176.25 | 39.04 | 60.41 | 9.67×10^3^ | 1.70×10^6^ | 18.33 | 143.11 | 1.88×10^4^ | 98.52 | 65.80 |
| 75 | MGS72 | 5.33 | 211.50 | 37.41 | 82.85 | 3.20×10^3^ | 6.77×10^5^ | 39.44 | 108.74 | 3.28×10^4^ | 196.01 | 91.12 |
| 76 | MGS73 | 5.83 | 188.25 | 37.95 | 46.78 | 2.13×10^3^ | 4.02×10^5^ | 13.42 | 198.24 | 2.27×10^4^ | 144.31 | 118.53 |
| 77 | MGS74 | 5.00 | 189.00 | 40.84 | 81.17 | 8.47×10^3^ | 1.60×10^6^ | 53.59 | 210.59 | 2.83×10^4^ | 163.24 | 92.01 |
| 78 | MGS75 | 5.17 | 119.50 | 29.79 | 35.37 | 8.60×10^3^ | 1.03×10^6^ | 19.87 | 189.82 | 3.21×10^4^ | 140.00 | 104.93 |
| 79 | MGS76 | 5.50 | 147.75 | 36.70 | 24.05 | 5.03×10^3^ | 7.44×10^5^ | 22.29 | 302.14 | 3.64×10^4^ | 136.86 | 78.61 |
| 80 | MGS79 | 5.00 | 269.25 | 31.84 | 71.04 | 1.19×10^4^ | 3.20×10^6^ | 23.36 | 219.41 | 1.84×10^4^ | 128.71 | 107.27 |
| 81 | MGS81 | 5.00 | 148.25 | 35.37 | 73.98 | 5.53×10^3^ | 8.20×10^5^ | 32.12 | 115.92 | 1.90×10^4^ | 155.91 | 108.93 |
| 82 | MGS82 | 5.00 | 153.50 | 30.06 | 74.00 | 9.77×10^3^ | 1.50×10^6^ | 28.95 | 123.75 | 2.68×10^4^ | 158.91 | 104.91 |
| 83 | MGS83 | 5.33 | 141.60 | 30.99 | 62.08 | 8.90×10^3^ | 1.26×10^6^ | 66.70 | 151.38 | 3.40×10^4^ | 164.38 | 121.26 |
| 84 | MGS84 | 5.50 | 229.75 | 43.16 | 46.15 | 6.27×10^3^ | 1.44×10^6^ | 24.55 | 306.41 | 4.52×10^4^ | 291.21 | 95.73 |
| 85 | MGS86 | 5.33 | 170.33 | 30.75 | 85.12 | 1.03×10^4^ | 1.76×10^6^ | 37.20 | 163.07 | 1.73×10^4^ | 138.48 | 125.47 |
| 86 | MGS87 | 5.67 | 206.67 | 27.37 | 57.83 | 9.27×10^3^ | 1.92×10^6^ | 18.71 | 224.85 | 2.19×10^4^ | 161.31 | 117.41 |
| 87 | MGS88 | 5.17 | 170.00 | 28.61 | 75.47 | 6.77×10^3^ | 1.15×10^6^ | 17.16 | 178.46 | 2.81×10^4^ | 176.68 | 123.79 |
| 88 | MGS89 | 5.33 | 149.40 | 30.05 | 56.15 | 1.08×10^4^ | 1.61×10^6^ | 24.25 | 183.50 | 2.73×10^4^ | 180.72 | 141.00 |
| 89 | MGS90 | 5.00 | 139.50 | 34.75 | 36.30 | 3.03×10^3^ | 4.23×10^5^ | 23.19 | 138.30 | 2.94×10^4^ | 193.35 | 114.69 |
| 90 | MGS91 | 5.17 | 162.25 | 27.40 | 80.52 | 6.07×10^3^ | 9.84×10^5^ | 25.66 | 100.01 | 4.06×10^4^ | 201.52 | 79.76 |
| 91 | MGS92 | 5.17 | 152.00 | 35.45 | 26.69 | 3.03×10^3^ | 4.61×10^5^ | 26.27 | 99.35 | 3.64×10^4^ | - | - |
| 92 | MGS93 | 5.50 | 206.67 | 32.98 | 69.07 | 5.73×10^3^ | 1.18×10^6^ | 40.51 | 118.29 | 2.89×10^4^ | 242.54 | 106.87 |
| 93 | MGS94 | 5.67 | 136.75 | 34.43 | 49.86 | 8.93×10^3^ | 1.22×10^6^ | 36.30 | 119.87 | 4.28×10^4^ | 380.49 | 109.70 |
| 94 | MGS100 | 5.33 | 92.33 | 36.47 | 68.69 | 5.93×10^3^ | 5.48×10^5^ | 38.97 | 94.41 | 2.62×10^4^ | 162.24 | 54.50 |
| 95 | MGS101 | 5.00 | 150.33 | 33.76 | 76.48 | 8.87×10^3^ | 1.33×10^6^ | 18.93 | 95.41 | 2.30×10^4^ | 138.17 | 77.15 |
| 96 | MGS102 | 5.00 | 165.33 | 34.50 | 31.32 | 7.67×10^3^ | 1.27×10^6^ | 35.46 | 145.10 | 2.65×10^4^ | 182.29 | 107.89 |
| 97 | MGS103 | 4.67 | 138.33 | 31.12 | 30.77 | 7.27×10^3^ | 1.01×10^6^ | 37.56 | 188.00 | 3.02×10^4^ | 318.76 | 104.02 |
| 98 | MGS104 | 5.33 | 146.00 | 30.54 | - | 3.73×10^3^ | 5.45×10^5^ | 69.66 | 155.25 | 3.26×10^4^ | 230.10 | 102.47 |
| 99 | MGS106 | 5.33 | 100.00 | 31.74 | 58.64 | 7.53×10^3^ | 7.53×10^5^ | 32.69 | 220.56 | 3.12×10^4^ | 165.80 | 109.20 |
| 100 | MGS107 | 5.50 | 157.00 | 37.53 | 66.31 | 7.20×10^3^ | 1.13×10^6^ | 36.44 | 218.95 | 3.81×10^4^ | 172.93 | 121.03 |
| 101 | MGS108 | 5.50 | 142.33 | 34.21 | - | 4.20×10^3^ | 5.98×10^5^ | 36.76 | 156.39 | 2.76×10^4^ | 173.01 | 114.81 |
| 102 | MGS110 | 5.33 | 91.33 | - | - | 7.13×10^3^ | 6.52×10^5^ | 28.35 | 139.13 | 2.57×10^4^ | 117.72 | 142.12 |
| 103 | MGS111 | 5.00 | 150.00 | 38.53 | 27.92 | 8.00×10^3^ | 1.20×10^6^ | 71.84 | 233.33 | 3.83×10^4^ | 104.58 | 83.63 |
| 104 | MGS113 | 5.00 | 111.33 | 31.52 | - | 3.67×10^3^ | 4.08×10^5^ | 18.12 | 89.53 | 2.14×10^4^ | 149.02 | 93.50 |
| 105 | MGS115 | 5.50 | 171.00 | 30.89 | - | 5.94×10^3^ | 1.02×10^6^ | 18.03 | 166.73 | 2.26×10^4^ | 145.13 | 89.07 |
| 106 | MGS116 | 5.67 | 124.00 | 32.49 | 27.78 | 2.60×10^3^ | 3.22×10^5^ | 30.58 | 206.77 | 3.38×10^4^ | 137.20 | 86.52 |
| 107 | MGS117 | 5.33 | 111.67 | 29.68 | - | 6.95×10^3^ | 7.76×10^5^ | 15.23 | 80.60 | 1.95×10^4^ | 124.01 | 117.59 |
| 108 | MGS118 | 5.00 | 105.67 | 35.79 | 55.24 | 6.65×10^3^ | 7.03×10^5^ | 26.45 | 151.93 | 2.76×10^4^ | 116.97 | 83.14 |
| 109 | MGS119 | 5.33 | 137.67 | - | - | 3.27×10^3^ | 4.50×10^5^ | 16.42 | 156.45 | 2.40×10^4^ | 127.47 | 93.09 |
| 110 | MGS120 | 5.00 | 148.33 | 41.54 | 51.43 | 7.75×10^3^ | 1.15×10^6^ | 17.41 | 113.23 | 1.99×10^4^ | 105.75 | 95.07 |
| 111 | MGS121 | 5.00 | 148.00 | 31.81 | 68.19 | 7.07×10^3^ | 1.05×10^6^ | 38.44 | 274.17 | 3.84×10^4^ | 242.87 | 104.23 |
| 112 | MGS122 | 6.00 | 142.00 | 34.02 | - | 2.73×10^3^ | 3.88×10^5^ | 19.50 | 157.74 | 2.80×10^4^ | 151.65 | 110.38 |
| 113 | MGS124 | 6.33 | 174.33 | 37.21 | 38.36 | 5.53×10^3^ | 9.65×10^5^ | 45.21 | 294.04 | 3.64×10^4^ | 176.87 | 132.97 |
| 114 | MGS125 | 6.33 | 123.00 | 28.05 | 33.33 | 2.73×10^3^ | 3.36×10^5^ | 30.10 | 145.27 | 3.41×10^4^ | 210.35 | 123.70 |
| 115 | MGS126 | 5.67 | 109.67 | 31.54 | 36.03 | 3.33×10^3^ | 3.66×10^5^ | 29.17 | 306.72 | 4.04×10^4^ | 285.04 | 86.12 |
| 116 | MGS127 | 5.67 | 211.75 | 27.18 | 71.83 | 8.20×10^3^ | 1.74×10^6^ | 35.29 | 104.63 | 2.97×10^4^ | 162.72 | 106.13 |
| 117 | MGS128 | 5.33 | 103.33 | 29.75 | - | 4.40×10^3^ | 4.55×10^5^ | 26.64 | 113.64 | 3.29×10^4^ | 192.01 | 95.02 |
| 118 | MGS130 | 5.33 | 110.33 | - | 51.90 | 9.55×10^3^ | 1.05×10^6^ | 23.47 | 100.47 | 2.97×10^4^ | 96.18 | 71.02 |
| 119 | WGS1 | 5.33 | 75.67 | 30.08 | 24.95 | 1.22×10^4^ | 9.23×10^5^ | - | 113.51 | 4.06×10^4^ | - | - |
| 120 | WGS2 | 6.00 | 98.00 | 34.85 | 68.09 | 1.52×10^4^ | 1.49×10^6^ | - | 144.26 | 2.84×10^4^ | 72.21 | 75.49 |
| 121 | WGS3 | 6.00 | 251.00 | 37.11 | 44.99 | 1.26×10^4^ | 3.16×10^6^ | - | 153.08 | 2.51×10^4^ | 79.68 | 77.43 |
| 122 | WGS4 | 5.50 | 99.00 | 30.36 | 61.46 | 7.70×10^3^ | 7.62×10^5^ | - | 98.57 | 4.06×10^4^ | - | - |
| 123 | WGS5 | 6.33 | 139.50 | 30.41 | 66.09 | 9.20×10^3^ | 1.28×10^6^ | - | 188.33 | 3.25×10^4^ | 88.01 | 70.50 |
| 124 | WGS6 | 6.00 | 112.50 | 31.18 | 63.93 | 1.15×10^4^ | 1.29×10^6^ | - | 156.93 | 2.77×10^4^ | - | - |
| 125 | WGS8 | 6.00 | 116.00 | 28.98 | 68.15 | 9.67×10^3^ | 1.12×10^6^ | - | 176.82 | 3.90×10^4^ | 107.67 | 70.54 |
| 126 | WGS12 | 5.00 | 101.00 | 27.05 | 47.98 | 1.44×10^4^ | 1.45×10^6^ | - | 166.73 | 2.66×10^4^ | 62.16 | 101.64 |
| 127 | WGS14 | 6.33 | 127.25 | 32.86 | 31.18 | 7.70×10^3^ | 9.80×10^5^ | - | 112.21 | 3.90×10^4^ | - | - |
| 128 | WGS18 | 6.00 | 101.50 | 29.36 | 53.44 | 7.40×10^3^ | 7.51×10^5^ | - | 149.76 | 3.40×10^4^ | 96.19 | 73.89 |
| 129 | WGS19 | 5.50 | 134.80 | 29.50 | 79.00 | 9.03×10^3^ | 1.22×10^6^ | - | 153.27 | 4.15×10^4^ | 79.89 | 73.66 |
| 130 | WGS20 | 5.17 | 119.25 | 28.61 | 44.84 | 1.11×10^4^ | 1.32×10^6^ | - | 166.25 | 2.89×10^4^ | 83.64 | 76.07 |
| 131 | WGS22 | 6.33 | 104.67 | 30.03 | 57.85 | 8.23×10^3^ | 8.62×10^5^ | - | 126.58 | - | - | - |
| 132 | WGS23 | 6.00 | 118.75 | 30.27 | 72.70 | 1.20×10^4^ | 1.43×10^6^ | - | 144.09 | 3.63×10^4^ | 130.23 | 56.48 |
| 133 | WFS7 | 5.00 | 128.75 | 34.31 | 85.36 | 8.13×10^3^ | 1.05×10^6^ | - | 112.17 | 3.00×10^4^ | 96.90 | 59.24 |
| 134 | WFS27 | 5.00 | 169.00 | 35.79 | 80.29 | 9.03×10^3^ | 1.53×10^6^ | 39.76 | 149.84 | 3.51×10^4^ | 192.49 | 87.18 |
| 135 | WFS31 | 5.20 | 161.25 | 35.75 | 91.58 | - | - | 28.13 | 142.83 | 3.95×10^4^ | 242.59 | 54.63 |
| 136 | WFS38 | 5.33 | 134.75 | 31.55 | - | - | - | 54.06 | 167.20 | 3.40×10^4^ | 159.26 | 77.02 |
| 137 | WFS41 | 5.17 | 146.25 | 39.50 | 90.21 | 1.02×10^4^ | 1.50×10^6^ | - | 98.66 | 3.61×10^4^ | 115.02 | 120.59 |
| 138 | WFS44 | 5.00 | 94.00 | 27.91 | 67.67 | 7.07×10^3^ | 6.64×10^5^ | 34.51 | 110.08 | 4.39×10^4^ | 121.40 | 69.53 |
| 139 | WFS47 | 5.00 | 131.67 | 29.89 | 92.30 | 5.87×10^3^ | 7.72×10^5^ | 84.55 | 141.66 | 4.53×10^4^ | - | - |
| 140 | WFS49 | 5.67 | 178.33 | 31.82 | 93.79 | 8.97×10^3^ | 1.60×10^6^ | 47.31 | 234.05 | 3.46×10^4^ | 161.27 | 95.53 |
| 141 | WFS54 | 5.00 | 99.25 | 39.23 | 90.22 | 1.17×10^4^ | 1.16×10^6^ | 18.36 | 101.83 | 2.66×10^4^ | 78.35 | 71.71 |
| 142 | WFS57 | 5.83 | 128.00 | 37.16 | 91.89 | 7.13×10^3^ | 9.13×10^5^ | - | 307.57 | 4.35×10^4^ | 188.16 | 75.24 |
| 143 | WFS58 | 6.00 | 143.67 | 24.61 | 89.98 | 4.87×10^3^ | 6.99×10^5^ | 95.09 | 167.84 | 3.88×10^4^ | 173.05 | 71.15 |
