# Supplementary material for: Genome-Wide Association Studies Provide Insights into the Genetic Determination of Flower and Leaf Traits of Actinidia eriantha
Source: Front Plant Sci. 2021 Aug 20;12:730890. doi: 10.3389/fpls.2021.730890 (PMC8417775; doi:10.3389/fpls.2021.730890)
Supplement: Supplementary Material 3 — Significant SNP of 11 quantitative traits under different association models. [file Table_3.docx]

Supplementary file 3 Significant SNP of 11 quantitative traits under different association models.

| Traits | Significance level | *P* value | GLM | GLM(Q) | MLM(K) | MLM(Q+K) |
| --- | --- | --- | --- | --- | --- | --- |
| NPF | 0.01 | 2.16×10^-7^ | 44 | 2 | 29 | 2 |
|  | 0.05 | 1.08×10^-6^ | 187 | 18 | 137 | 17 |
| NSF | 0.01 | 2.16×10^-7^ | 12 | 0 | 0 | 0 |
|  | 0.05 | 1.08×10^-6^ | 40 | 3 | 4 | 1 |
| CD | 0.01 | 2.16×10^-7^ | 8 | 1 | 1 | 1 |
|  | 0.05 | 1.08×10^-6^ | 31 | 6 | 11 | 3 |
| APF | 0.01 | 2.16×10^-7^ | 1 | 1 | 1 | 1 |
|  | 0.05 | 1.08×10^-6^ | 3 | 6 | 3 | 6 |
| APA | 0.01 | 2.16×10^-7^ | 49 | 3 | 39 | 3 |
|  | 0.05 | 1.08×10^-6^ | 249 | 4 | 195 | 5 |
| PV | 0.01 | 2.16×10^-7^ | 0 | 8 | 0 | 0 |
|  | 0.05 | 1.08×10^-6^ | 4 | 43 | 3 | 1 |
| ACP | 0.01 | 2.16×10^-7^ | 57 | 2 | 46 | 2 |
|  | 0.05 | 1.08×10^-6^ | 195 | 10 | 152 | 10 |
| AL | 0.01 | 2.16×10^-7^ | 1 | 1 | 2 | 2 |
|  | 0.05 | 1.08×10^-6^ | 3 | 10 | 7 | 16 |
| TCL | 0.01 | 2.16×10^-7^ | 1 | 0 | 1 | 0 |
|  | 0.05 | 1.08×10^-6^ | 5 | 0 | 7 | 5 |
| TPL | 0.01 | 2.16×10^-7^ | 1 | 1 | 1 | 1 |
|  | 0.05 | 1.08×10^-6^ | 1 | 3 | 1 | 1 |
| TFL | 0.01 | 2.16×10^-7^ | 10529 | 0 | 788 | 0 |
|  | 0.05 | 1.08×10^-6^ | 20881 | 0 | 819 | 2 |
