# Supplementary material for: Genome-Wide Association Studies Provide Insights into the Genetic Determination of Flower and Leaf Traits of Actinidia eriantha
Source: Front Plant Sci. 2021 Aug 20;12:730890. doi: 10.3389/fpls.2021.730890 (PMC8417775; doi:10.3389/fpls.2021.730890)
Supplement: Supplementary Material 4 — SNP and candidate genes significantly associated with flower and leaf traits. [file Table_4.docx]

Supplementary file 4 SNP and candidate genes significantly associated with flower and leaf traits.

| Traits | Chr | Position | Allele^a^ | *P* value | R^2^(%) | Candidate gene | Description | |
| --- | --- | --- | --- | --- | --- | --- | --- | --- |
| APA | Chr 05 | 13323947  13323952 | A/C  G/C | 1.98×10^-7^ | 18.71 | DTZ79_05g05960;  DTZ79_05g05970;  DTZ79_05g05980;  DTZ79_05g05990;  DTZ79_05g06000;  DTZ79_05g06010;  DTZ79_05g06020;  DTZ79_05g06030;  DTZ79_05g06040;  DTZ79_05g06050;  DTZ79_05g06060;  DTZ79_05g06070 | tRNA pseudouridine synthase B;  Unknown protein;  Genomic DNA, chromosome 3, P1 clone: MMJ24;  GHMP kinase ATP-binding protein, putative;  Beta-1,4-N-acetylglucosaminyltransferase-like protein;  NAC domain protein;  NAC domain protein;  Kinase family protein;  Unknown protein;  Ribosomal RNA-processing protein 8;  Chalcone-flavonone isomerase family protein;  Aldose 1-epimerase, putative | |
| APA  APF | Chr 05 | 21860399 | A/T | 1.67×10^-7^  2.06×10^-7^ | 19.48  20.54 | DTZ79_05g11720;  DTZ79_05g11730 | Sieve element occlusion protein;  Eukaryotic aspartyl protease family protein | |
| PV | Chr 06 | 22519450 | C/T | 8.79×10^-7^ | 15.32 | DTZ79_06g14950;  DTZ79_06g14960;  DTZ79_06g14970;  DTZ79_06g14980 | Coffea canephora DH200=94 genomic scaffold, scaffold_1;  Pentatricopeptide repeat-containing protein;  Homeobox leucine zipper family protein;  Cysteine protease | |
| NSF | Chr 13 | 15414747 | T/C | 3.04×10^-7^ | 20.92 | DTZ79_13g12560;  DTZ79_13g12570;  DTZ79_13g12580;  DTZ79_13g12590;  DTZ79_13g12600;  DTZ79_13g12610 | Enhanced downy mildew 2, putative;  TBC1 domain family member;  Unknown protein;  Transcription initiation factor TFIID subunit 6;  IQ-domain protein;  Nuclear ribonuclease Z | |
| NPF | Chr 19 | 15281450 | G/T | 6.29×10^-8^ | 28.19 | DTZ79_19g06220;  DTZ79_19g06230;  DTZ79_19g06240;  DTZ79_19g06250;  DTZ79_19g06260;DTZ79_19g06270;  DTZ79_19g06280;  DTZ79_19g06290;DTZ79_19g06300 | Haloacid dehalogenase-like hydrolase family protein;  Unknown protein;  Polynucleotidyl transferase, ribonuclease H-like superfamily protein;  LOCATED IN: mitochondrion;  Importin subunit alpha;  Unknown protein;  Agenet domain-containing protein, putative;  Unknown protein;  Neutral/alkaline invertase |  |
| NPF | Chr 22 | 12323585 | C/T | 7.30×10^-8^ | 28.82 | DTZ79_22g04890;  DTZ79_22g04900;  DTZ79_22g04910;  DTZ79_22g04920;  DTZ79_22g04930;  DTZ79_22g04940 | Unknown protein;  Nodulin MtN21 /EamA-like transporter family protein;  Ras-related protein Rab-8A;  Inorganic pyrophosphatase family protein;  Coffea canephora DH200=94 genomic scaffold, scaffold_22;  Histone H2A |  |
| CD | Chr 22 | 21866146 | T/C | 9.72×10^-10^ | 17.00 | DTZ79_22g12270;  DTZ79_22g12280;  DTZ79_22g12290;DTZ79_22g12300;DTZ79_22g12310;DTZ79_22g12320;DTZ79_22g12330;DTZ79_22g12340;DTZ79_22g12350;DTZ79_22g12360 | Coffea canephora DH200=94 genomic scaffold, scaffold_9;  Coffea canephora DH200=94 genomic scaffold, scaffold_12;  Unknown protein;  Unknown protein;  Unknown protein;  Patatin;  Chorismate synthase;  Unknown protein;  Ubiquitin-conjugating enzyme E2;  MLO-like protein |  |
| ACP | Chr 01 | 16955703 | A/G | 9.06×10^-8^ | 23.74 | DTZ79_01g09630;  DTZ79_01g09640;  DTZ79_01g09650;  DTZ79_01g09660;  DTZ79_01g09670;  DTZ79_01g09680 | Terpene cyclase/mutase family member;  Alkaline alpha-galactosidase seed imbibition protein;  Sister chromatid cohesion protein PDS5 like B;  Unknown protein;  Ribosomal protein S5 family protein;  Zinc finger AN1 and C2H2 domain stress-associated protein |  |
| ACP | Chr 05 | 20301771 | C/T | 1.68×10^-7^ | 19.53 | DTZ79_05g10900;DTZ79_05g10910;DTZ79_05g10920;DTZ79_05g10930 | Histone-lysine N-methyltransferase;  BEL1-like homeodomain protein 2;  5'-3' exonuclease family protein;  Flap endonuclease Xni |  |
| AL | Chr 05 | 19338016 | G/A | 2.57×10^-8^ | 20.62 | DTZ79_05g10200;  DTZ79_05g10210;  DTZ79_05g10220;  DTZ79_05g10230 | Heavy metal atpase 1;  FAD-dependent oxidoreductase family protein;  RP/EB family microtubule-associated protein;  Cytochrome P450 | |
| AL | Chr 24 | 13872137 | C/T | 2.50×10^-9^ | 20.25 | DTZ79_24g08360;  DTZ79_24g08370;  DTZ79_24g08380;  DTZ79_24g08390;  DTZ79_24g08400;  DTZ79_24g08410;  DTZ79_24g08420;  DTZ79_24g08430;  DTZ79_24g08440;  DTZ79_24g08450 | Poly(A) RNA polymerase GLD2-like protein;  S-adenosyl-L-methionine-dependent methyltransferases superfamily protein;  S-adenosyl-L-methionine-dependent methyltransferases superfamily protein;  BTB-POZ and MATH domain protein;  Regulator of chromosome condensation (RCC1) family protein;  Receptor-like protein kinase;  Unknown protein;  Eukaryotic translation initiation factor 3 subunit G;  Mannose-1-phosphate guanyltransferase, putative;  CASP-like protein; | |
| TPL | Chr 03 | 19322743 | G/A | 1.81×10^-7^ | 47.27 | DTZ79_03g15410;  DTZ79_03g15420 | Nucleolar protein 10;  Transcription factor ORG2-like protein | |
| TCL | Chr 09 | 4605737 | G/A | 1.04×10^-6^ | 13.29 | DTZ79_09g03940;DTZ79_09g03950;  DTZ79_09g03960;  DTZ79_09g03970;DTZ79_09g03980;DTZ79_09g03990;DTZ79_09g04000;DTZ79_09g04010;  DTZ79_09g04020;DTZ79_09g04030;  DTZ79_09g04040;DTZ79_09g04050;DTZ79_09g04060 | Zinc finger family protein;  Transmembrane and coiled-coil domains protein, putative;  Carbon catabolite repressor protein 4 like 2;  F-box family protein;  Kinase family protein;  Thioredoxin;  Protein trichome birefringence;  Pmr5/Cas1p GDSL/SGNH-like acyl-esterase family protein;  Protein trichome birefringence;  Homeobox associated leucine zipper protein;  Receptor-like kinase;  nudix hydrolase homolog 23;  Lectin receptor kinase | |
| TCL | Chr 12 | 13603928  13603950  13603963  13603985 | G/A  C/T  G/A  C/T | 7.90×10^-7^  1.04×10^-6^  6.64×10^-7^  3.04×10^-7^ | 18.20  18.01  19.34  19.43 | DTZ79_12g08260;  DTZ79_12g08270;  DTZ79_12g08280;DTZ79_12g08290 | RING/U-box superfamily protein;  Interactor of constitutive active ROPs protein, putative;  Coiled-coil domain-containing 94;  Gibberellin 2-oxidase 2 | |
| TFL | Chr 12 | 16989132 | C/A | 6.05×10^-7^ | 12.23 | DTZ79_12g09540;  DTZ79_12g09550;DTZ79_12g09560;DTZ79_12g09570;DTZ79_12g09580;DTZ79_12g09590 | Coffea canephora DH200=94 genomic scaffold, scaffold_60;  Protein disulfide-isomerase like 2-2;  Syntaxin, putative;  Syntaxin of plants 122 protein;  Isocitrate lyase;  Protein yippee-like | |
| TFL | Chr 18 | 14479741 | A/G | 5.99×10^-7^ | 14.45 | DTZ79_18g06530;  DTZ79_18g06540;  DTZ79_18g06550;  DTZ79_18g06560;DTZ79_18g06570 | Pentatricopeptide repeat-containing-like protein;  WRKY transcription factor;  NADH-ubiquinone oxidoreductase-related;  UDP-glycosyltransferase;  UDP-glycosyltransferase | |
