# Supplementary material for: Genome-Wide Association Studies Provide Insights into the Genetic Determination of Flower and Leaf Traits of Actinidia eriantha
Source: Front Plant Sci. 2021 Aug 20;12:730890. doi: 10.3389/fpls.2021.730890 (PMC8417775; doi:10.3389/fpls.2021.730890)
Supplement: Supplementary file 5 [file Data_Sheet_1.ZIP › Supporting information files 1/Chr 05 SNP/Chr 05.docx]

Chr 05 SNP

F: GCTAGTTACTGTAGACTAGT

R: TTGGTCATCTGGAATGCAAT

Product size: 322 bp

Tm: 52℃

>Chr05 SNP-1

GTAGTGTGGCTCTTCTCTTTTTTCCCAAAGACCCCTGGTAACCCATATCCATTTCACCAAATTAGCAATTTGATGAAGTACTCTCTTTCTCTGTCTAGATCACAACAACACGTCCTCCTCTCTCATTCTAGAAATTAGAAATCTAGAAAGGAAATTGATTTCTGCACTCTAAAATTTGATTTCTGCACTCCTATTTGAGTGCAGATATCAACACCATTTCTGCACTCCTATTTTTACAAATTTTGAAGTGCAGAAATTATTTTTCCTTTAGAAATTGCATTCAG

>Chr05 SNP-2

GCGGTTGGTCTCTTCTCTTTTTTCCCAAAGACCCCTGGTAACCCATATCCATTTCACCAAATTAGCAATTTGATGAAGTACTCTCTTTCTCTCTCTAGATCACAACAACAAGTCCTCCTCTCTCATTCTAGAAATTAGAAATCTAGAAAGGAAATTGATTTCTGCACTCTAAAATTTGATTTTTGCACTCCTATTTGAGTGCAGAAATCAACATCATTTCTGCACTCTTACTTTTACAAATTTTGGAGTGCAGAAAATAATTTCCTTTAGAAATTGCATTCCAGATGACCAAAAG

>Chr05 SNP-3

AGTACGGTGGCCTCTTCTCTTTTTTCCCAAAGACCCCTGGTAACCCATATCCATTTCACCAAATTAGCAATTTGATGAAGTACTCTCTTTCTCTCTCTAGATCACAACAACAAGTCCTCCTCTCTCATTCTAGAAATTAGAAATCTAGAAAGGAAATTGATTTCTGCACTCTAAAATTTGATTTTTGCACTCCTATTTGAGTGCAGAAATCAACATCATTTCTGCACCCCTACTTTTACAAATTTTGAAGTGCAGAAAATAATTTCCTTTAAAAATTGGATTCAG

>Chr05 SNP-4

GGACTTACGATCTTCTCTTTTTTCCCAAAGACCCCTGGTAACCCATATCCATTTCACCAAATTAGCAATTTGATGAAGTACTCTCTTTCTCTCTCTAGATCACAACAACAAGTCCTCCTCTCTCATTCTAGAAATTAGAAATCTAGAAAGGAAATTGATTTCTGCACTCTAAAATTTGATTTTTGCACTCCTATTTGAGTGCAGAAATCAACATCATTTCTGCACTCTTACTTTTACAAATTTTGGAGTGCAGAAAATAGTTTCCTTTAGAAATTGCATTCAGG

>Chr05 SNP-5

TTTCTTACGTCTCTTCTCTTTTTTCCCAAAGACCCCTGGTAACCCATATCCATTTCACCAAATTAGCAATTTGATGAAGTACTCTCTTTCTCTCTCTAGATCACAACAATAAGTCCTCCTCTCTCATTCTAGAAATTAGAAATCTAGAAAGGAAATTAATTTCTGCACTCTAAAATTTGATTTTTGCACTCCTATTTGAGTGCAGAAATCAACATCATTTCTGCACTCTTACTTTTACAAATTTTGGAGTGCAGAAAACAATTTCCTTTAGAAATTGCATTCAG

>Chr05 SNP-6

GCTACTGCGATCTTCTCTTTTTTCCCAAAGACCGCTGGTAACCCATATCCATTTCACCAAATTAGCAATTTGATGAAGTACTCTCTTTCTCTCTCTAGATCACAACAACACGTCCTCCTCTCTGATTCTAGAAATTAGAAATCTGGAGAGGAAATTGATTTCTGCACTCTAAAACTTGATTTCTGCGCTCCTATTTGAGTGTATAAATCAACACCATTTCTGCACTCCTACTTTTACAAATTTTGAAGTGCAGAAATTATTTTTCCTTTAAAAATTGCTCCA

>Chr05 SNP-7

GTGTTGCCATCTTCTCTTTTTTCCCAAAGACCGCTGGTAACCCATATCCATTTCACCAAATTAGCAATTTGATGAAGTACTCTCTTTCTCTGTCTAGATCACAACAACACGTCCTCCTCTCTCATTCTAGAAATTAGAAATCTAGAAAGGAAATTGATTTCTGCACTCTAAAATTTGATTTCTGCACTCCTATTTGAGTGCAGATATCAACACCATTTCTGCACTCCTATTTTTACAAATTTTGAAGTGCAGAAATTATTTTTCCTTTAGAAATTGCATTCAGGAATGACCAAAA

>Chr05 SNP-8

ACTCACTGTGGTCTCTTCATCTTTTTTACCAAGACCCCTGGTAACCCATATCCATTTCACCAAATTAGCAATTTGATGAAGTACTCTCTTTCTCTCTCTAGATCACAACAACAAGTCCTCCTCTCTCATTCTAGAAATTAGAAATCTAGAAAGGAAATTGATTTCTGCACTCTAAAATTTGATTTTTGCACTCCTATTTGAGTGCAGAAATCAACATCATTTCTGCACTCTTACTTTTACAAATTTTGGAGTGCAGAAAATAATTTCCTTTAGAAATTGCATTCAGGAA

>Chr05 SNP-9

GGACCTACGATCTTCTCTTTTTTCCCAAAGACCCCTGGTAACCCATATCCATTTCACCAAATTAGCAATTTGATGAAGTACTCTCTTTCTCTGTCTAGATCACAACAACACGTCCTCCTCTCTCATTCTAGAAATTAGAAATCTAGAAAGGAAATTGATTTCTGCACTCTAAAATTTGATTTCTGCACTCCTATTTGAGTGCAGATATCAACACCATTTCTGCACTCCTATTTTTACAAATTTTGAAGTGCAGAAATTATTTTTCCTTTAGAAATTGCATTC

>Chr05 SNP-10

GACTGGCGCTCTTCTCTTTTTTCCCAAAGACCCCTGGTAACCCATATCCATTTCACCAAATTAGCAATTTGATGAAGTACTCTCTTTCTCTGTCTAGATCACAACAACAAGTCCTCCTCTCTCATTCTAGAAATTAGAAATCTAGAAAGGAAATTGATTTCTGCACTCTAAAATTTGATTTTTGCACTCCTATTTGAGTGCAGAAATCAACATCATTTCTGCACTCTTACTTTTACAAATTTTGGAGTGCAGAAAATAATTTCCTTTAGAAATTGCATTCAG

>Chr05 SNP-11

GGTCTTGCCCTCTTCTCTTTTTTCCCAAAGACCCCTGGTAACCCATATCCATTTCACCAAATTAGCAATTTGATGAAGTACTCTCTTTCTCTCTCTAGATCACAACAATAAGTCCTCCTCTCTCATTCTAGAAATTAGAAATCTAGAAAGGAAATTAATTTCTGCACTCTAAAATTTGATTTTTGCACTCCTATTTGAGTGCAGAAATCAACATCATTTCTGCACTCTTACTTTTACAAATTTTGGAGTGCAGAAAACAATTTCCTTTAGAAATTGCATTCAGAA

>Chr05 SNP-12

ATTCTTGGCTCTTCTCTTTTTTCCCAAAGACCGCTGGTAACCCATATCCATTTCACCAAATTAGCAATTTGATGAAGTACTCTCTTTCTCTCTCTAGATCACAACAACACGTCCTCCTCTCTCATTCTAGAAATTAGAAATCTAAAAAGAAAATTGATTTCTGCACTCTAAAATTTAATTTCTGTACTCCTATTTAAGTGCAGAAATCAACGCCATTTCTGCACTCCTACTTTTATAAATTTTGGAGTGCAGAAATTAAATTTCTTTTAGAAATTGCATTCAGGATGACCAAAGGG

>Chr05 SNP-13

TTTTTGCCCTCTTCTCTTTTTTCCCAAAGACCCCTGGTAACCCATATCCATTTCACCAAATTAGCAATTTGATGAAGTACTCTCTTTCTCTCTCTAGATCACAACAACAAGTCCTCCTCTCTCATTCTAGAAATTAGAAATCTAGAAAGGAAATTGATTTCTGCACTCTAAAATTTGATTTTTGCACTCCTATTTGAGTGCAGAAATCAACATCATTTCTGCACTCTTACTTTTACAAATTTTGGAGTGCAGAAAATAATTTCCTTTAGAAATTGCATCCAGGAATGACCAAA

>Chr05 SNP-14

TGACTTCGCTCTTCTCTTTTTTCCCAAAGACCCCTGGTAACCCATATCCATTTCACCAAATTAGCAATTTGATGAAGTACTCTCTTTCTCTGTCTAGATCACAACAACACGTCCTCCTCTCTCATTCTAGAAATTAGAAATCTAGAAAGGAAATTGATTTCTGCACTCTAAAATTTGATTTCTGCACTCCTATTTGAGTGCAGATATCAACACCATTTCTGCACTCCTATTTTTACAAATTTTGAAGTGCAGAAATTATTTTTCCTTTAGAAATTGCATTCAGAATGACCAAAGG

>Chr05 SNP-15

GTAGTGTGGCTCTTCTCTTTTTTCCCAAAGACCCCTGGTAACCCATATCCATTTCACCAAATTAGCAATTTGATGAAGTACTCTCTTTCTCTGTCTAGATCACAACAACACGTCCTCCTCTCTCATTCTAGAAATTAGAAATCTAGAAAGGAAATTGATTTCTGCACTCTAAAATTTGATTTCTGCACTCCTATTTGAGTGCAGATATCAACACCATTTCTGCACTCCTATTTTTACAAATTTTGAAGTGCAGAAATTATTTTTCCTTTAGAAATTGCATTCAG

>Chr05 SNP-16

GTTTTTTTCATCTTCTCTTTTTTCCCAAAGACCCCTGGTAACCCATATCCATTTCACCAAATTAGCAATTTGATGAAGTACTCTCTTTCTCTGTCTAGATCACAACAACACGTCCTCCTCTCTCATTCTAGAAATTAGAAATCTAGAAAGGAAATTGATTTCTGCACTCTAAAATTTGATTTCTGCACTCCTATTTGAGTGCAGATATCAACACCATTTCTGCACTCCTATTTTTACAAATTTTGAAGTGCAGAAATTATTTTTCCTTTAGAAATTGCATTC

>Chr05 SNP-17

ATCTTTTTGGCTCTTCTCTTTTTTCCCAAAGACCCCTGGTAACCCATATCCATTTCACCAAATTAGCAATTTGATGAAGTACTCTCTTTCTCTGTCTAGATCACAACAACACGTCCTCCTCTCTCATTCTAGAAATTAGAAATCTAGAAAGGAAATTGATTTCTGCACTCTAAAATTTGATTTCTGCACTCCTATTTGAGTGCAGATATCAACACCATTTCTGCACTCCTATTTTTACAAATTTTGAAGTGCAGAAATTATTTTTCCTTTAGAAATTGCATTCAGGAATGACCAA

>Chr05 SNP-18

GACTTATCCTCTTCTCTTTTTTCCCAAAGACCGCTGGTAACCCATATCCATTTCACCAAATTAGCAATTTGATGAAGTACTCTCTTTCTCTGTCTAGATCACAACAACACGTCCTCCTCTCTCATTCTAGAAATTAGAAATCTAGAGAGGAAATTGATTTCTGCACTCTAAAACTTGATTTCTGCGCTCCTATTTGAGTGCATATATCAACACCATTTCTGCACTCCTACTTTTACAAATTTTGAAGTGCAGAAATTATTTTTCCTTTAGAAATTGCATTCAGGAATGACCAAAA

>Chr05 SNP-19

TTGTTTGCCTCTTCTCTTTTTTCCCAAAGACCGCTGGTAACCCATATCCATTTCACCAAATTAGCAATTTGATGAAGTACTCTCTTTCTCTGTCTAGATCACAACAACATGTCCTCCTCTCTCGTTCTAGAAATTAGAAATCTGGAAAGAAAATTGATTTCTGCACTCTAAAATTTGATTTCTGCACTCTTATTTGAGTGCAGAAATCAACACCATTTCTGCACTCCTATTTTTACAAATTTTGAAGTGCAGAAATTATTTTTCCTTTAGAAATTGCATTCAGA

>Chr05 SNP-20

CGCTTACCCTCTTCTCTTTTTTCCCAAAGACCCCTGGTAACCCATATCCATTTCACCAAATTAGCAATTTGATGAAGTACTCTCTTTCTCTGTCTAGATCACAACAACACGTCCTCCTCTCTCATTCTAGAAATTAGAAATCTAGAAAGGAAATTGATTTCTGCACTCTAAAATTTGATTTCTGCACTCCTATTTGAGTGCAGATATCAACACCATTTCTGCACTCCTATTTTTACAAATTTTGAAGTGCAGAAATTATTTTTCCTTTAGAAATTGCATTCAG

>Chr05 SNP-21

CTTTTACGCTCTTCTCTTTTTTCCCAAAGACCCCTGGTAACCCATATCCATTTCACCAAATTAGCAATTTGATGAAGTACTCTCTTTCTCTGTCTAGATCACAACAACACGTCCTCCTCTCTCATTCTAGAAATTAGAAATCTAGAAAGGAAATTGATTTCTGCACTCTAAAATTTGATTTCTGCACTCCTATTTGAGTGCAGATATCAACGCCATTTCTGCACTCCTATTTTTACAAATTTTGAAGTGCAGAAATTATTTTTCCTTTAGAAATTGCATTCAGGAATGACCAAAG

>Chr05 SNP-22

GTGTTACCCTCTTCTCTTTTTTCCCAAAGACCCCTGGTAACCCATATCCATTTCACCAAATTAGCAATTTGATGAAGTACTCTCTTTCTCTGTCTAGATCACAACAACACGTCCTCCTCTCTCATTCTAGAAATTAGAAATCTAGAAAGGAAATTGATTTCTGCACTCTAAAATTTGATTTCTGCACTCCTATTTGAGTGCAGATATCAACACCATTTCTGCACTCCTATTTTTACAAATTTTGAAGTGCAGAAATTATTTTTCCTTTAGAAATTGCATTCAG

>Chr05 SNP-23

TTGTTTGCTCTTCTCTTTTTTCCCAAAGACCGCTGGTAACCCATATCCATTTCACCAAATTAGCAATTTGATGAAGTACTCTCTTTCTCTGTCTAGATCACAACAACACGTCCTCCTCTCTCATTCTAGAAATTAGAAATCTGGAAAGGAAATTGATTTCTGCACTCTAAAATTTGATTTCTGCACTCCTATTTGAGTGTAGAAATCAACACCATTTCTGCACTCCTATTTTTACAAATTTTGAAGTGCAGAAATTATTTTTCCTTTAAAAATGGCATCCA

>Chr05 SNP-24

GCGTACTATCTTCCTCTTTTTTACCAAAGACCCCAGGTAACCCATATCCATTTCACCAAATTAGCAATTTGATGAAGTACTCTCTTTCTCTGTCTAGATCACAACAACACGTCCTCCTCTCTGATTCTAGAAATTAGAAATCTGGAAAGGAAATTGATTTCTGCACTCTAAAATTTGATTTCTGCACTCCTATTTGAGTGTAGATATCAACACCATTTCTGCACTCCTATTTTTACAAATTTTGAAGTGCAGAAATTATTTTTCTTTTAAAAATGGTC

>Chr05 SNP-25

GCGGTACGATCTTCCTCTTTTTTCCCAAAGACCGCTGGTAACCCATATCCATTTCACCAAATTAGCAATTTGATGAAGTACTCTCTTTCTCTCTCTAGATCACAACAACACGTCCTCCTCTCTGGTTCTAGAAATTAGAAATCTGGAAAGAAAATTGATTTCTGCACTCTAAAATTTGATTTCTGCACTCCTATTTGAGTATAGAAATCAACGCCATTTCTGCACTCCTACTTTTACAAATTTTGGAGTGCAGAAAACAATTTCCTTTAGAAATTGCTCCGAAAA

>Chr05 SNP-26

CTCGTTACGCTCTTCTCTTTTTTCCCAAAGACCGCTGGTAACCCATATCCATTTCACCAAATTAGCAATTTGATGAAGTACTCTCTTTCTCTGTCTAGATCACAACAACACGTCCTCCTCTCTGATTCTAGAAATTAGAAATCTGGAAAGGAAATTGATTTCTGCACTCTAAAATTTGATTTCTGCACTCCTATTTGAGTGTAGAAATCAACACCATTTCTGCACTCCTACTTTTACAAATTTTGAAGTGCAGAAAACAATTTTCTTTTGGAATTTGCTTAAAA

>Chr05 SNP-27

GTACTATCTTCTCTTTTTTCCCAAAGACCCCTGGTAACCCATATCCATTTCACCAAATTAGCAATTTGATGAAGTACTCTCTTTCTCTGTCTAGATCACAACAACACGTCCTCCTCTCTCATTCTAGAAATTAGAAATCTAGAAAGGAAATTGATTTCTGCACTCTAAAATTTGATTTCTGCACTCCTATTTGAGTGCAGATATCAACACCATTTCTGCACTCCTATTTTTACAAATTTTGAAGTGCAGAAATTATTTTTCCTTTAGAAATTGCTTCA

>Chr05 SNP-28

CTCTTGTGCTCTTCATCTTTTTTCCCAAAGACCGCTGGTAACCCATATCCATTTCACCAAATTAGCAATTTGATGAAGTACTCTCTTTCTCTGTCTAGATCACAACAACACGTCCTCCTCTCTGATTCTAGAAATTAGAAATCTGGAAAGGAAATTGATTTCTGCACTCTAAAATTTGATTTCTGCACTCCTATTTGAGTGTAGAAATCAACACCATTTCTGCACTCCTACTTTTACAAATTTTGAAGTGCAGAAAACAATTTTCTTTTAGAATTTCCATCAAA

>Chr05 SNP-29

GCTACTGCGATCTTCTCTTTTTTCCCAAAGACCGCTGGTAACCCATATCCATTTCACCAAATTAGCAATTTGATGAAGTACTCTCTTTCTCTCTCTAGATCACAACAACACGTCCTCCTCTCTGATTCTAGAAATTAGAAATCTGGAGAGGAAATTGATTTCTGCACTCTAAAACTTGATTTCTGCGCTCCTATTTGAGTGTATAAATCAACACCATTTCTGCACTCCTACTTTTACAAATTTTGAAGTGCAGAAATTATTTTTCCTTTAAAAATTGCTCCA

>Chr05 SNP-30

GTGTGTGGCTCTTCTCTTTTTTCCCAAAGACCGCTGGTAACCCATATCCATTTCACCAAATTAGCAATTTGATGAAGTACTCTCTTTCTCTCTCTAGATCACAACAACACGTCCTCCTCTCTCGTTCTAGAAATTAGAAATCTGGAGAGAAAATTGATTTCTGCACTCTAAAACTTGATTTCTGCGCTCCTATTTGAGTGCATAAATCAACACAATTTCTGCACTCCTACTTTTACAAATTTTGAAGTGCAGAAATTATTTTTCCTTTAGAAATTGCATCCAGGAATGACCAAA

>Chr05 SNP-31

GGCTTGCGACTCTTCTCTTTTTTCCCAAAGACCGCTGGTAACCCATATCCATTTCACCAAATTAGCAATTTGATGAAGTACTCTCTTTCTCTGTCTAGATCACAACAACACGTCCTCCTCTCTGATTCTAGAAATTAGAAATCTGGAAAGGAAATTGATTTCTGCACTCTAAAATTTGATTTCTGCACTCCTATTTGAGTGTAGAAATCAACACCATTTCTGCACTCCTATTTTTACAAATTTTGAAGTGCAGAAATTATTTTTCCTTAAAAAATGGCATAAA

>Chr05 SNP-32

TTTTTTTTTCCTCTTCTCTTTTTTCCCAAAGACCCCTGGTAACCCATATCCATTTCACCAAATTAGCAATTTGATGAAGTACTCTCTTTCTCTCTCTAGATCACAACAATAAGTCCTCCTCTCTCATTCTAGAAATTAGAAATCTAGAAAGGAAATTAATTTCTGCACTCTAAAATTTGATTTTTGCACTCCTATTTGAGTGCAGAAATCAACATCATTTCTGCACCCCTACTTTTACAAATTTTGGAGTGCAGAAAACAATTTCCTTTAAAAATTGCATTCCA

>Chr05 SNP-33

GGCCTTACGCTCTTCTCTTTTTTCCCAAAGACCCCTGGTAACCCATATCCATTTCACCAAATTAGCAATTTGATGAAGTACTCTCTTTCTCTGTCTAGATCACAACAACACGTCCTCCTCTCTCATTCTAGAAATTAGAAATCTAGAAAGGAAATTGATTTCTGCACTCTAAAATTTGATTTCTGCACTCCTATTTGAGTGCAGATATCAACACCATTTCTGCACTCCTATTTTTACAAATTTTGAAGTGCAGAAATTATTTTTCCTTTAGAAATTGCTTCA

>Chr05 SNP-34

CACTTATGCTCTTCTCTTTTTTCCCAAAGACCGCTGGTAACCCATATCCATTTCACCAAATTAGCAATTTGATGAAGTACTCTCTTTCTCTGTCTAGATCACAACAACACGTCCTCCTCTCTGATTCTAGAAATTAGAAATCTGGAAAGGAAATTGATTTCTGCACTCTAAAATTTGATTTCTGCACTCCTATTTGAGTATAGAAATCAACACCATTTCTGCACTCCTACTTTTACAAATTTTGAAGTGCAGAAAACAATTTTCTTTTAGAAATTGCATCAA

>Chr05 SNP-35

GGGTTACGCTCTTCTCTTTTTTCCCAAAGACCCCTGGTAACCCATATCCATTTCACCAAATTAGCAATTTGATGAAGTACTCTCTTTCTCTGTCTAGATCACAACAACACGTCCTCCTCTCTCATTCTAGAAATTAGAAATCTAGAAAGGAAATTGATTTCTGCACTCTAAAATTTGATTTCTGCACTCCTATTTGAGTGCAGATATCAACACCATTTCTGCACTCCTATTTTTACAAATTTTGAAGTGCAGAAATTATTTTTCCTTTAGAAATTGCATTCAG

>Chr05 SNP-36

GTGTGTGGCTCTTCTCTTTTTTCCCAAAGACCGCTGGTAACCCATATCCATTTCACCAAATTAGCAATTTGATGAAGTACTCTCTTTCTCTCTCTAGATCACAACAACACGTCCTCCTCTCTCGTTCTAGAAATTAGAAATCTGGAGAGAAAATTGATTTCTGCACTCTAAAACTTGATTTCTGCGCTCCTATTTGAGTGCATAAATCAACACAATTTCTGCACTCCTACTTTTACAAATTTTGAAGTGCAGAAATTATTTTTCCTTTAGAAATTGCATCCAGGAATGACCAAA

>Chr05 SNP-37

GGCATGTACTCAGTCTTTCTCTTTTTTCCCAAAGACCGCTGGTAACCCATATCCATTTCACCAAATTAGCAATTTGATGAAGTACTCTCTTTCTCTGTCTAGATCACAACAACACGTCCTCCTCTCTCATTCTAGAAATTAGAAATCTAGAGAGGAAATTGATTTCTGCACTCTAAAACTTGATTTCTGCGCTCCTATTTGAGTGCATATATCAACACCATTTCTGCACTCCTACTTTTACAAATTTTGAAGTGCAGAAATTATTTTTCCTTTAGAAATTGGG

>Chr05 SNP-38

GACTTATGCTCTTCTCTTTTTTCCCAAAGACCCCTGGTAACCCATATCCATTTCACCAAATTAGCAATTTGATGAAGTACTCTCTTTCTCTGTCTAGATCACAACAACACGTCCTCCTCTCTCATTCTAGAAATTAGAAATCTAGAAAGGAAATTGATTTCTGCACTCTAAAATTTGATTTCTGCACTCCTATTTGAGTGCAGATATCAACACCATTTCTGCACTCCTATTTTTACAAATTTTGAAGTGCAGAAATTATTTTTCCTTTAGAAATTGCATTCAGAATGACCAAAAT

>Chr05 SNP-39

GGTCTGCGCTCTTCTCTTTTTTCCCAAAGACCCCTGGTAACCCATATCCATTTCACCAAATTAGCAATTTGATGAAGTACTCTCTTTCTCTGTCTAGATCACAACAACACGTCCTCCTCTCTCATTCTAGAAATTAGAAATCTAGAAAGGAAATTGATTTCTGCACTCTAAAATTTGATTTCTGCACTCCTATTTGAGTGCAGATATCAACACCATTTCTGCACTCCTATTTTTACAAATTTTGAAGTGCAGAAATTATTTTTCCTTTAGAAATTGCATTCAG

>Chr05 SNP-40

TATACGTTTACTCTTCTCTTTTTTCCCAAAGACCCCTGGTAACCCATATCCATTTCACCAAATTAGCAATTTGATGAAGTACTCTCTTTCTCTGTCTAGATCACAACAACACGTCCTCCTCTCTCATTCTAGAAATTAGAAATCTAGAAAGGAAATTGATTTCTGCACTCTAAAATTTGATTTCTGCACTCCTATTTGAGTGCAGATATCAACACCATTTCTGCACTCCTATTTTTACAAATTTTGAAGTGCAGAAATTATTTTTCCTTTAGAAATTGCATTCAG

>Chr05 SNP-41

GGATTTGCATCTTCTCTTTTTTCCCAAAGACCGCTGGTAACCCATATCCATTTCACCAAATTAGCAATTTGATGAAGTACTCTCTTTCTCTCTCTAGATCACAACAACACGTCCTCCTCTCTCATTCTAGAAATTAGAAATCTAAAAAGAAAATTGATTTCTGCACTCTAAAATTTAATTTCTGTACTCCTATTTAAGTGCAGAAATCAACGCCATTTCTGCACTCCTACTTTTATAAATTTTGGAGTGCAGAAATTAAATTTCTTTTAGAAATTGCATTCAGGAATGACCAAA

>Chr05 SNP-42

ATTCTTGGCTCTTCTCTTTTTTCCCAAAGACCGCTGGTAACCCATATCCATTTCACCAAATTAGCAATTTGATGAAGTACTCTCTTTCTCTCTCTAGATCACAACAACACGTCCTCCTCTCTCATTCTAGAAATTAGAAATCTAAAAAGAAAATTGATTTCTGCACTCTAAAATTTAATTTCTGTACTCCTATTTAAGTGCAGAAATCAACGCCATTTCTGCACTCCTACTTTTATAAATTTTGGAGTGCAGAAATTAAATTTCTTTTAGAAATTGCATTCAGGATGACCAAAGGG

>Chr05 SNP-43

GTGTTACCCTCTTCTCTTTTTTCCCAAAGACCCCTGGTAACCCATATCCATTTCACCAAATTAGCAATTTGATGAAGTACTCTCTTTCTCTGTCTAGATCACAACAACACGTCCTCCTCTCTCATTCTAGAAATTAGAAATCTAGAAAGGAAATTGATTTCTGCACTCTAAAATTTGATTTCTGCACTCCTATTTGAGTGCAGATATCAACACCATTTCTGCACTCCTATTTTTACAAATTTTGAAGTGCAGAAATTATTTTTCCTTTAGAAATTGCATTCAGAAATGACCAAAG

>Chr05 SNP-44

CTTTTTGCCTCTTCTCTTTTTTCCCAAAGACCGCTGGTAACCCATATCCATTTCACCAAATTAGCAATTTGATGAAGTACTCTCTTTCTCTGTCTAGATCACAACAACACGTCCTCCTCTCTGATTCTAGAAATTAGAAATCTGGAAAGGAAATTGATTTCTGCACTCTAAAATTTGATTTCTGCACTCCTATTTGAGTGTAGAAATCAACACCATTTCTGCACTCCTATTTTTACAAATTTTGAAGTGCAGAAATTATTTTTCCTTAAAAAATGGCATCCGGAATGACCAAA

>Chr05 SNP-45

TTTCCTTCGCTCTTCTCTTTTTTCCCAAAGACCGCTGGTAACCCATATCCATTTCACCAAATTAGCAATTTGATGAAGTACTCTCTTTCTCTGTCTAGATCACAACAACACGTCCTCCTCTCTCATTCTAGAAATTAGAAATCTAGAAAGGAAATTGATTTCTGCACTCTAAAATTTGATTTCTGCACTCCTATTTGAGTGCAGATATCAACACCATTTCTGCACTCCTATTTTTACAAATTTTGAAGTGCAGAAATTATTTTTCCTTTAGAAATTGCATTCAGAAATGACCAAAGGG

>Chr05 SNP-46

ATTCTTGGCTCTTCTCTTTTTTCCCAAAGACCGCTGGTAACCCATATCCATTTCACCAAATTAGCAATTTGATGAAGTACTCTCTTTCTCTCTCTAGATCACAACAACACGTCCTCCTCTCTCATTCTAGAAATTAGAAATCTAAAAAGAAAATTGATTTCTGCACTCTAAAATTTAATTTCTGTACTCCTATTTAAGTGCAGAAATCAACGCCATTTCTGCACTCCTACTTTTATAAATTTTGGAGTGCAGAAATTAAATTTCTTTTAGAAATTGCATTCAGGATGACCAAAGGG

>Chr05 SNP-47

TTTGTGTGCTCTTCTCTTTTTTCCCAAAGACCCCTGGTAACCCATATCCATTTCACCAAATTAGCAATTTGATGAAGTACTCTCTTTCTCTGTCTAGATCACAACAACACGTCCTCCTCTCTCATTCTAGAAATTAGAAATCTAGAAAGGAAATTGATTTCTGCACTCTAAAATTTGATTTCTGCACTCCTATTTGAGTGCAGATATCAACACCATTTCTGCACTCCTATTTTTACAAATTTTGAAGTGCAGAAATTATTTTTCCTTTAGAAATTGCATTCAGGAATGACCAAAA

>Chr05 SNP-48

AGCTACTATTGCTATCTCTTTTTTCACCAAAGACCCCTGGTAACCCATATCCATTTCACCAAATTAGCAATTTGATGAAGTACTCTCTTTCTCTGTCTAGATCACAACAACACGTCCTCCTCTCTCATTCTAGAAATTAGAAATCTAGAAAGGAAATTGATTTCTGCACTCTAAAATTTGATTTCTGCACTCCTATTTGAGTGCAGATATCAACACCATTTCTGCACTCCTATTTTTACAAATTTTGAAGTGCAGAAATTATTTTTCCTTTAGAAATTGCATTCCAGATGACCAAAGCGCCCC

>Chr05 SNP-49

ATTTGTTTGACTCTTCTCTTTTTTCCCAAAGACCCCTGGTAACCCATATCCATTTCACCAAATTAGCAATTTGATGAAGTACTCTCTTTCTCTGTCTAGATCACAACAACACGTCCTCCTCTCTCATTCTAGAAATTAGAAATCTAGAAAGGAAATTGATTTCTGCACTCTAAAATTTGATTTCTGCACTCCTATTTGAGTGCAGATATCAACACCATTTCTGCACTCCTATTTTTACAAATTTTGAAGTGCAGAAATTATTTTTCCTTTAGAAATTGCATTCAGG

>Chr05 SNP-50

AGACCTTTTATCTTCTCTTTTTTCCCAAAGACCCCTGGTAACCCATATCCATTTCACCAAATTAGCAATTTGATGAAGTACTCTCTTTCTCTGTCTAGATCACAACAACACGTCCTCCTCTCTCATTCTAGAAATTAGAAATCTAGAAAGGAAATTGATTTCTGCACTCTAAAATTTGATTTCTGCACTCCTATTTGAGTGCAGATATCAACACCATTTCTGCACTCCTATTTTTACAAATTTTGAAGTGCAGAAATTATTTTTCCTTTAGAAATTGCATTCAG

>Chr05 SNP-51

GGATTATGCTCTTCTCTTTTTTCCCAAAGACCCCTGGTAACCCATATCCATTTCACCAAATTAGCAATTTGATGAAGTACTCTCTTTCTCTGTCTAGATCACAACAACACGTCCTCCTCTCTCATTCTAGAAATTAGAAATCTAGAAAGGAAATTGATTTCTGCACTCTAAAATTTGATTTCTGCACTCCTATTTGAGTGCAGATATCAACACCATTTCTGCACTCCTATTTTTACAAATTTTGAAGTGCAGAAATTATTTTTCCTTTAGAAATTGCATTCAG

>Chr05 SNP-52

GGTTTTCCTCTTCTCTTTTTTCCCAAAGACCGCTGGTAACCCATATCCATTTCACCAAATTAGCAATTTGATGAAGTACTGTCTTTCTCTCTCTAGATCACAACAACACGTCCTCCTCTCTCATTCTAGAAATTAGAAATCTAAAAAGAAAATTGATTTCTGCACTCTAAAATTTAATTTCTGCACTCCTATTTAAGTGCAGAAATCAACGCCATTTCTGCACTCCTACTTTTATAAGTTTTGGAGTGCAGAAATTAAATTTCCTTTAGAAATTGCATTCCAGATGACCAAAAC

>Chr05 SNP-53

TTCTTTACCCTCTTCTCTTTTTTCCCAAAGACCGCTGGTAACCCATATCCATTTCACCAAATTAGCAATTTGATGAAGTACTCTCTTTCTCTGTCTAGATCACAACAACACGTCCTCCTCTCTCATTCTAGAAATTAGAAATCTAGAAAGGAAATTGATTTCTGCACTCTAAAATTTGATTTCTGCACTCCTATTTGAGTGCAGATATCAACACCATTTCTGCACTCCTATTTTTACAAATTTTGAAGTGCAGAAATTATTTTTCCTTTAGAAATTGCATTCAGGAATGACCAAAGC

>Chr05 SNP-54

GACCGTACCCTCTTCTCTTTTTTCCCAAAGACCGCTGGTAACCCATATCCATTTCACCAAATTAGCAATTTGATGAAGTACTCTCTTTCTCTCTCTAGATCACAACAACATGTCCTCCTCTCTCGTTCTAGAAATTAGAAATCTGGAAAGGAAATTGATTTCTGCACTCTAAAATTTGATTTCTGCACTCTTATTTGAGTGCAGAAATCAACACCATTTCTGCACCCCTACTTTTACAAATTTTGAAGTGCAGAAATTATTTTTCCTTTAGAAATTGCATTCA

>Chr05 SNP-55

GGCTTTACGCTCTTCTCTTTTTTCCCAAAGACCGCTGGTAACCCATATCCATTTCACCAAATTAGCAATTTGATGAAGTACTCTCTTTCTCTCTCTAGATCACAACAACATGTCCTCCTCTCTGGTTCTAGAAATTAGAAATCTGGAAAGAAAATTGATTTCTGCACTCTAAAATTTGATTTCTGCACTCTTATTTGAGTATAGAAATCAACACCATTTCTGCACCCCTACTTTTACAAATTTTGAAGTGCAGAAAACAATTTTCTTTTAGGATTTGCTTCAA

>Chr05 SNP-56

ACTTTCTGTTATCTTCTCTTTTTTCCCAAAGACCGCTGGTAACCCATATCCATTTCACCAAATTAGCAATTTGATGAAGTACTCTCTTTCTCTCTCTAGATCACAACAACATGTCCTCCTCTCTCGTTCTAGAAATTAGAAATCTGGAAAGAAAATTGATTTCTGCACTCTAAAATTTGATTTCTGCACTCCTATTTGAGTGCAGAAATCAACACCATTTCTGCACCCCTACTTTTACAAATTTTGAAGTGCAGAAATTATTTTTCCTTTAAAAATGGCATTAAGGATGACCAAA

>Chr05 SNP-57

TTAGTATTGATCTTCTCTTTTTTCCCAAAGACCGCTGGTAACCCATATCCATTTCACCAAATTAGCAATTTGATGAAGTACTCTCTTTCTCTGTCTAGATCACAACAACACGTCCTCCTCTCTCATTCTAGAAATTAGAAATCTGGAAAGGAAATTGATTTCTGCACTCTAAAATTTGATTTCTGCACTCCTATTTGAGTGTAGAAATCAACACCATTTCTGCACTCCTACTTTTACAAATTTTGAAGTGCAGAAAACAATTTTCTTTTAGAATTTGCTTCAAG

>Chr05 SNP-58

AATTCGTTCATCTTCTCTTTTTTCCCAAAGACCGCTGGTAACCCATATCCATTTCACCAAATTAGCAATTTGATGAAGTACTCTCTTTCTCTCTCTAGATCACAACAACACGTCCTCCTCTCTCATTCTAGAAATTAGAAATCTAGAGAGGAAATTGATTTCTGCACTCTAAAACTTGATTTCTGCGCTCCTATTTGAGTGCATAAATCAACACAATTTCTGCACCCCTACTTTTACAAATTTTGAAGTGCAGAAATTATTTTTCCTTTAGAAATTGCATTC

>Chr05 SNP-59

CTTTTCCCTCTTCTCTTTTTTACCAAAGACCGCTGGTAACCCATATCCATTTCACCAAATTAGCAATTTGATGAAGTACTGTCTTTCTCTCTCTAGATCACAACAACACGTCCTCCTCTCTCATTCTAGAAATTAGAAATCTAAAAAGAAAATTGATTTCTGCACTCTAAAATTTAATTTCTGCACTCCTATTTAAGTGCAGAAATCAACGCCATTTCTGCACTCCTACTTTTATAAGTTTTGGAGTGCAGAAATTAAATTTCCTTTAGAAATTGCATTCAGGATGACCAAAA

>Chr05 SNP-60

GTCTTTTTACTCTTCTCTTTTTTCCCAAAGACCGCTGGTAACCCATATCCATTTCACCAAATTAGCAATTTGATGAAGTACTCTCTTTCTCTCTCTAGATCACAACAACATGTCCTCCTCTCTCGTTCTAGAAATTAGAAATCTGGAAAGAAAATTGATTTCTGCACTCTAAAACTTGATTTCTGCGCTCCTATTTGAGTGCATAAATCAACACAATTTCTGCACTCCTACTTTTACAAATTTTGAAGTGCAGAAATTATTTTTCCTTTAGAAATTGCATCCCAGAATGACCAAAA

>Chr05 SNP-61

TTCTTACCCTCTTCTCTTTTTTCCCAAAGACCGCTGGTAACCCATATCCATTTCACCAAATTAGCAATTTGATGAAGTACTCTCTTTCTCTCTCTAGATCACAACAACATGTCCTCCTCTCTGGTTCTAGAAATTAGAAATCTGGAAAGAAAATTGATTTCTGCACTCTAAAATTTGATTTCTGCACTCTTATTTGAGTATAGAAATCAACACCATTTCTGCACCCCTACTTTTACAAATTTTGAAGTGCAGAAAACAATTTTCTTTTAGAATTTGCTTCAAA

>Chr05 SNP-62

GTCTGTGCCTCTTCTCTTTTTTCCCAAAGACCGCTGGTAACCCATATCCATTTCACCAAATTAGCAATTTGATGAAGTACTCTCTTTCTCTCTCTAGATCACAACAACATGTCCTCCTCTCTCGTTCTAGAAATTAGAAATCTGGAAAGGAAATTGATTTCTGCACTCTAAAATTTGATTTCTGCACTCTTATTTGAGTGCAGAAATCAACACCATTTCTGCACCCCTACTTTTACAAATTTTGAAGTGCAGAAATTATTTTTCCTTTAGAAATTGCATCCAGGATGACCAAAG

>Chr05 SNP-63

GGCTTACTTCTCTTCTCTTTTTTCCCAAAGACCGCTGGTAACCCATATCCATTTCACCAAATTAGCAATTTGATGAAGTACTCTCTTTCTCTCTCTAGATCACAACAACATGTCCTCCTCTCTCGTTCTAGAAATTAGAAATCTGGAAAGAAAATTGATTTCTGCACTCTAAAATTTGATTTCTGCACTCTTATTTGAGTGCAGAAATCAACACAATTTCTGCACCCCTACTTTTACAAATTTTGAAGTGCAGAAATTATTTTTCCTTTAGAAATTGCATTCAG

>Chr05 SNP-64

GGCTGACGATCTTCTCTTTTTTCCCAAAGACCCCTGGTAACCCATATCCATTTCACCAAATTAGCAATTTGATGAAGTACTCTCTTTCTCTGTCTAGATCACAACAACACGTCCTCCTCTCTCATTCTAGAAATTAGAAATCTAGAAAGGAAATTGATTTCTGCACTCTAAAATTTGATTTCTGCACTCCTATTTGAGTGCAGATATCAACACCATTTCTGCACTCCTATTTTTACAAATTTTGAAGTGCAGAAATTATTTTTCCTTTAGAAATTGCATTC

>Chr05 SNP-65

GGATTATCCTCTTCTCTTTTTTCCCAAAGACCGCTGGTAACCCATATCCATTTCACCAAATTAGCAATTTGATGAAGTACTCTCTTTCTCTCTCTAGATCACAACAACATGTCCTCCTCTCTGGTTCTAGAAATTAGAAATCTGGAAAGAAAATTGATTTCTGCACTCTAAAATTTGATTTCTGCACTCTTATTTGAGTATAGAAATCAACACCATTTCTGCACCCCTACTTTTACAAATTTTGAAGTGCAGAAAACAATTTTCTTTTAGGATTTGCTTCC

>Chr05 SNP-66

GGACTTGCGATCTTCTCTTTTTTCCCAAAGACCGCTGGTAACCCATATCCATTTCACCAAATTAGCAATTTGATGAAGTACTCTCTTTCTCTCTCTAGATCACAACAACATGTCCTCCTCTCTCGTTCTAGAAATTAGAAATCTGGAAAGAAAATTGATTTCTGCACTCTAAAATTTGATTTCTGCACTCTTATTTGAGTATAGAAATCAACACCATTTCTGCACCCCTACTTTTACAAATTTTGAAGTGCAGAAAACAATTTTCTTTTAGGATTTGCTTCAA

>Chr05 SNP-67

GTCTTTACGCTCTTCTCTTTTTTCCCAAAGACCCCTGGTAACCCATATCCATTTCACCAAATTAGCAATTTGATGAAGTACTCTCTTTCTCTGTCTAGATCACAACAACACGTCCTCCTCTCTCATTCTAGAAATTAGAAATCTAGAAAGGAAATTGATTTCTGCACTCTAAAATTTGATTTCTGCACTCCTATTTGAGTGCAGATATCAACACCATTTCTGCACTCCTATTTTTACAAATTTTGAAGTGCAGAAATTATTTTTCCTTTAGAAATTGCATCCAG

>Chr05 SNP-68

GACTTACGATCTTCTCTTTTTTCCCAAAGACCGCTGGTAACCCATATCCATTTCACCAAATTAGCAATTTGATGAAGTACTCTCTTTCTCTCTCTAGATCACAACAACACGTCCTCCTCTCTGGTTCTAGAAATTAGAAATCTGGAAAGAAAATTGATTTCTGCACTCTAAAATTTGATTTCTGCACTCCTATTTGAGTATAGAAATCAACGCCATTTCTGCACTCCTACTTTTACAAATTTTGGAGTGCAGAAAACAATTTCCTTTAGAAATTGGATTCCAGAAAGGACCAAAAA

>Chr05 SNP-69

CGCTTACGCTCTTCTCTTTTTTCCCAAAGACCGCTGGTAACCCATATCCATTTCACCAAATTAGCAATTTGATGAAGTACTCTCTTTCTCTCTCTAGATCACAACAACATGTCCTCCTCTCTCGTTCTAGAAATTAGAAATCTGGAGAGGAAATTGATTTCTGCACTCTAAAACTTGATTTCTGCGCTCCTATTTGAGTGCATAAATCAACACAATTTCTGCACTCCTACTTTTACAAATTTTGAAGTGCAGAAATTATTTTTCCTTTAGAAATTGCATTCAG

>Chr05 SNP-70

TTCTTACGCTCTTCTCTTTTTTCCCAAAGACCCCTGGTAACCCATATCCATTTCACCAAATTAGCAATTTGATGAAGTACTCTCTTTCTCTGTCTAGATCACAACAACACGTCCTCCTCTCTCATTCTAGAAATTAGAAATCTAGAAAGGAAATTGATTTCTGCACTCTAAAATTTGATTTCTGCACTCCTATTTGAGTGCAGATATCAACACCATTTCTGCACTCCTATTTTTACAAATTTTGAAGTGCAGAAATTATTTTTCCTTTAGAAATTGCATTCAG

>Chr05 SNP-71

GTCTGACGACTCTTCTCTTTTTTCCCAAAGACCGCTGGTAACCCATATCCATTTCACCAAATTAGCAATTTGATGAAGTACTCTCTTTCTCTCTCTAGATCACAACAACACGTCCTCCTCTCTCATTCTAGAAATTAGAAATCTAGAGAGGAAATTGATTTCTGCACTCTAAAACTTGATTTCTGCGCTCCTATTTGAGTGCATAAATCAACACAATTTCTGCACTCCTACTTTTACAAATTTTGAAGTGCAGAAATTATTTTTCCTTTAGAAATTGCATTC

>Chr05 SNP-72

GTTCTTGCGTCTCTTCTCTTTTTTCCCAAAGACCGCTGGTAACCCATATCCATTTCACCAAATTAGCAATTTGATGAAGTACTCTCTTTCTCTCTCTAGATCACAACAACATGTCCTCCTCTCTCGTTCTAGAAATTAGAAATCTGGAAAGAAAATTGATTTCTGCACTCTAAAATTTGATTTCTGCACTCTTATTTGAGTGCAGAAATCAACACAATTTCTGCACCCCTACTTTTACAAATTTTGAAGTGCAGAAATTATTTTTCCTTTAGAAATTGCATTCAGGAATGACCAAAGG

>Chr05 SNP-73

ACTACTGACGATCTTCTCTTTTTTCCCAAAGACCCCTGGTAACCCATATCCATTTCACCAAATTAGCAATTTGATGAAGTACTCTCTTTCTCTCTCTAGATCACAACAACACGTCCTCCTCTCTGATTCTAGAAATTAGAAATCTGGAAAGGAAATTGATTTCTGCACTCTAAAATTTGATTTCTGCACTCCTATTTGAGTGTAGAAATCAACACCATTTCTGCACTCCTACTTTTACAAATTTTGAAGTGCAGAAAACAATTTTCTTTTAGAATTTCCATAAG

>Chr05 SNP-74

GACTTACGCTCTTCTCTTTTTTCCCAAAGACCCCTGGTAACCCATATCCATTTCACCAAATTAGCAATTTGATGAAGTACTCTCTTTCTCTGTCTAGATCACAACAACACGTCCTCCTCTCTCATTCTAGAAATTAGAAATCTAGAAAGGAAATTGATTTCTGCACTCTAAAATTTGATTTCTGCACTCCTATTTGAGTGCAGATATCAACACCATTTCTGCACTCCTATTTTTACAAATTTTGAAGTGCAGAAATTATTTTTCCTTTAGAAATTGCATTC

>Chr05 SNP-75

GGATTTACGCTCTTCTCTTTTTTCCCAAAGACCCCTGGTAACCCATATCCATTTCACCAAATTAGCAATTTGATGAAGTACTCTCTTTCTCTGTCTAGATCACAACAACACGTCCTCCTCTCTCATTCTAGAAATTAGAAATCTAGAAAGGAAATTGATTTCTGCACTCTAAAATTTGATTTCTGCACTCCTATTTGAGTGCAGATATCAACACCATTTCTGCACTCCTATTTTTACAAATTTTGAAGTGCAGAAATTATTTTTCCTTTAGAAATTGCATTCAGG

>Chr05 SNP-76

GGCTTACGCTCTTCTCTTTTTTCCCAAAGACCGCTGGTAACCCATATCCATTTCACCAAATTAGCAATTTGATGAAGTACTCTCTTTCTCTCTCTAGATCACAACAACATGTCCTCCTCTCTCGTTCTAGAAATTAGAAATCTGGAAAGAAAATTGATTTCTGCACTCTAAAATTTGATTTCTGCACTCTTATTTGAGTGCAGAAATCAACACAATTTCTGCACCCCTACTTTTACAAATTTTGAAGTGCAGAAATTATTTTTCCTTTAGAAATTGCATTCAGGAATGACCAAAG

>Chr05 SNP-77

TTGTTTACCCTCTTCTCTTTTTTCCCAAAGACCGCTGGTAACCCATATCCATTTCACCAAATTAGCAATTTGATGAAGTACTCTCTTTCTCTCTCTAGATCACAACAACATGTCCTCCTCTCTCGTTCTAGAAATTAGAAATCTGGAAAGAAAATTGATTTCTGCACTCTAAAATTTGATTTCTGCACTCTTATTTGAGTGCAGAAATCAACACAATTTCTGCACCCCTACTTTTACAAATTTTGAAGTGCAGAAATTATTTTTCCTTTAGAAATTGCATTCAGGAATGACCAAAG

>Chr05 SNP-78

GGGCCAGCTCAGCTTCTCTCTTTTTTTTCCCAAAGACCGCTGGTAACCCATATCCATTTCACCAAATTAGCAATTTGATGAAGTACTCTCTTTCTCTGTCTAGATCACAACAACATGTCCTCCTCTCTCGTTCTAGAAATTAGAAATCTGGAAAGAAAATTGATTTCTGCACTCTAAAATTTGATTTCTGCACTCTTATTTGAGTGCAGAAATCAACACCATTTCTGCACCCCTATTTTTACAAATTTTGAAGTGCAGAAATTATTTTTCCTTTAGAAATTGCATTCCAGAATTT

>Chr05 SNP-79

GTCTGTCCCTCTTCTCTTTTTTCCCAAAGACCCCTGGTAACCCATATCCATTTCACCAAATTAGCAATTTGATGAAGTACTCTCTTTCTCTGTCTAGATCACAACAACACGTCCTCCTCTCTCATTCTAGAAATTAGAAATCTAGAAAGGAAATTGATTTCTGCACTCTAAAATTTGATTTCTGCACTCCTATTTGAGTGCAGATATCAACACCATTTCTGCACTCCTATTTTTACAAATTTTGAAGTGCAGAAATTATTTTTCCTTTAGAAATTGCATTCAGAATGACCAAACA

>Chr05 SNP-80

CTTTTTGACTCTTCTCTTTTTTCCCAAAGACCGCTGGTAACCCATATCCATTTCACCAAATTAGCAATTTGATGAAGTACTGTCTTTCTCTCTCTAGATCACAACAACACGTCCTCCTCTCTCATTCTAGAAATTAGAAATCTAAAAAGAAAATTGATTTCTGCACTCTAAAATTTAATTTCTGCACTCCTATTTAAGTGCAGAAATCAACGCCATTTCTGCACTCCTACTTTTATAAGTTTTGGAGTGCAGAAATTAAATTTCCTTTAGAAATTGCATTCAGGAATGACCAAAS

>Chr05 SNP-81

GGCCCTACGATCTTCTCTTTTTTCCCAAAGACCCCTGGTAACCCATATCCATTTCACCAAATTAGCAATTTGATGAAGTACTCTCTTTCTCTGTCTAGATCACAACAACACGTCCTCCTCTCTCATTCTAGAAATTAGAAATCTAGAAAGGAAATTGATTTCTGCACTCTAAAATTTGATTTCTGCACTCCTATTTGAGTGCAGATATCAACACCATTTCTGCACTCCTATTTTTACAAATTTTGAAGTGCAGAAATTATTTTTCCTTTAGAAATTGCATTCAGG

>Chr05 SNP-82

GGCAGACGATCTTCTCTTTTTTCCCAAAGACCGCTGGTAACCCATATCCATTTCACCAAATTAGCAATTTGATGAAGTACTCTCTTTCTCTCTCTAGATCACAACAACACGTCCTCCTCTCTCATTCTAGAAATTAGAAATCTGGAGAGGAAATTGATTTCTGCACTCTAAAACTTGATTTCTGCGCTCCTATTTGAGTATATAAATCAACACCATTTCTGCACTCCTACTTTTACAAATTTTGAAGTGCAGAAAACAATTTCCTTTTAAAATTTGCATAA

>Chr05 SNP-83

GGTCTTACGATCTTCTCTTTTTTCCCAAAGACCGCTGGTAACCCATATCCATTTCACCAAATTAGCAATTTGATGAAGTACTCTCTTTCTCTCTCTAGATCACAACAACACGTCCTCCTCTCTCATTCTAGAAATTAGAAATCTGGAGAGGAAATTGATTTCTGCACTCTAAAATTTGATTTCTGCGCTCCTATTTGAGTATATAAATCAACACCATTTCTGCACTCCTACTTTTACAAATTTTGAAGTGCAGAAATTATTTTTCCTTAAAAAATGGAATCA

>Chr05 SNP-84

CGCTGTACGCTCTTCTCTTTTTTCCCAAAGACCGCTGGTAACCCATATCCATTTCACCAAATTAGCAATTTGATGAAGTACTCTCTTTCTCTCTCTAGATCACAACAACATGTCCTCCTCTCTCGTTCTAGAAATTAGAAATCTGGAAAGAAAATTGATTTCTGCACTCTAAAATTTGATTTCTGCACTCTTATTTGAGTGCAGAAATCAACACAATTTCTGCACCCCTACTTTTACAAATTTTGAAGTGCAGAAATTATTTTTCCTTTAGAAATTGCATTCAGG

>Chr05 SNP-85

GGGGTTTTCCTCTTCTCTTTTTTCCCAAAGACCGCTGGTAACCCATATCCATTTCACCAAATTAGCAATTTGATGAAGTACTGTCTTTCTCTCTCTAGATCACAACAACACGTCCTCCTCTCTCATTCTAGAAATTAGAAATCTAAAAAGAAAATTGATTTCTGCACTCTAAAATTTAATTTCTGCACTCCTATTTAAGTGCAGAAATCAACGCCATTTCTGCACTCCTACTTTTATAAGTTTTGGAGTGCAGAAATTAAATTTCCTTTAGAAATTGCATTCCAGATGACCAAAAC

>Chr05 SNP-86

GACTTACGATCTTCTCTTTTTTCCCAAAGACCCCTGGTAACCCATATCCATTTCACCAAATTAGCAATTTGATGAAGTACTCTCTTTCTCTGTCTAGATCACAACAACACGTCCTCCTCTCTCATTCTAGAAATTAGAAATCTAGAAAGGAAATTGATTTCTGCACTCTAAAATTTGATTTCTGCACTCCTATTTGAGTGCAGATATCAACACCATTTCTGCACTCCTATTTTTACAAATTTTGAAGTGCAGAAATTATTTTTCCTTTAGAAATTGCATTCAGGAATGGACCAAA

>Chr05 SNP-87

GGCGTACTTACTCTTCCTCTTTTTTCCCAAAGACCGCTGGTAACCCATATCCATTTCACCAAATTAGCAATTTGATGAAGTACTCTCTTTCTCTCTCTAGATCACAACAACATGTCCTCCTCTCTCGTTCTAGAAATTAGAAATCTGGAAAGGAAATTGATTTCTGCACTCTAAAATTTGATTTCTGCACTCTTATTTGAGTGCAGAAATCAACACCATTTCTGCACCCCTATTTTTACAAATTTTGAAGTGCAGAAATTATTTTTCCTTTAGAAATTGCATCA

>Chr05 SNP-88

GTACTTACTTATCTTCATCTTTTTTCCCAAAGACCGCTGGTAACCCATATCCATTTCACCAAATTAGCAATTTGATGAAGTACTCTCTTTCTCTCTCTAGATCACAACAACACGTCCTCCTCTCTCATTCTAGAAATTAGAAATCTAAAAAGAAAATTGATTTCTGCACTCTAAAATTTGATTTCTGCACTCCTATTTGAGTGCAGAAATCAACGCCATTTCTGCACTCCTACTTTTATAAATTTTGGAGTGCAGAAATTATATTTCTTTTAGAAATTGCATCAGAA

>Chr05 SNP-89

GACGCTATCTTCTCTTTTTTCCCAAAGACCCCTGGTAACCCATATCCATTTCACCAAATTAGCAATTTGATGAAGTACTCTCTTTCTCTGTCTAGATCACAACAACACGTCCTCCTCTCTCATTCTAGAAATTAGAAATCTAGAAAGGAAATTGATTTCTGCACTCTAAAATTTGATTTCTGCACTCCTATTTGAGTGCAGATATCAACACCATTTCTGCACTCCTATTTTTACAAATTTTGAAGTGCAGAAATTATTTTTCCTTTAGAAATTGCATC

>Chr05 SNP-90

ATTCTTGGCTCTTCTCTTTTTTCCCAAAGACCGCTGGTAACCCATATCCATTTCACCAAATTAGCAATTTGATGAAGTACTCTCTTTCTCTCTCTAGATCACAACAACACGTCCTCCTCTCTCATTCTAGAAATTAGAAATCTAAAAAGAAAATTGATTTCTGCACTCTAAAATTTAATTTCTGTACTCCTATTTAAGTGCAGAAATCAACGCCATTTCTGCACTCCTACTTTTATAAATTTTGGAGTGCAGAAATTAAATTTCTTTTAGAAATTGCATTCAGGATGACCAAAGGG

>Chr05 SNP-91

GGCGTACGATCTTCTCTTTTTTCCCAAAGACCGCTGGTAACCCATATCCATTTCACCAAATTAGCAATTTGATGAAGTACTCTCTTTCTCTCTCTAGATCACAACAACACGTCCTCCTCTCTCATTCTAGAAATTAGAAATCTAAAAAGAAAATTGATTTCTGCACTCTAAAATTTAATTTCTGCACTCCTATTTAAGTGCAGAAATCAACGCCATTTCTGCACTCCTACTTTTATAAGTTTTGGAGTGCAGAAATTAAATTTCCTTTAGAAATTGCTTCA

>Chr05 SNP-92

GGTTTTCCTCTTCTCTTTTTTCCCAAAGACCGCTGGTAACCCATATCCATTTCACCAAATTAGCAATTTGATGAAGTACTGTCTTTCTCTCTCTAGATCACAACAACACGTCCTCCTCTCTCATTCTAGAAATTAGAAATCTAAAAAGAAAATTGATTTCTGCACTCTAAAATTTAATTTCTGCACTCCTATTTAAGTGCAGAAATCAACGCCATTTCTGCACTCCTACTTTTATAAGTTTTGGAGTGCAGAAATTAAATTTCCTTTAGAAATTGCATTCCAGATGACCAAAAC

>Chr05 SNP-93

ATTCTTGGCTCTTCTCTTTTTTCCCAAAGACCGCTGGTAACCCATATCCATTTCACCAAATTAGCAATTTGATGAAGTACTCTCTTTCTCTCTCTAGATCACAACAACACGTCCTCCTCTCTCATTCTAGAAATTAGAAATCTAAAAAGAAAATTGATTTCTGCACTCTAAAATTTAATTTCTGTACTCCTATTTAAGTGCAGAAATCAACGCCATTTCTGCACTCCTACTTTTATAAATTTTGGAGTGCAGAAATTAAATTTCTTTTAGAAATTGCATTCAGGATGACCAAAGGG

>Chr05 SNP-94

GTATTACTATCTTCTCTTTTTTCCCAAAGACCGCTGGTAACCCATATCCATTTCACCAAATTAGCAATTTGATGAAGTACTCTCTTTCTCTCTCTAGATCACAACAACATGTCCTCCTCTCTCGTTCTAGAAATTAGAAATCTGGAAAGAAAATTGATTTCTGCACTCTAAAATTTGATTTCTGCACTCTTATTTGAGTGCAGAAATCAACACAATTTCTGCACCCCTACTTTTACAAATTTTGAAGTGCAGAAATTATTTTTCCTTTAGAAATTGCATTC

>Chr05 SNP-95

AACTTACTGCGATCTTCTCTTTTTTCCCAAAGACCCCTGGTAACCCATATCCATTTCACCAAATTAGCAATTTGATGAAGTACTCTCTTTCTCTGTCTAGATCACAACAACACGTCCTCCTCTCTCATTCTAGAAATTAGAAATCTAGAAAGGAAATTGATTTCTGCACTCTAAAATTTGATTTCTGCACTCCTATTTGAGTGCAGATATCAACACCATTTCTGCACTCCTATTTTTACAAATTTTGAAGTGCAGAAATTATTTTTCCTTTAGAAATTGCATTCAG

>Chr05 SNP-96

GGACTTACTATCTTCTCTTTTTTCCCAAAGACCCCTGGTAACCCATATCCATTTCACCAAATTAGCAATTTGATGAAGTACTCTCTTTCTCTGTCTAGATCACAACAACACGTCCTCCTCTCTCATTCTAGAAATTAGAAATCTAGAAAGGAAATTGATTTCTGCACTCTAAAATTTGATTTCTGCACTCCTATTTGAGTGCAGATATCAACACCATTTCTGCACTCCTATTTTTACAAATTTTGAAGTGCAGAAATTATTTTTCCTTTAGAAATTGCATTCAG

>Chr05 SNP-97

ACTACTCTTCTCTTTTTTCCCAAAGACCGCTGGTAACCCATATCCATTTCACCAAATTAGCAATTTGATGAAGTACTCTCTTTCTCTCTCTAGATCACAACAACACGTCCTCCTCTCTCATTCTAGAAATTAGAAATCTAGAGAGGAAATTGATTTCTGCACTCTAAAACTTGATTTCTGCACTCCTATTTGAGTGCATAAATCAACACCATTTCTGCACTCCTACTTTTACAAATTTTGAAGTGCAGAAATTATTTTTCCTTTAGAAATTGCATC

>Chr05 SNP-98

GCTACTCTTCATCTTTTTTCCCAAAGACCGCTGGTAACCCATATCCATTTCACCAAATTAGCAATTTGATGAAGTACTCTCTTTCTCTCTCTAGATCACAACAACACGTCCTCCTCTCTCATTCTAGAAATTAGAAATCTGGAGAGGAAATTGATTTCTGCACTCTAAAACTTGATTTCTGCGCTCCTATTTGAGTATATAAATCAACACCATTTCTGCACTCCTACTTTTACAAATTTTGAAGTGCAGAAATTATTTTTCCTTAAAAAATGGCATCAG

>Chr05 SNP-99

TACTACTCTTCTCTTTTTTCCCAAAGACCGCTGGTAACCCATATCCATTTCACCAAATTAGCAATTTGATGAAGTACTCTCTTTCTCTCTCTAGATCACAACAACATGTCCTCCTCTCTCGTTCTAGAAATTAGAAATCTGGAAAGGAAATTGATTTCTGCACTCTAAAATTTGATTTCTGCACTCTTATTTGAGTGCAGAAATCAACACCATTTCTGCACCCCTACTTTTACAAATTTTGAAGTGCAGAAATTATTTTTCCTTTAGAAATTGCAT

>Chr05 SNP-100

GCACTACTCTTCTCTTTTTTCCCAAAGACCGCTGGTAACCCATATCCATTTCACCAAATTAGCAATTTGATGAAGTACTCTCTTTCTCTGTCTAGATCACAACAACACGTCCTCCTCTCTGATTCTAGAAATTAGAAATCTGGAAAGGAAATTGATTTCTGCACTCTAAAATTTGATTTCTGCACTCCTATTTGAGTGTAGAAATCAACACCATTTCTGCACTCCTACTTTTACAAATTTTGAAGTGCAGAAAACAATTTCCTTTTAGAATTTGCTTCAA

>Chr05 SNP-101

GAGGGACGACTCTTCCTCTTTTTTCCCAAAGACCGCTGGTAACCCATATCCATTTCACCAAATTAGCAATTTGATGAAGTACTCTCTTTCTCTCTCTAGATCACAACAACATGTCCTCCTCTCTCGTTCTAGAAATTAGAAATCTGGAAAGAAAATTGATTTCTGCACTCTAAAATTTGATTTCTGCACTCTTATTTGAGTGCAGAAATCAACACAATTTCTGCACCCCTACTTTTACAAATTTTGAAGTGCAGAAATTATTTTTCCTTTAGAAATTGCATCAG

>Chr05 SNP-102

GGTTGTGTCTCTTCTCCTTTTTCCCAAAGACCCCTGGTAACCCATATCCATTTCACCAAATTAGCAATTTGATGTACTCTCTTTCTCTCTCTAGATCACAACAATAAGTCCTCCTCTCTCATTCTAGAAATTAGAAATCTAGAAAGGAAATTGATTTCTGCACTCTAAAATTTGATTTTTGCACTCCTATTTGAGTGCAGAAATCAACATCATTTCTGCACTCTTATTTTTACAAATTTTGGAGTACAGAAAACAATTTCCTTTAGAAATTGCATCCAGATGACCAAACCAAA

>Chr05 SNP-103

GGCTGCTACTCTTCTCTTTTTTCCCAAAGACCCCTGGTAACCCATATCCATTTCACCAAATTAGCAATTTGATGAAGTACTCTCTTTCTCTCTCTAGATCACAACAATAAGTCCTCCTCTCTCATTCTAGAAATTAGAAATCTAGAAAGGAAATTAATTTCTGCACTCTAAAATTTGATTTTTGCACTCCTATTTGAGTGCAGAAATCAACATCATTTCTGCACTCTTACTTTTACAAATTTTGGAGTGCAGAAAACAATTTCCTTTAGAAATTGCATTCAGGAATGACCAAAAGG

>Chr05 SNP-104

>Chr05 SNP-105

GGCTGCGTACTCTTCTCTTTTTTCCCAAGACCGCTGGTAACCCATATCCATTTCACCAAATTAGCAATTTGATGAAGTACTCTCTTTCTCTCTCTAGATCACAACAACACGTCCTCCTCTCTGGTTCTAGAAATTAGAAATCTAGAGAGGAAATTGATTTCTGCACTCTAAAACTTGATTTCTGCGCTCCTATTTGAGTGTATAAATCAACACCATTTCTGCACTCCTACTTTTACAAATTTTGAAGTGCAGAAATTATTTTTCCTTTAAAAATGGCTTTCCAGAAGGACCAAAA

>Chr05 SNP-106

GGTGGCGGCTTCTCTTCTCTTTTTTCCCAAGACCGCTGGTAACCCATATCCATTTCACCAAATTAGCAATTTGATGAAGTACTCTCTTTCTCTCTCTAGATCACAACAACATGTCCTCCTCTCTGGTTCTAGAAATTAGAAATCTGGAAAGAAAATTGATTTCTGCACTCTAAAATTTGATTTCTGCACTCTTATTTGAGTGTAGAAATCAACACCATTTCTGCACCCCTACTTTTACAAATTTTGAAGTGCAGAAATTATTTTTCTTTAAAAAATTGCTTTCCAAAGGACCAAAGG

>Chr05 SNP-107

GGCGGCTATCTTCTCTTTTTTCCCAAAGACCCCTGGTAACCCATATCCATTTCACCAAATTAGCAATTTGATGAAGTACTCTCTTTCTCTCTCTAGATCACAACAACACGTCCTCCTCTCTCATTCTAGAAATTAGAAATCTAAAAAGAAAATTGATTTCTGCACTCTAAAATTTGATTTCTGCACTCCTATTTGAGTGCAGAAATCAACGCCATTTCTGCACTCCTACTTTTATAAATTTTGGAGTGCAGAAATTATATTTCTTTTAGAAATTGCATTC

>Chr05 SNP-108

GTCTTTTTTCTCTTCTCTTTTTTCCCAAAGACCCCTGGTAACCCATATCCATTTCACCAAATTAGCAATTTGATGAAGTACTCTCTTTCTCTGTCTAGATCACAACAACACGTCCTCCTCTCTCATTCTAGAAATTAGAAATCTAGAAAGGAAATTGATTTCTGCACTCTAAAATTTGATTTCTGCACTCCTATTTGAGTGCAGATATCAACACCATTTCTGCACTCCTATTTTTACAAATTTTGAAGTGCAGAAATTATTTTTCCTTTAGAAATTGCATTCCAGATGACCAAAAA

>Chr05 SNP-109

GGTTGGTATCTTCTCTTTTTTCCCAAAGACCGCTGGTAACCCATATCCATTTCACCAAATTAGCAATTTGATGAAGTACTCTCTTTCTCTCTCTAGATCACAACAACATGTCCTCCTCTCTGGTTCTAGAAATTAGAAATCTGGAAAGAAAATTGATTTCTGCACTCTAAAATTTGATTTCTGCACTCTTATTTGAGTGTAGAAATCAACACAATTTCTGCACCCCTACTTTTACAAATTTTGAAGTGCAGAAATTATTTTTCCTTTAAAAATTGCTTCCAGAAA

>Chr05 SNP-110

GGCTCGTACTCTTCTCTTTTTTCCCAAAGACCCCTGGTAACCCATATCCATTTCACCAAATTAGCAATTTGATGAAGTACTCTCTTTCTCTGTCTAGATCACAACAACACGTCCTCCTCTCTCATTCTAGAAATTAGAAATCTAGAAAGGAAATTGATTTCTGCACTCTAAAATTTGATTTCTGCACTCCTATTTGAGTGCAGATATCAACACCATTTCTGCACTCCTATTTTTACAAATTTTGAAGTGCAGAAATTATTTTTCCTTTAGAAATTGCATTCAG

>Chr05 SNP-111

GGAGCACCTCTCTTCTCTTTTTTCCCAAAAGACCGCTGGTAACCCATATCCATTTCACCAAATTAGCAATTTGATGAAGTACTCTCTTTCTCTGTCTAGATCACAACAACATGTCCTCCTCTCTCGTTCTAGAAATTAGAAATCTGGAAAGAAAATTGATTTCTGCACTCTAAAATTTGATTTCTGCACTCTTATTTGAGTGCAGATATCAACACCATTTCTGCACTCCTATTTTTACAAATTTTGAAGTGCAGAAATTATTTTTCCTTTAGAAATTGCATTCAGGATGACCC

>Chr05 SNP-112

GGTTGCGTCTCTTCTCTTTTTTCCCAAAGACCGCTGGTAACCCATATCCATTTCACCAAATTAGCAATTTGATGAAGTACTCTCTTTCTCTGTCTAGATCACAACAACACGTCCTCCTCTCTCATTCTAGAAATTAGAAATCTAGAAAGGAAATTGATTTCTGCACTCTAAAATTTGATTTCTGCACTCCTATTTGAGTGCAGATATCAACGCCATTTCTGCACTCCTATTTTTACAAATTTTGAAGTGCAGAAATTATTTTTCCTTTAGAAATTGCATTCCAGATGACCAAA

>Chr05 SNP-113

GGGGGGTGCTTCTCTTCTCTTTTTTCCCAAAGACCGCTGGTAACCCATATCCATTTCACCAAATTAGCAATTTGATGAAGTACTCTCTTTCTCTCTCTAGATCACAACAACATGTCCTCCTCTCTCGTTCTAGAAATTAGAAATCTGGAAAGGAAATTGATTTCTGCACTCTAAAATTTGATTTCTGCACTCTTATTTGAGTGCAGAAATCAACACCATTTCTGCACCCCTATTTTTACAAATTTTGAAGTGCAGAAATTATTTTTCCTTTAGAAATTGCATTCCAGAATGACCAAAAC

>Chr05 SNP-114

GGTTCGTCTCTTCTCTTTTTTCCCAAAGACCCCTGGTAACCCATATCCATTTCACCAAATTAGCAATTTGATGAAGTACTCTCTTTCTCTGTCTAGATCACAACAACACGTCCTCCTCTCTCATTCTAGAAATTAGAAATCTAGAAAGGAAATTGATTTCTGCACTCTAAAATTTGATTTCTGCACTCCTATTTGAGTGCAGATATCAACACCATTTCTGCACTCCTATTTTTACAAATTTTGAAGTGCAGAAATTATTTTTCCTTTAGAAATTGCATTCCAGAATGACCAAAGG

>Chr05 SNP-115

TGCTTACGACTCTTCTCTTTTTTCCCAAAGACCGCTGGTAACCCATATCCATTTCACCAAATTAGCAATTTGATGAAGTACTCTCTTTCTCTCTCTAGATCACAACAACACGTCCTCCTCTCTCATTCTAGAAATTAGAAATCTAGAGAGGAAATTGATTTCTGCACTCTAAAACTTGATTTCTGCGCTCCTATTTGAGTGCATAAATCAACACAATTTCTGCACTCCTACTTTTACAAATTTTGAAGTGCAGAAATTATTTTTCCTTTAGAAATTGCATTCA

>Chr05 SNP-116

GGCTCCTACTCTTCATCTTTTTTCCCAAAGACCGCTGGTAACCCATATCCATTTCACCAAATTAGCAATTTGATGAAGTACTCTCTTTCTCTCTCTAGATCACAACAACACGTCCTCCTCTCTGGTTCTAGAAATTAGAAATCTGGAAAGAAAATTGATTTCTGCACTCTAAAATTTGATTTCTGCACTCCTATTTGAGTGTAGAAATCAACACCATTTCTGCACTCCTACTTTTACAAATTTTGGAGTGCAGAAAACAATTTCCTTTAGAAATTGGATTCAGGA

>Chr05 SNP-117

GGTTCGACTCTTCTCTTTTTTCCCAAAGACCCCTGGTAACCCATATCCATTTCACCAAATTAGCAATTTGATGAAGTACTCTCTTTCTCTGTCTAGATCACAACAACACGTCCTCCTCTCTCATTCTAGAAATTAGAAATCTAGAAAGGAAATTGATTTCTGCACTCTAAAATTTGATTTCTGCACTCCTATTTGAGTGCAGATATCAACACCATTTCTGCACTCCTATTTTTACAAATTTTGAAGTGCAGAAATTATTTTTCCTTTAGAAATTGCATTCAG

>Chr05 SNP-118

NGTGCTAGACTCTCTCTCTTTTTTCCCAAAAGACCGCTGGTAACCCATATCCATTTCACCAAATTAGCAATTTGATGAAGTACTCTCTTTCTCTGTCTAGATCACAACAACATGTCCTCCTCTCTCGTTCTAGAAATTAGAAATCTGGAAAGAAAATTGATTTCTGCACTCTAAAATTTGATTTCTGCACTCCTATTTGAGTGCAGATATCAACACCATTTCTGCACTCCTATTTTTACAAATTTTGAAGTGCAGAAATTATTTTTCCTTTAGAAATTGCATTCCAGAATGACC

>Chr05 SNP-119

GAGGGACGACTCTTCCTCTTTTTTCCCAAAGACCGCTGGTAACCCATATCCATTTCACCAAATTAGCAATTTGATGAAGTACTCTCTTTCTCTCTCTAGATCACAACAACATGTCCTCCTCTCTCGTTCTAGAAATTAGAAATCTGGAAAGAAAATTGATTTCTGCACTCTAAAATTTGATTTCTGCACTCTTATTTGAGTGCAGAAATCAACACAATTTCTGCACCCCTACTTTTACAAATTTTGAAGTGCAGAAATTATTTTTCCTTTAGAAATTGCATCAG

>Chr05 SNP-120

GCTGTTCTCTTCTCTTTTTTCCCAAAGACCGCTGGTAACCCATATCCATTTCACCAAATTAGCAATTTGATGAAGTACTCTCTTTCTCTGTCTAGATCACAACAACACGTCCTCCTCTCTGGTTCTAGAAATTAGAAATCTGGAAAGGAAATTGATTTCTGCACTCTAAAATTTGATTTCTGCACTCCTATTTGAGTGTAGATATCAACACCATTTCTGCACTCCTATTTTTACAAATTTTGAAGTGCAGAAAATATTTTTCCTTAAAAAATGGTTCCAG

>Chr05 SNP-121

GGCGCCTCTCTTCTCTTTTTTCCCAAAGACCCCTGGTAACCCATATCCATTTCACCAAATTAGCAATTTGATGAAGTACTCTCTTTCTCTGTCTAGATCACAACAACACGTCCTCCTCTCTCATTCTAGAAATTAGAAATCTAGAAAGGAAATTGATTTCTGCACTCTAAAATTTGATTTCTGCACTCCTATTTGAGTGCAGATATCAACGCCATTTCTGCACTCCTATTTTTACAAATTTTGAAGTGCAGAAATTATTTTTCCTTTAGAAATTGCATTCA

>Chr05 SNP-122

GTTTACGACTCTTCTCTTTTTTCCCAAAGACCGCTGGTAACCCATATCCATTTCACCAAATTAGCAATTTGATGAAGTACTCTCTTTCTCTCTCTAGATCACAACAACACGTCCTCCTCTCTGGTTCTAGAAATTAGAAATCTGGAGAGGAAATTGATTTCTGCACTCTAAAACTTGATTTCTGCGCTCCTATTTGAGTGCATAAATCAACACCATTTCTGCACTCCTACTTTTACAAATTTTGAAGTGCAGAAATTATTTTTCCTTTAAAAATGGCTCCAGA

>Chr05 SNP-123

GGTTTTCCTCTTCTCTTTTTTCCCAAAGACCGCTGGTAACCCATATCCATTTCACCAAATTAGCAATTTGATGAAGTACTGTCTTTCTCTCTCTAGATCACAACAACACGTCCTCCTCTCTCATTCTAGAAATTAGAAATCTAAAAAGAAAATTGATTTCTGCACTCTAAAATTTAATTTCTGCACTCCTATTTAAGTGCAGAAATCAACGCCATTTCTGCACTCCTACTTTTATAAGTTTTGGAGTGCAGAAATTAAATTTCCTTTAGAAATTGCATTCCAGATGACCAAAAC

>Chr05 SNP-124

GCTCTTCTCTTCTCCTTTTTCCCAAAGACCCCTGGTAACCCATATCCATTTCACCAAATTAGCAATTTGATGTACTCTCTTTCTCTCTCTAGATCACAACAATAAGTCCTCCTCTCTCATTCTAGAAATTAGAAATCTAGAAAGGAAATTGATTTCTGCACTCTAAAATTTGATTTTTGCACTCCTATTTGAGTGCAGAAATCAACATCATTTCTGCACTCTTATTTTTACAAATTTTGGAGTACAGAAAACAATTTCCTTTAGAAATTGCATTCAATATGACCAAACC

>Chr05 SNP-125

CGTACTACTCTTCTCTTTTTTCCCAAAGACCCCTGGTAACCCATATCCATTTCACCAAATTAGCAATTTGATGAAGTACTCTCTTTCTCTGTCTAGATCACAACAACACGTCCTCCTCTCTCATTCTAGAAATTAGAAATCTAGAAAGGAAATTGATTTCTGCACTCTAAAATTTGATTTCTGCACTCCTATTTGAGTGCAGATATCAACGCCATTTCTGCACTCCTATTTTTACAAATTTTGAAGTGCAGAAATTATTTTTCCTTTAGAAATTGCATTCAGG

>Chr05 SNP-126

GTACCTTACCCTCTTCTCTTTTTTCCCAAAGACCGCTGGTAACCCATATCCATTTCACCAAATTAGCAATTTGATGAAGTACTCTCTTTCTCTCTCTAGATCACAACAACACGTCCTCCTCTCTCATTCTAGAAATTAGAAATCTAGAGAGGAAATTGATTTCTGCACTCTAAAACTTGATTTCTGCGCTCCTATTTGAGTGCATAAATCAACACAATTTCTGCACCCCTACTTTTACAAATTTTGAAGTGCAGAAATTATTTTTCCTTTAGAAATTGCAT

>Chr05 SNP-127

CTTTTCCCTCTTCTCTTTTTTACCAAAGACCGCTGGTAACCCATATCCATTTCACCAAATTAGCAATTTGATGAAGTACTGTCTTTCTCTCTCTAGATCACAACAACACGTCCTCCTCTCTCATTCTAGAAATTAGAAATCTAAAAAGAAAATTGATTTCTGCACTCTAAAATTTAATTTCTGCACTCCTATTTAAGTGCAGAAATCAACGCCATTTCTGCACTCCTACTTTTATAAGTTTTGGAGTGCAGAAATTAAATTTCCTTTAGAAATTGCATTCAGGATGACCAAAA

>Chr05 SNP-128

GGCTGCTATCTTCATCTTTTTTCCCAAAGACCCCTGGTAACCCATATCCATTTCACCAAATTAGCAATTTGATGAAGTACTCTCTTTCTCTGTCTAGATCACAACAACACGTCCTCCTCTCTCATTCTAGAAATTAGAAATCTAGAAAGGAAATTGATTTCTGCACTCTAAAATTTGATTTCTGCACTCCTATTTGAGTGCAGATATCAACACCATTTCTGCACTCCTATTTTTACAAATTTTGAAGTGCAGAAATTATTTTTCCTTTAGAAATTGCATTC

>Chr05 SNP-129

GGTTTAGCATCTTCTCTTTTTTCCCAAAGACCCCTGGTAACCCATATCCATTTCACCAAATTAGCAATTTGATGAAGTACTCTCTTTCTCTCTCTAGATCACAACAATAAGTCCTCCTCTCTCATTCTAGAAATTAGAAATCTAGAAAGGAAATTAATTTCTGCACTCTAAAATTTGATTTTTGCACTCCTATTTGAGTGCAGAAATCAACATCATTTCTGCACTCTTACTTTTACAAATTTTGGAGTGCAGAAAACAATTTCCTTTAGAAATTGCATTCCA

>Chr05 SNP-130

GGCTCTACTCTTCTCTTTTTTCCCAAAGACCGCTGGTAACCCATATCCATTTCACCAAATTAGCAATTTGATGAAGTACTGTCTTTCTCTCTCTAGATCACAACAACACGTCCTCCTCTCTCATTCTAGAAATTAGAAATCTAAAAAGAAAATTGATTTCTGCACTCTAAAATTTAATTTCTGCACTCCTATTTAAGTGCAGAAATCAACGCCATTTCTGCACTCCTACTTTTATAAGTTTTGGAGTGCAGAAATTAAATTTCCTTTAGAAATTGCATTCAG

>Chr05 SNP-131

TTCTTCGCACTCTTCTCTTTTTTCCCAAAGACCGCTGGTAACCCATATCCATTTCACCAAATTAGCAATTTGATGAAGTACTCTCTTTCTCTCTCTAGATCACAACAACACGTCCTCCTCTCTCATTCTAGAAATTAGAAATCTAGAGAGGAAATTGATTTCTGCACTCTAAAACTTGATTTCTGCGCTCCTATTTGAGTGCATAAATCAACACAATTTCTGCACTCCTACTTTTACAAATTTTGAAGTGCAGAAATTATTTTTCCTTTAGAAATTGCATTCAAGGATGACCAAAAA>Chr05 SNP-132

GGTTTCCATCTTCTCTTTTTTCCCAAAGACCGCTGGTAACCCATATCCATTTCACCAAATTAGCAATTTGATGAAGTACTCTCTTTCTCTCTCTAGATCACAACAACATGTCCTCCTCTCTCGTTCTAGAAATTAGAAATCTGGAGAGGAAATTGATTTCTGCACTCTAAAATTTGATTTCTGCGCTCTTATTTGAGTGCATAAATCAACACAATTTCTGCACCCCTACTTTTACAAATTTTGAAGTGCAGAAATTATTTTTCCTTTAGAAATTGCATTCAAGAATGACCAAAC

>Chr05 SNP-133

GGCGTACGATCTTCTCTTTTTTCCCAAAGACCGCTGGTAACCCATATCCATTTCACCAAATTAGCAATTTGATGAAGTACTCTCTTTCTCTCTCTAGATCACAACAACACGTCCTCCTCTCTCATTCTAGAAATTAGAAATCTAAAAAGAAAATTGATTTCTGCACTCTAAAATTTAATTTCTGCACTCCTATTTAAGTGCAGAAATCAACGCCATTTCTGCACTCCTACTTTTATAAGTTTTGGAGTGCAGAAATTAAATTTCCTTTAGAAATTGCTTCA

>Chr05 SNP-134

CGGTCTATCTTCTCTTTTTTCCCAAAGACCCCTGGTAACCCATATCCATTTCACCAAATTAGCAATTTGATGAAGTACTCTCTTTCTCTGTCTAGATCACAACAACACGTCCTCCTCTCTCATTCTAGAAATTAGAAATCTAGAAAGGAAATTGATTTCTGCACTCTAAAATTTGATTTCTGCACTCCTATTTGAGTGCAGATATCAACACCATTTCTGCACTCCTATTTTTACAAATTTTGAAGTGCAGAAATTATTTTTCCTTTAGAAATTGCATTCAAGAATGACCAAAA

>Chr05 SNP-135

GGAGCCATCATCCTCTCTTTTTTCCCAAAAGACCGCTGGTAACCCATATCCATTTCACCAAATTAGCAATTTGATGAAGTACTCTCTTTCTCTGTCTAGATCACAACAACATGTCCTCCTCTCTCGTTCTAGAAATTAGAAATCTGGAAAGAAAATTGATTTCTGCACTCTAAAATTTGATTTCTGCACTCTTATTTGAGTGCAGATATCAACACCATTTCTGCACTCCTATTTTTACAAATTTTGAAGTGCAGAAATTATTTTTCCTTTAGAAATTGCATCCCAGATGACCA

>Chr05 SNP-136

GGAGAGCAACTTCTCTTTCATCTTTTTTCCCAAGACCGCTGGTAACCCATATCCATTTCACCAAATTAGCAATTTGATGAAGTACTCTCTTTCTCTGTCTAGATCACAACAACATGTCCTCCTCTCTCGTTCTAGAAATTAGAAATCTGGAAAGAAAATTGATTTCTGCACTCTAAAATTTGATTTCTGCACTCTTATTTGAGTGCAGATATCAACACCATTTCTGCACTCCTATTTTTACAAATTTTGAAGTGCAGAAATTATTTTTCCTTTAGAAATTGG

>Chr05 SNP-137

GGCGTACGATCTTCTCTTTTTTCCCAAAGACCGCTGGTAACCCATATCCATTTCACCAAATTAGCAATTTGATGAAGTACTCTCTTTCTCTCTCTAGATCACAACAACACGTCCTCCTCTCTCATTCTAGAAATTAGAAATCTAAAAAGAAAATTGATTTCTGCACTCTAAAATTTAATTTCTGCACTCCTATTTAAGTGCAGAAATCAACGCCATTTCTGCACTCCTACTTTTATAAGTTTTGGAGTGCAGAAATTAAATTTCCTTTAGAAATTGCTTCA

>Chr05 SNP-138

CGCTCTACTCTTCTCTTTTTTCCCAAAGACCGCTGGTAACCCATATCCATTTCACCAAATTAGCAATTTGATGAAGTACTCTCTTTCTCTCTCTAGATCACAACAACATGTCCTCCTCTCTGGTTCTAGAAATTAGAAATCTGGAAAGAAAATTGATTTCTGCACTCTAAAATTTGATTTCTGCACTCTTATTTGAGTGTAGAAATCAACACCATTTCTGCACCCCTACTTTTACAAATTTTGAAGTGCAGAAATTATTTTTCCTTTAAAAATGGCTTCCAG

>Chr05 SNP-139

GGATTACTATCTTCTCTTTTTTCCCAAAGACCGCTGGTAACCCATATCCATTTCACCAAATTAGCAATTTGATGAAGTACTCTCTTTCTCTCTCTAGATCACAACAACACGTCCTCCTCTCTCATTCTAGATATTAGAAATCTAGAAAGGAAATTGATTTCTGCACTCTAAAATTTGATTTTTGCACTCCTATTTGAGTGCAGAAATCAACACAATTTCTGCACCCCTACTTTTACAAATTTTGAAGTGCAGAAATTATTTTTCCTTTAGAAATTGCTTCC

>Chr05 SNP-140

GGCTCTACTCTTCTCTTTTTTCCCAAAGACCGCTGGTAACCCATATCCATTTCACCAAATTAGCAATTTGATGAAGTACTGTCTTTCTCTCTCTAGATCACAACAACACGTCCTCCTCTCTCATTCTAGAAATTAGAAATCTAAAAAGAAAATTGATTTCTGCACTCTAAAATTTAATTTCTGCACTCCTATTTAAGTGCAGAAATCAACGCCATTTCTGCACTCCTACTTTTATAAGTTTTGGAGTGCAGAAATTAAATTTCCTTTAGAAATTGCATTCAG

>Chr05 SNP-141

GGTTTCCCATCTTCTCTTTTTTCCCAAAGACCCCTGGTAACCCATATCCATTTCACCAAATTAGCAATTTGATGAAGTACTCTCTTTCTCTCTCTAGATCACAACAACAAGTCCTCCTCTCTCATTCTAGAAATTAGAAATCTAGAAAGGAAATTGATTTCTGCACTCTAAAATTTGATTTTTGCACTCCTATTTGAGTGCAGAAATCAACATCATTTCTGCACTCTTACTTTTACAAATTTTGGAGTGCAGAAAATAATTTCCTTTAGAAATTGCATTCCAGAATGACCAAAA

>Chr05 SNP-142

GGCTACTACTCTTCTCTTTTTTCCCAAAGACCCCTGGTAACCCATATCCATTTCACCAAATTAGCAATTTGATGAAGTACTCTCTTTCTCTGTCTAGATCACAACAACACGTCCTCCTCTCTCATTCTAGAAATTAGAAATCTAGAAAGGAAATTGATTTCTGCACTCTAAAATTTGATTTCTGCACTCCTATTTGAGTGCAGATATCAACACCATTTCTGCACTCCTATTTTTACAAATTTTGAAGTGCAGAAATTATTTTTCCTTTAGAAATTGCATTC

>Chr05 SNP-143

CTTTTTGACTCTTCTCTTTTTTCCCAAAGACCGCTGGTAACCCATATCCATTTCACCAAATTAGCAATTTGATGAAGTACTGTCTTTCTCTCTCTAGATCACAACAACACGTCCTCCTCTCTCATTCTAGAAATTAGAAATCTAAAAAGAAAATTGATTTCTGCACTCTAAAATTTAATTTCTGCACTCCTATTTAAGTGCAGAAATCAACGCCATTTCTGCACTCCTACTTTTATAAGTTTTGGAGTGCAGAAATTAAATTTCCTTTAGAAATTGCATTCAGGAATGACCAAA
