# Supplementary material for: Genome-Wide Association Studies Provide Insights into the Genetic Determination of Flower and Leaf Traits of Actinidia eriantha
Source: Front Plant Sci. 2021 Aug 20;12:730890. doi: 10.3389/fpls.2021.730890 (PMC8417775; doi:10.3389/fpls.2021.730890)
Supplement: Supplementary file 5 [file Data_Sheet_1.ZIP › Supporting information files 1/Chr 24 SNP/Chr 24.docx]

Chr 24 SNP

F: GACACGTGGCATTAAGTAATG

R: CCATCACCATTCATAGCGAC

Product size: 357 bp

Tm: 52.6℃

>Chr24-1

GGCTTGGACATGCCGTGGCGCATCTGAGCGACCACGAATGGTCTCTGGAATCTGATGACCTCAACTTCCAGAAACTTCTGGCTGACAATCGCCGAGGATCTAGCGGCCACAATATCGCCACGTGTCCGATTCGCCACGAGGGCATCCGACGTGGCACCCCCCATTCGAGTGAAATTGATACGCAAATCGTTTTTATGGATCAACAGCGATTAGGGCACCTGCTCAGAGTCAAAGCGGGCCGAGAACTTTCTCTCTCCTGGGAGAAAAAAGCTGAGAAAATCGGAGAAGATTTTGAACTTTGATTTTTTGGTCGCTATGAATGGTGATGGGT

>Chr24-2

CGCATGGGTGCGTGGCGCATCTGAGCGACCACGAATGGTCTCTGGAATCTGATGACCTCAACTTCCAGAAACTTCTGGCTGACAATCGCCGAGGATCTGGCGGCCACAATATCGCCACGTGTCCGATTCGCCACGAGGGCATCCGACGTGGCACCCCCATTCGAGTGAAATTGATACGCAAATCGTTTTTATGGATCAACAGCGATTAGGGCACCTGCTCAGAGTCAAAGCGGGCCGAGAACTTCCTCTCTCCTGGGAGAAAAAAGCGGAGAAAATCGGAGAAAATCTTGAACTTTGATTTTTTGGTCGCTATAAT

>Chr24-3

CGCATCGATCTGCCGTGGCGCATCTGAGCGACCACGAATGGTCTCTGGAATCTGATGACCTCAACTTCCAGAAACTTCTGGCTGACAATCGCCGAGGATCTAGCGGCCACAATATCGCCACGTGTCCGATTCGCCACGAGGGCATCCGACGTGGCACCCCCCATTCGAGTGAAATTGATACGCAAATCGTTTTTATGGATCAACAGCGATTAGGGCACCTGCTCAGAGTCAAAGCGGGCCGAGAACTTTCTCTCTCCTGGGAGAAAAAAGCTGAGAAAATCGGAGAAAATTTTGAACTTTGATTTTTTGGTCGCTATGAATGGTGATGGAG

>Chr24-4

CGCATCGATTGACGTGGCGCATCTGAGCGACCACGAATGGTCTCTGGAATCTGATGACCTCAACTTCCAGAAACTTCTGGCTGACAATCGCCGAGGATCTAGCGGCCACAATATCGCCACGTGTCCGATTCGCCACGAGGGCATCCGACGTGGCACCCCCCATTCGAGTGAAATTGATACGCAAATCGTTTTTATGGATCAACAGCGATTAGGGCACCTGCTCAGAGTCAAAGCGGGCCGAGAACTTTCTCTCTCCTGGGAGAAAAAAGCTGAGAAAATCGGAGAAAATTTTGAACTTTGATTTTTTGGTCGCTATATTG

>Chr24-5

GGCATGATTGACGTGGCGCATCTGAGCGACCACGAATGGTCTCTGGAATCTGATGACCTCAACTTCCAGAAACTTCTGGCTGACAATCGCCGAGGATCTAGCGGCCACAATATCGCCACGTGTCCGATTCGCCACGAGGGCATCCGACGTGGCACCCCCCATTCGAGTGAAATTGATACGCAAATCGTTTTTATGGATCAACAGCGATTAGGGCACCTGCTCAGAGTCAAAGCGGGCCGAGAACTTTCTCTCTCCTGGGAGAAAAAAGCTGAGAAAATCGGAGAAAATTTTGAACTTTGATTTTTTGGTCGCTATGAATGGTGATGGA

>Chr24-6

GGCATCGCTTGACGTGGCGCATCTGAGCGACCACGAATGGTCTCTGGAATCTGATGACCTCAACTTCCAGAAACTTCTGGCTGACAATCGCCGAGGATCTAGCGGCCACAATATCGCCACGTGTCCGATTCGCCACGAGGGCATCCGACGTGGCACCCCCCATTCGAGTGAAATTGATACGCAAATCGTTTTTATGGATCAACAGCGATTAGGGCACCTGCTCAGAGTCAAAGCGGGCCGAGAACTTTCTCTCTCCTGGGAGAAAAAAGCTGAGAAAATCGGAGAAAATTTTGAACTTTGATTTTTTTGTCGCTATAATTGGTGATGGAC

>Chr24-7

CGTTCGATGACGTGGCGCATCTGAGCGACCACGAATGGTCTCTGGAATCTGATGACCTCAACTTCCAGAAACTTCTGGCTGACAATCGCCGAGGATCTAGCGGCCACAATATCGCCACGTGTCCGATTCGCCACGAGGGCATCCGACGTGGCACCCCCCATTCGAGTGAAATTGATACGCAAATCGTTTTTATGGATCAACAGCGATTAGGGCACCTGCTCAGAGTCAAAGCGGGCCGAGAACTTTCTCTCTCCTGGGAGAAAAAAGCTGAGAAAATCGGAGAAAATTTTGAACTTTGATTTTTTGGTCGCTTGATG

>Chr24-8

CGCATCGATGCGTGGCGCATCTGAGCGACCACGAATGGTCTCTGGAATCTGATGACCTCAACTTCCAGAAACTTCTGGCTGACAATCGCCGAGGATCTGGCGGCCACAATATCGCCACGTGTCCGATTCGCCACGAGGGCATCCGACGTGGCACCCCCATTCGAGTGAAATTGATACGCAAATCGTTTTTATGGATCAACAGCGATTAGGGCACCTGCTCAGAGTCAAAGCGGGCCGAGAACTTCCTCTCTCCTGGGAGAAAAAAGCGGAGAAAATCGGAGAAAATCTTGAACTTTGATTTTTTGGTCGCTATAATTGGGTGATGGAAC

>Chr24-9

CTTGGGGATGACGTGGCGCATCTGAGCGACCACGAATGGTCTCTGGAATCTGATGACCTCAACTTCCAGAAACTTCTGGCTGACAATCGCCGAGGATCTAGCGGCCACAATATCGCCACGTGTCCGATTCGCCACGAGGGCATCCGACGTGGCACCCCCCATTCGAGTGAAATTGATACGCAAATCGTTTTTATGGATCAACAGCGATTAGGGCACCTGCTCAGAGTCAAAGCGGGCCGAGAACTTTCTCTCTCCTGGGAGAAAAAAGCTGAGAAAATCGGAGAAAATTTTGAACTTTGATTTTTTGGTCGCTATGAATGGTGATGGAA

>Chr24-10

GCTTTTTCTGACGTGGCGCATCTGAGCGACCACGAATGGTCTCTGGAATCTGATGACCTCAACTTCCAGAAACTTCTGGCTGACAATCGCCGAGGATCTAGCGGCCACAATATCGCCACGTGTCCGATTCGCCACGAGGGCATCCGACGTGGCACCCCCCATTCGAGTGAAATTGATACGCAAATCGTTTTTATGGATCAACAGCGATTAGGGCACCTGCTCAGAGTCAAAGCGGGCCGAGAACTTTCTCTCTCCTGGGAGAAAAAAGCTGAGAAAATCGGAGAAAATTTTGAACTTTGATTTTTTGGTCGCTATAATTGGT

>Chr24-11

GCAGGGGTTGACGTGGCGCATCTGAGCGACCACGAATGGTCTCTGGAATCTGATGACCTCAACTTCCAGAAACTTCTGGCTGACAATCGCCGAGGATCTAGCGGCCACAATATCGCCACGTGTCCGATTCGCCACGAGGGCATCCGACGTGGCACCCCCCATTCGAGTGAAATTGATACGCAAATCGTTTTTATGGATCAACAGCGATTAGGGCACCTGCTCAGAGTCAAAGCGGGCCGAGAACTTTCTCTCTCCTGGGAGAAAAAAGCTGAGAAAATCGGAGAAAATTTTGAACTTTGATTTTTTTGTCGCTATGAATGGTGATGGACC

>Chr24-12

GGGTATTCATGACGTGGCGCATCTGAGCGACCACGAATGGTCTCTGGAATCTGATGACCTCAACTTCCAGAAACTTCTGGCTGACAATCGCCGAGGATCTAGCGGCCACAATATCGCCACGTGTCCGATTCGCCACGAGGGCATCCGACGTGGCACCCCCCATTCGAGTGAAATTGATACGCAAATCGTTTTTATGGATCAACAGCGATTAGGGCACCTGCTCAGAGTCAAAGCGGGCCGAGAACTTTCTCTCTCCTGGGAGAAAAAAGCTGAGAAAATCGGAGAAAATTTTGAACTTTGATTTTTTGGTCGCTATGAATGGTGATGGAT

>Chr24-13

CGTAGGGGTTGACGTGGCGCATCTGAGCGACCACGAATGGTCTCTGGAATCGGATGACCTCAACTTCCAGAAACTTCTGGCTGACAATCGCCGAGGATCTAGCGGCCACAATATCGCCACGTGTCCGATTCGCCACGAGGGCATCCGACGTGGCACCCCCCATTCGAGTGAAATTGATACGCAAATCGTTTTTATGGATCAACAGCGATTAGGGCACCTGCTCAGAGTCAAAGCGGGCCGAGAACTTTCTCTCTCCTGGGAGAAAAAAGCTGAGAAAATCGGAGAAAATTTTGAACTTTGATTTTTTGGTCGCTATGAATGGTGATGGAA

>Chr24-14

GCTTTTTGATTGACGTGGCGCATCTGAGCGACCACGAATGGTCTCTGGAATCTGATGACCTCAACTTCCAGAAACTTCTGGCTGACAATCGCCGAGGATCTAGCGGCCACAATATCGCCACGTGTCCGATTCGCCACGAGGGCATCCGACGTGGCACCCCCCATTCGAGTGAAATTGATACGCAAATCGTTTTTATGGATCAACAGCGATTAGGGCACCTGCTCAGAGTCAAAGCGGGCCGAGAACTTTCTCTCTCCTGGGAGAAAAAAGCTGAGAAAATCGGAGAAGATTTTGAACTTTGATTTTTTGGTCGCTATGAATGGTGATGGAC

>Chr24-15

GCATTTGGTGACGTGGCGCATCTGAGCGACCACGAATGGTCTCTGGAATCTGATGACCTCAACTTCCAGAAACTTCTGGCTGACAATCGCCGAGGATCTAGCGGCCACAATATCGCCACGTGTCCGATTCGCCACGAGGGCATCCGACGTGGCACCCCCCATTCGAGTGAAATTGATACGCAAATCGTTTTTATGGATCAACAGCGATTAGGGCACCTGCTCAGAGTCAAAGCGGGCCGAGAACTTTCTCTCTCCTGGGAGAAAAAAGCTGAGAAAATCGGAGAAAATTTTGAACTTTGATTTTTTGGTCGCTTGATGGGGTGATGGAC

>Chr24-16

GGCTACTCTTGCCGTGGCGCATCTGAGCGACCACGAATGGTCTCTGGAATCTGATGACCTCAACTTCCAGAAACTTCTGGCTGACAATCGCCGAGGATCTAGCGGCCACAATATCGCCACGTGTCCGATTCGCCACGAGGGCATCCGACGTGGCACCCCCCATTCGAGTGAAATTGATACGCAAATCGTTTTTATGGATCAACAGCGATTAGGGCACCTGCTCAGAGTCAAAGCGGGCCGAGAACTTTCTCTCTCCTGGGAGAAAAAAGCTGAGAAAATCGGAGAAAATTTTGAACTTTGATTTTTTGGTCGCTATAAT

>Chr24-17

CGCTTTCTTGCCGTGGCGCATCTGAGCGACCACGAATGGTCTCTGGAATCTGATGACCTCAACTTCCAGAAACTTCTGGCTGACAATCGCCGAGGATCTAGCGGCCACAATATCGCCACGTGTCCGATTCGCCACGAGGGCATCCGACGTGGCACCCCCCATTCGAGTGAAATTGATACGCAAATCGTTTTTATGGATCAACAGCGATTAGGGCACCTGCTCAGAGTCAAAGCGGGCCGAGAACTTTCTCTCTCCTGGGAGAAAAAAGCTGAGAAAATCGGAGAAAATTTTGAACTTTGATTTTTTGGTCGCTATGAATGGTGATGGAGG

>Chr24-18

CGCTTTTCTTGACGTGGCGCATCTGAGCGACCACGAATGGTCTCTGGAATCTGATGACCTCAACTTCCAGAAACTTCTGGCTGACAATCGCCGAGGATCTAGCGGCCACAATATCGCCACGTGTCCGATTCGCCACGAGGGCATCCGACGTGGCACCCCCCATTCGAGTGAAATTGATACGCAAATCGTTTTTATGGATCAACAGCGATTAGGGCACCTGCTCAGAGTCAAAGCGGGCCGAGAACTTTCTCTCTCCTGGGAGAAAAAAGCTGAGAAAATCGGAGAAAATTTTGAACTTTGATTTTTTGGTCGCTATGAATGGTGATGGACC

>Chr24-19

CGCTTTTGATGACGTGGCGCATCTGAGCGACCACGAATGGTCTCTGGAATCTGATGACCTCAACTTCCAGAAACTTCTGGCTGACAATCGCCGAGGATCTAGCGGCCACAATATCGCCACGTGTCCGATTCGCCACGAGGGCATCCGACGTGGCACCCCCCATTCGAGTGAAATTGATACGCAAATCGTTTTTATGGATCAACAGCGATTAGGGCACCTGCTCAGAGTCAAAGCGGGCCGAGAACTTTCTCTCTCCTGGGAGAAAAAAGCTGAGAAAATCGGAGAAAATTTTGAACTTTGATTTTTTGGTCGCTATAATGGGTGATGGA

>Chr24-20

CGCATTCATGACGTGGCGCATCTGAGCGACCACGAATGGTCTCTGGAATCTGATGACCTCAACTTCCAGAAACTTCTGGCTGACAATCGCCGAGGATCTAGCGGCCACAATATCGCCACGTGTCCGATTCGCCACGAGGGCATCCGACGTGGCACCCCCCATTCGAGTGAAATTGATACGCAAATCGTTTTTATGGATCAACAGCGATTAGGGCACCTGCTCAGAGTCAAAGCGGGCCGAGAACTTTCTCTCTCCTGGGAGAAAAAAGCTGAGAAAATCGGAGAAAATTTTGAACTTTGATTTTTTGGTCGCTATAAT

>Chr24-21

CGCTTTTTTGTGACGTGGCGCATCTGAGCGACCACGAATGGTCTCTGGAATCTGATGACCTCAACTTCCAGAAACTTCTGGCTGACAATCGCCGAGGATCTAGCGGCCACAATATCGCCACGTGTCCGATTCGCCACGAGGGCATCCGACGTGGCACCCCCCATTCGAGTGAAATTGATACGCAAATCGTTTTTATGGATCAACAGCGATTAGGGCACCTGCTCAGAGTCAAAGCGGGCCGAGAACTTTCTCTCTCCTGGGAGAAAAAAGCTGAGAAAATCGGAGAAAATTTTGAACTTTGATTTTTTGGTCGCTATGAATGGTGATGGAC

>Chr24-22

GGGTATTCATGACGTGGCGCATCTGAGCGACCACGAATGGTCTCTGGAATCTGATGACCTCAACTTCCAGAAACTTCTGGCTGACAATCGCCGAGGATCTAGCGGCCACAATATCGCCACGTGTCCGATTCGCCACGAGGGCATCCGACGTGGCACCCCCCATTCGAGTGAAATTGATACGCAAATCGTTTTTATGGATCAACAGCGATTAGGGCACCTGCTCAGAGTCAAAGCGGGCCGAGAACTTTCTCTCTCCTGGGAGAAAAAAGCTGAGAAAATCGGAGAAAATTTTGAACTTTGATTTTTTGGTCGCTATGAATGGTGATGGAT

>Chr24-23

GCTTTTTGTTGACGTGGCGCATCTGAGCGACCACGAATGGTCTCTGGAATCTGATGACCTCAACTTCCAGAAACTTCTGGCTGACAATCGCCGAGGATCTAGCGGCCACAATATCGCCACGTGTCCGATTCGCCACGAGGGCATCCGACGTGGCACCCCCCATTCGAGTGAAATTGATACGCAAATCGTTTTTATGGATCAACAGCGATTAGGGCACCTGCTCAGAGTCAAAGCGGGCCGAGAACTTTCTCTCTCCTGGGAGAAAAAAGCTGAGAAAATCGGAGAAAATTTTGAACTTTGATTTTTTGGTCGCTATAATGGGTGATGGAA

>Chr24-24

GCTTTTTGATTGACGTGGCGCATCTGAGCGACCACGAATGGTCTCTGGAATCTGATGACCTCAACTTCCAGAAACTTCTGGCTGACAATCGCCGAGGATCTAGCGGCCACAATATCGCCACGTGTCCGATTCGCCACGAGGGCATCCGACGTGGCACCCCCCATTCGAGTGAAATTGATACGCAAATCGTTTTTATGGATCAACAGCGATTAGGGCACCTGCTCAGAGTCAAAGCGGGCCGAGAACTTTCTCTCTCCTGGGAGAAAAAAGCTGAGAAAATCGGAGAAGATTTTGAACTTTGATTTTTTGGTCGCTATGAATGGTGATGGAC

>Chr24-25

CTTTGTGTCTGCCGTGGCGCATCTGAGCGACCACGAATGGTCTCTGGAATCTGATGACCTCAACTTCCAGAAACTTCTGGCTGACAATCGCCGAGGATCTAGCGGCCACAATATCGCCACGTGTCCGATTCGCCACGAGGGCATCCGACGTGGCACCCCCCATTCGAGTGAAATTGATACGCAAATCGTTTTTATGGATCAACAGCGATTAGGGCACCTGCTCAGAGTCAAAGCGGGCCGAGAACTTTCTCTCTCCTGGGAGAAAAAAGCTGAGAAAATCGGAGAAAATTTTGAACTTTGATTTTTTGGTCGCTATGAATGGTGATGGAAA

>Chr24-26

CCATGGTATGACGTGGCGCATCTGAGCGACCACGAATGGTCTCTGGAATCTGATGACCTCAACTTCCAGAAACTTCTGGCTGACAATCGCCGAGGATCTAGCGGCCACAATATCGCCACGTGTCCGATTCGCCACGAGGGCATCCGACGTGGCACCCCCCATTCGAGTGAAATTGATACGCAAATCGTTTTTATGGATCAACAGCGATTAGGGCACCTGCTCAGAGTCAAAGCGGGCCGAGAACTTTCTCTCTCCTGGGAGAAAAAAGCTGAGAAAATCGGAGAAGATTTTGAACTTTGATTTTTTGGTCGCTATGAATGGTGATGGA

>Chr24-27

GCTTTGGGTTGACGTGGCGCATCTGAGCGACCACGAATGGTCTCTGGAATCTGATGACCTCAACTTCCAGAAACTTCTGGCTGACAATCGCCGAGGATCTAGCGGCCACAATATCGCCACGTGTCCGATTCGCCACGAGGGCATCCGACGTGGCACCCCCCATTCGAGTGAAATTGATACGCAAATCGTTTTTATGGATCAACAGCGATTAGGGCACCTGCTCAGAGTCAAAGCGGGCCGAGAACTTTCTCTCTCCTGGGAGAAAAAAGCTGAGAAAATCGGAGAAAATTTTGAACTTTGATTTTTTGGTCGCTATAATGGGTGATGGAC

>Chr24-28

GCTCTCCTTTGCCGTGGCGCATCTGAGCGACCACGAATGGTCTCTGGAATCTGATGACCTCAACTTCCAGAAACTTCTGGCTGACAATCGCCGAGGATCTAGCGGCCACAATATCGCCACGTGTCCGATTCGCCACGAGGGTATCCGACGTGGCACCCCCCGTTCGAGTGAAATTGATACGCAAATCGTTTTTATGGATCAACAGCGATTAGGGCACCTGCTCAGAGTCAAAGCGGGCCGAGAACTTTCTCTCTCCTGGGAGAAAAAAGCTGAGAAAATCGGAGAAAATTTTGAACTTTGATTTTTTGGTCGCTATGAATGGGTGATGGAA

>Chr24-29

CGTTTTGGGTTGCCGTGGCGCATCTGAGCGACCACGAATGGTCTCTGGAATCTGATGACCTCAACTTCCAGAAACTTCTGGCTGACAATCGCCGAGGATCTAGCGGCCACAATATCGCCACGTGTCCGATTCGCCACGAGGGCATCCGACGTGGCACCCCCCATTCGAGTGAAATTGATACGCAAATCGTTTTTATGGATCAACAGCGATTAGGGCACCTGCTCAGAGTCAAAGCGGGCCGAGAACTTTCTCTCTCCTGGGAGAAAAAAGCTGAGAAAATCGGAGAAAATTTTGAACTTTGATTTTTTGGTCGCTATGAATGGTGATGGAC

>Chr24-30

GCTTTTCTCTGACGTGGCGCATCTGAGCGACCACGAATGGTCTCTGGAATCTGATGACCTCAACTTCCAGAAACTTCTGGCTGACAATCGCCGAGGATCTAGCGGCCACAATATCGCCACGTGTCCGATTCGCCACGAGGGCATCCGACGTGGCACCCCCCATTCGAGTGAAATTGATACGCAAATCGTTTTTATGGATCAACAGCGATTAGGGCACCTGCTCAGAGTCAAAGCGGGCCGAGAACTTTCTCTCTCCTGGGAGAAAAAAGCTGAGAAAATCGGAGAAAATTTTGAACTTTGATTTTTTGGTCGCTATAATGGG

>Chr24-31

GCATCTGATGCCGTGGCGCATCTGAGCGACCACGAATGGTCTCTGGAATCTGATGACCTCAACTTCCAGAAACTTCTGGCTGACAATCGCCGAGGATCTAGCGGCCACAATATCGCCACGTGTCCGATTCGCCACGAGGGTATCCGACGTGGCACCCCCCGTTCGAGTGAAATTGATACGCAAATCGTTTTTATGGATCAACAGCGATTAGGGCACCTGCTCAGAGTCAAAGCGGGCCGAGAACTTTCTCTCTCCTGGGAGAAAAAAGCTGAGAAAATCGGAGAAAATTTTGAACTTTGATTTTTTGGTCGCTATAATG

>Chr24-32

GCTTTCGATGACGTGGCGCATCTGAGCGACCACGAATGGTCTCTGGAATCTGATGACCTCAACTTCCAGAAACTTCTGGCTGACAATCGCCGAGGATCTAGCGGCCACAATATCGCCACGTGTCCGATTCGCCACGAGGGCATCCGACGTGGCACCCCCCATTCGAGTGAAATTGATACGCAAATCGTTTTTATGGATCAACAGCGATTAGGGCACCTGCTCAGAGTCAAAGCGGGCCGAGAACTTTCTCTCTCCTGGGAGAAAAAAGCTGAGAAAATCGGAGAAAATTTTGAACTTTGATTTTTTGGTCGCTATGAATGGGTGATGGAA

>Chr24-33

CTCAGTTTTTGCCGTGGCGCATCTGAGCGACCACGAATGGTCTCTGGAATCTGATGACCTCAACTTCCAGAAACTTCTGGCTGACAATCGCCGAGGATCTAGCGGCCACAATATCGCCACGTGTCCGATTCGCCACGAGGGCATCCGACGTGGCACCCCCCATTCGAGTGAAATTGATACGCAAATCGTTTTTATGGATCAACAGCGATTAGGGCACCTGCTCAGAGTCAAAGCGGGCCGAGAACTTTCTCTCTCCTGGGAGAAAAAAGCTGAGAAAATCGGAGAAAATTTTGAACTTTGATTTTTTGGTCGCTATGAATGGTGATGGAC

>Chr24-34

GCGTTTTGTTGACGTGGCGCATCTGAGCGACCACGAATGGTCTCTGGAATCTGATGACCTCAACTTCCAGAAACTTCTGGCTGACAATCGCCGAGGATCTAGCGGCCACAATATCGCCACGTGTCCGATTCGCCACGAGGGCATCCGACGTGGCACCCCCCATTCGAGTGAAATTGATACGCAAATCGTTTTTATGGATCAACAGCGATTAGGGCACCTGCTCAGAGTCAAAGCGGGCCGAGAACTTTCTCTCTCCTGGGAGAAAAAAGCTGAGAAAATCGGAGAAAATTTTGAACTTTGATTTTTTGGTCGCTATGAATGGTGATGGAG

>Chr24-35

TCTTTTCGGTGACGTGGCGCATCTGAGCGACCACGAATGGTCTCTGGAATCTGATGACCTCAACTTCCAGAAACTTCTGGCTGACAATCGCCGAGGATCTAGCGGCCACAATATCGCCACGTGTCCGATTCGCCACGAGGGCATCCGACGTGGCACCCCCCATTCGAGTGAAATTGATACGCAAATCGTTTTTATGGATCAACAGCGATTAGGGCACCTGCTCAGAGTCAAAGCGGGCCGAGAACTTTCTCTCTCCTGGGAGAAAAAAGCTGAGAAAATCGGAGAAAATTTTGAACTTTGATTTTTTGGTCGCTATGAATGGGTGATGGA

>Chr24-36

CCTTTTTGTTTGACGTGGCGCATCTGAGCGACCACGAATGGTCTCTGGAATCTGATGACCTCAACTTCCAGAAACTTCTGGCTGACAATCGCCGAGGATCTAGCGGCCACAATATCGCCACGTGTCCGATTCGCCACGAGGGCATCCGACGTGGCACCCCCCATTCGAGTGAAATTGATACGCAAATCGTTTTTATGGATCAACAGCGATTAGGGCACCTGCTCAGAGTCAAAGCGGGCCGAGAACTTTCTCTCTCCTGGGAGAAAAAAGCTGAGAAAATCGGAGAAAATTTTGAACTTTGATTTTTTGGTCGCTATAATGGGT

>Chr24-37

GCATCTCTCTGCCGTGGCGCATCTGAGCGACCACGAATGGTCTCTGGAATCTGATGACCTCAACTTCCAGAAACTTCTGGCTGACAATCGCCGAGGATCTAGCGGCCACAATATCGCCACGTGTCCGATTCGCCACGAGGGTATCCGACGTGGCACCCCCCGTTCGAGTGAAATTGATACGCAAATCGTTTTTATGGATCAACAGCGATTAGGGCACCTGCTCAGAGTCAAAGCGGGCCGAGAACTTTCTCTCTCCTGGGAGAAAAAAGCTGAGAAAATCGGAGAAAATTTTGAACTTTGATTTTTTGGTCGCTATAAT

>Chr24-38

GTTTTGGTTTGACGTGGCGCATCTGAGCGACCACGAATGGTCTCTGGAATCTGATGACCTCAACTTCCAGAAACTTCTGGCTGACAATCGCCGAGGATCTAGCGGCCACAATATCGCCACGTGTCCGATTCGCCACGAGGGCATCCGACGTGGCACCCCCCATTCGAGTGAAATTGATACGCAAATCGTTTTTATGGATCAACAGCGATTAGGGCACCTGCTCAGAGTCAAAGCGGGCCGAGAACTTTCTCTCTCCTGGGAGAAAAAAGCTGAGAAAATCGGAGAAAATTTTGAACTTTGATTTTTTGGTCGCTATGAATGGTGATGGAC

>Chr24-39

GTTTTTTGATGCCGTGGCGCATCTGAGCGACCACGAATGGTCTCTGGAATCTGATGACCTCAACTTCCAGAAACTTCTGGCTGACAATCGCCGAGGATCTAGCGGCCACAATATCGCCACGTGTCCGATTCGCCACGAGGGCATCCGACGTGGCACCCCCCATTCGAGTGAAATTGATACGCAAATCGTTTTTATGGATCAACAGCGATTAGGGCACCTGCTCAGAGTCAAAGCGGGCCGAGAACTTTCTCTCTCCTGGGAGAAAAAAGCTGAGAAAATCGGAGAAAATTTTGAACTTTGATTTTTTGGTCGCTATATT

>Chr24-40

GCTTCGGTTTGCCGTGGCGCATCTGAGCGACCACGAATGGTCTCTGGAATCTGATGACCTCAACTTCCAGAAACTTCTGGCTGACAATCGCCGAGGATCTAGCGGCCACAATATCGCCACGTGTCCGATTCGCCACGAGGGTATCCGACGTGGCACCCCCCGTTCGAGTGAAATTGATACGCAAATCGTTTTTATGGATCAACAGCGATTAGGGCACCTGCTCAGAGTCAAAGCGGGCCGAGAACTTTCTCTCTCCTGGGAGAAAAAAGCTGAGAAAATCGGAGAAAATTTTGAACTTTGATTTTTTGGTCGCTATAAT

>Chr24-41

GGAGGTGTGCCGTGGCGCATCTGAGCGACCACGAATGGTCTCTGGAATCTGATGACCTCAACTTCCAGAAACTTCTGGCTGACAATCGCCGAGGATCTAGCGGCCACAATATCGCCACGTGTCCGATTCGCCACGAGGGCATCCGACGTGGCACCCCCCATTCGAGTGAAATTGATACGCAAATCGTTTTTATGGATCAACAGCGATTAGGGCACCTGCTCAGAGTCAAAGCGGGCCGAGAACTTTCTCTCTCCTGGGAGAAAAAAGCTGAGAAAATCGGAGAAAATTTTGAACTTTGATTTTTTGGTCGCTATGAATGGTGATGGAA

>Chr24-42

CTTTTGGGGCTGCCGTGGCGCATCTGAGCGACCACGAATGGTCTCTGGAATCTGATGACCTCAACTTCCAGAAACTTCTGGCTGACAATCGCCGAGGATCTAGCGGCCACAATATCGCCACGTGTCCGATTCGCCACGAGGGCATCCGACGTGGCACCCCCCATTCGAGTGAAATTGATACGCAAATCGTTTTTATGGATCAACAGCGATTAGGGCACCTGCTCAGAGTCAAAGCGGGCCGAGAACTTTCTCTCTCCTGGGAGAAAAAAGCTGAGAAAATCGGAGAAAATTTTGAACTTTGATTTTTTGGTCGCTATGAATGGTGATGGAA

>Chr24-43

GGCTTCTTTTGCCGTGGCGCATCTGAGCGACCACGAATGGTCTCTGGAATCTGATGACCTCAACTTCCAGAAACTTCTGGCTGACAATCGCCGAGGATCTAGCGGCCACAATATCGCCACGTGTCCGATTCGCCACGAGGGCATCCGACGTGGCACCCCCCATTCGAGTGAAATTGATACGCAAATCGTTTTTATGGATCAACAGCGATTAGGGCACCTGCTCAGAGTCAAAGCGGGCCGAGAACTTTCTCTCTCCTGGGAGAAAAAAGCTGAGAAAATCGGAGAAAATTTTGAACTTTGATTTTTTGGTCGCTATGAATGGTGATGGACT

>Chr24-44

GTATTGTGCCGTGGCGCATCTGAGCGACCACGAATGGTCTCTGGAATCTGATGACCTCAACTTCCAGAAACTTCTGGCTGACAATCGCCGAGGATCTAGCGGCCACAATATCGCCACGTGTCCGATTCGCCACGAGGGCATCCGACGTGGCACCCCCCATTCGAGTGAAATTGATACGCAAATCGTTTTTATGGATCAACAGCGATTAGGGCACCTGCTCAGAGTCAAAGCGGGCCGAGAACTTTCTCTCTCCTGGGAGAAAAAAGCTGAGAAAATCGGAGAAAATTTTGAACTTTGATTTTTTGGTCGCTATAATGGGTGATGGACG

>Chr24-45

CTTAGGGTTTGACGTGGCGCATCTGAGCGACCACGAATGGTCTCTGGAATCTGATGACCTCAACTTCCAGAAACTTCTGGCTGACAATCGCCGAGGATCTAGCGGCCACAATATCGCCACGTGTCCGATTCGCCACGAGGGCATCCGACGTGGCACCCCCCATTCGAGTGAAATTGATACGCAAATCGTTTTTATGGATCAACAGCGATTAGGGCACCTGCTCAGAGTCAAAGCGGGCCGAGAACTTTCTCTCTCCTGGGAGAAAAAAGCTGAGAAAATCGGAGAAAATTTTGAACTTTGATTTTTTGGTCGCTATGAATGGTGATGGATT

>Chr24-46

GCATTCGTTGCCGTGGCGCATCTGAGCGACCACGAATGGTCTCTGGAATCTGATGACCTCAACTTCCAGAAACTTCTGGCTGACAATCGCCGAGGATCTAGCGGCCACAATATCGCCACGTGTCCGATTCGCCACGAGGGCATCCGACGTGGCACCCCCCATTCGAGTGAAATTGATACGCAAATCGTTTTTATGGATCAACAGCGATTAGGGCACCTGCTCAGAGTCAAAGCGGGCCGAGAACTTTCTCTCTCCTGGGAGAAAAAAGCTGAGAAAATCGGAGAAAATTTTGAACTTTGATTTTTTGGTCGCTATAATTGGGTGATGG

>Chr24-47

GCAGTGATTGCCGTGGCGCATCTGAGCGACCACGAATGGTCTCTGGAATCTGATGACCTCAACTTCCAGAAACTTCTGGCTGACAATCGCCGAGGATCTAGCGGCCACAATATCGCCACGTGTCCGATTCGCCACGAGGGCATCCGACGTGGCACCCCCCATTCGAGTGAAATTGATACGCAAATCGTTTTTATGGATCAACAGCGATTAGGGCACCTGCTCAGAGTCAAAGCGGGCCGAGAACTTTCTCTCTCCTGGGAGAAAAAAGCTGAGAAAATCGGAGAAAATTTTGAACTTTGATTTTTTGGTCGCTATAAT

>Chr24-48

GCTCTCCTTTGCCGTGGCGCATCTGAGCGACCACGAATGGTCTCTGGAATCTGATGACCTCAACTTCCAGAAACTTCTGGCTGACAATCGCCGAGGATCTAGCGGCCACAATATCGCCACGTGTCCGATTCGCCACGAGGGTATCCGACGTGGCACCCCCCGTTCGAGTGAAATTGATACGCAAATCGTTTTTATGGATCAACAGCGATTAGGGCACCTGCTCAGAGTCAAAGCGGGCCGAGAACTTTCTCTCTCCTGGGAGAAAAAAGCTGAGAAAATCGGAGAAAATTTTGAACTTTGATTTTTTGGTCGCTATGAATGGGTGATGGAA

>Chr24-49

CCTAGTGATTGACGTGGCGCATCTGAGCGACCACGAATGGTCTCTGGAATCTGATGACCTCAACTTCCAGAAACTTCTGGCTGACAATCGCCGAGGATCTAGCGGCCACAATATCGCCACGTGTCCGATTCGCCACGAGGGCATCCGACGTGGCACCCCCCATTCGAGTGAAATTGATACGCAAATCGTTTTTATGGATCAACAGCGATTAGGGCACCTGCTCAGAGTCAAAGCGGGCCGAGAACTTTCTCTCTCCTGGGAGAAAAAAGCTGAGAAAATCGGAGAAAATTTTGAACTTTGATTTTTTGGTCGCTATGAATGGTGATGGAC

>Chr24-50

CACTCCTGCCGTGGCGCATCTGAGCGACCACGAATGGTCTCTGGAATCTGATGACCTCAACTTCCAGAAACTTCTGGCTGACAATCGCCGAGGATCTAGCGGCCACAATATCGCCACGTGTCCGATTCGCCACGAGGGTATCCGACGTGGCACCCCCCGTTCGAGTGAAATTGATACGCAAATCGTTTTTATGGATCAACAGCGATTAGGGCACCTGCTCAGAGTCAAAGCGGGCCGAGAACTTTCTCTCTCCTGGGAGAAAAAAGCTGAGAAAATCGGAGAAAATTTTGAACTTTGATTTTTTGGTCGCTTAAT

>Chr24-51

GCTTCGGGTTGACGTGGCGCATCTGAGCGACCACGAATGGTCTCTGGAATCTGATGACCTCAACTTCCAGAAACTTCTGGCTGACAATCGCCGAGGATCTAGCGGCCACAATATCGCCACGTGTCCGATTCGCCACGAGGGCATCCGACGTGGCACCCCCCATTCGAGTGAAATTGATACGCAAATCGTTTTTATGGATCAACAGCGATTAGGGCACCTGCTCAGAGTCAAAGCGGGCCGAGAACTTTCTCTCTCCTGGGAGAAAAAAGCTGAGAAAATCGGAGAAGATTTTGAACTTTGATTTTTTGGTCGCTTGATTGGGTGATGGACC

>Chr24-52

GCTTTGTTTTTTGACGTGGCGCATCTGAGCGACCACGAATGGTCTCTGGAATCTGATGACCTCAACTTCCAGAAACTTCTGGCTGACAATCGCCGAGGATCTAGCGGCCACAATATCGCCACGTGTCCGATTCGCCACGAGGGCATCCGACGTGGCACCCCCCATTCGAGTGAAATTGATACGCAAATCGTTTTTATGGATCAACAGCGATTAGGGCACCTGCTCAGAGTCAAAGCGGGCCGAGAACTTTCTCTCTCCTGGGAGAAAAAAGCTGAGAAAATCGGAGAAGATTTTGAACTTTGATTTTTTGGTCGCTATGAATGGTGATGGACT

>Chr24-53

GCTTTTATGACGTGGCGCATCTGAGCGACCACGAATGGTCTCTGGAATCTGATGACCTCAACTTCCAGAAACTTCTGGCTGACAATCGCCGAGGATCTAGCGGCCACAATATCGCCACGTGTCCGATTCGCCACGAGGGCATCCGACGTGGCACCCCCCATTCGAGTGAAATTGATACGCAAATCGTTTTTATGGATCAACAGCGATTAGGGCACCTGCTCAGAGTCAAAGCGGGCCGAGAACTTTCTCTCTCCTGGGAGAAAAAAGCTGAGAAAATCGGAGAAAATTTTGAACTTTGATTTTTTGGTCGCTATAAT

>Chr24-54

CCCATTCGTCTGCCGTGGCGCATCTGAGCGACCACGAATGGTCTCTGGAATCTGATGACCTCAACTTCCAGAAACTTCTGGCTGACAATCGCCGAGGATCTAGCGGCCACAATATCGCCACGTGTCCGATTCGCCACGAGGGCATCCGACGTGGCACCCCCCATTCGAGTGAAATTGATACGCAAATCGTTTTTATGGATCAACAGCGATTAGGGCACCTGCTCAGAGTCAAAGCGGGCCGAGAACTTTCTCTCTCCTGGGAGAAAAAAGCTGAGAAAATCGGAGAAAATTTTGAACTTTGATTTTTTGGTCGCTATAAA

>Chr24-55

GGCACTGGTTTGACGTGGCGCATCTGAGCGACCACGAATGGTCTCTGGAATCTGATGACCTCAACTTCCAGAAACTTCTGGCTGACAATCGCCGAGGATCTAGCGGCCACAATATCGCCACGTGTCCGATTCGCCACGAGGGTATCCGACGTGGCACCCCCCGTTCGAGTGAAATTGATACGCAAATCGTTTTTATGGATCAACAGCGATTAGGGCACCTGCTCAGAGTCAAAGCGGGCCGAGAACTTTCTCTCTCCTGGGAGAAAAAAGCTGAGAAAATCGGAGAAAATTTTGAACTTTGATTTTTTGGTCGCTTAATGGGGTGATGGAC

>Chr24-56

GCATCGGATGCGTGGCGCATCTGAGCGACCACGAATGGTCTCTGGAATCTGATGACCTCAACTTCCAGAAACTTCTGGCTGACAATCGCCGAGGATCTAGCGGCCACAATATCGCCACGTGTCCGATTCGCCACGAGGGCATCCGACGTGGCACCCCCCATTCGAGTGAAATTGATACGCAAATCGTTTTTATGGATCAACAGCGATTAGGGCACCTGCTCAGAGTCAAAGCGGGCCGAGAACTTTCTCTCTCCTGGGAGAAAAAAGCTGAGAAAATCGGAGAAAATTTTGAACTTTGATTTTTTGGTCGCTTGATGGGG

>Chr24-57

CGTATGGTTTGACGTGGCGCATCTGAGCGACCACGAATGGTCTCTGGAATCTGATGACCTCAACTTCCAGAAACTTCTGGCTGACAATCGCCGAGGATCTAGCGGCCACAATATCGCCACGTGTCCGATTCGCCACGAGGGCATCCGACGTGGCACCCCCCATTCGAGTGAAATTGATACGCAAATCGTTTTTATGGATCAACAGCGATTAGGGCACCTGCTCAGAGTCAAAGCGGGCCGAGAACTTTCTCTCTCCTGGGAGAAAAAAGCTGAGAAAATCGGAGAAAATTTTGAACTTTGATTTTTTGGTCGCTATGAATGGTGATGGACCC

>Chr24-58

GGCAGGGGTGCCGTGGCGCATCTGAGCGACCACGAATGGTCTCTGGAATCTGATGACCTCAACTTCCAGAAACTTCTGGCTGACAATCGCCGAGGATCTAGCGGCCACAATATCGCCACGTGTCCGATTCGCCACGAGGGCATCCGACGTGGCACCCCCCATTCGAGTGAAATTGATACGCAAATCGTTTTTATGGATCAACAGCGATTAGGGCACCTGCTCAGAGTCAAAGCGGGCCGAGAACTTTCTCTCTCCTGGGAGAAAAAAGCTGAGAAAATCGGAGAAAATTTTGAACTTTGATTTTTTGGTCGCTATGAATGGTGATGGAAT

>Chr24-59

TGCATCGGGTTTGACGTGGCGCATCTGAGCGACCACGAATGGTCTCTGGAATCTGATGACCTCAACTTCCAGAAACTTCTGGCTGACAATCGCCGAGGATCTAGCGGCCACAATATCGCCACGTGTCCGATTCGCCACGAGGGTATCCGACGTGGCACCCCCCGTTCGAGTGAAATTGATACGCAAATCGTTTTTATGGATCAACAGCGATTAGGGCACCTGCTCAGAGTCAAAGCGGGCCGAGAACTTTCTCTCTCCTGGGAGAAAAAAGCTGAGAAAATCGGAGAAAATTTTGAACTTTGATTTTTTGGTCGCTATAAT

>Chr24-60

GCATCTGATGCCGTGGCGCATCTGAGCGACCACGAATGGTCTCTGGAATCTGATGACCTCAACTTCCAGAAACTTCTGGCTGACAATCGCCGAGGATCTAGCGGCCACAATATCGCCACGTGTCCGATTCGCCACGAGGGTATCCGACGTGGCACCCCCCGTTCGAGTGAAATTGATACGCAAATCGTTTTTATGGATCAACAGCGATTAGGGCACCTGCTCAGAGTCAAAGCGGGCCGAGAACTTTCTCTCTCCTGGGAGAAAAAAGCTGAGAAAATCGGAGAAAATTTTGAACTTTGATTTTTTGGTCGCTATAATG

>Chr24-61

GGCATTGGGTTTGCCGTGGCGCATCTGAGCGACCACGAATGGTCTCTGGAATCTGATGACCTCAACTTCCAGAAACTTCTGGCTGACAATCGCCGAGGATCTAGCGGCCACAATATCGCCACGTGTCCGATTCGCCACGAGGGCATCCGACGTGGCACCCCCCATTCGAGTGAAATTGATACGCAAATCGTTTTTATGGATCAACAGCGATTAGGGCACCTGCTCAGAGTCAAAGCGGGCCGAGAACTTTCTCTCTCCTGGGAGAAAAAAGCTGAGAAAATCGGAGAAAATTTTGAACTTTGATTTTTTGGTCGCTATAATGGGTGATGGA

>Chr24-62

GGCTTTTCGGTTGACGTGGCGCATCTGAGCGACCACGAATGGTCTCTGGAATCTGATGACCTCAACTTCCAGAAACTTCTGGCTGACAATCGCCGAGGATCTAGCGGCCACAATATCGCCACGTGTCCGATTCGCCACGAGGGCATCCGACGTGGCACCCCCCATTCGAGTGAAATTGATACGCAAATCGTTTTTATGGATCAACAGCGATTAGGGCACCTGCTCAGAGTCAAAGCGGGCCGAGAACTTTCTCTCTCCTGGGAGAAAAAAGCTGAGAAAATCGGAGAAAATTTTGAACTTTGATTTTTTGGTCGCTATAAT

>Chr24-63

GGTTCGGGGCTTGACGTGGCGCATCTGAGCGACCACGAATGGTCTCTGGAATCTGATGACCTCAACTTCCAGAAACTTCTGGCTGACAATCGCCGAGGATCTAGCGGCCACAATATCGCCACGTGTCCGATTCGCCACGAGGGCATCCGACGTGGCACCCCCCATTCGAGTGAAATTGATACGCAAATCGTTTTTATGGATCAACAGCGATTAGGGCACCTGCTCAGAGTCAAAGCGGGCCGAGAACTTTCTCTCTCCTGGGAGAAAAAAGCTGAGAAAATCGGAGAAAATTTTGAACTTTGATTTTTTGGTCGCTATAAT

>Chr24-64

GGACGGCTTGCCGTGGCGCATCTGAGCGACCACGAATGGTCTCTGGAATCTGATGACCTCAACTTCCAGAAACTTCTGGCTGACAATCGCCGAGGATCTAGCGGCCACAATATCGCCACGTGTCCGATTCGCCACGAGGGCATCCGACGTGGCACCCCCCATTCGAGTGAAATTGATACGCAAATCGTTTTTATGGATCAACAGCGATTAGGGCACCTGCTCAGAGTCAAAGCGGGCCGAGAACTTTCTCTCTCCTGGGAGAAAAAAGCTGAGAAAATCGGAGAAAATTTTGAACTTTGATTTTTTGGTCGCTATAATGGGGTGATGGAA

>Chr24-65

CGCATCGCGTTGACGTGGCGCATCTGAGCGACCACGAATGGTCTCTGGAATCTGATGACCTCAACTTCCAGAAACTTCTGGCTGACAATCGCCGAGGATCTAGCGGCCACAATATCGCCACGTGTCCGATTCGCCACGAGGGCATCCGACGTGGCACCCCCCATTCGAGTGAAATTGATACGCAAATCGTTTTTATGGATCAACAGCGATTAGGGCACCTGCTCAGAGTCAAAGCGGGCCGAGAACTTTCTCTCTCCTGGGAGAAAAAAGCTGAGAAAATCGGAGAAAATTTTGAACTTTGATTTTTTGGTCGCTATAATGGGTGATGGAATT

>Chr24-66

GCCACTGCGTGCCGTGGCGCATCTGAGCGACCACGAATGGTCTCTGGAATCTGATGACCTCAACTTCCAGAAACTTCTGGCTGACAATCGCCGAGGATCTAGCGGCCACAATATCGCCACGTGTCCGATTCGCCACGAGGGCATCCGACGTGGCACCCCCCATTCGAGTGAAATTGATACGCAAATCGTTTTTATGGATCAACAGCGATTAGGGCACCTGCTCAGAGTCAAAGCGGGCCGAGAACTTTCTCTCTCCTGGGAGAAAAAAGCTGAGAAAATCGGAGAAAATTTTGAACTTTGATTTTTTGGTCGCTATAAT

>Chr24-67

CACTCCTGCCGTGGCGCATCTGAGCGACCACGAATGGTCTCTGGAATCTGATGACCTCAACTTCCAGAAACTTCTGGCTGACAATCGCCGAGGATCTAGCGGCCACAATATCGCCACGTGTCCGATTCGCCACGAGGGTATCCGACGTGGCACCCCCCGTTCGAGTGAAATTGATACGCAAATCGTTTTTATGGATCAACAGCGATTAGGGCACCTGCTCAGAGTCAAAGCGGGCCGAGAACTTTCTCTCTCCTGGGAGAAAAAAGCTGAGAAAATCGGAGAAAATTTTGAACTTTGATTTTTTGGTCGCTTAAT

>Chr24-68

GCACTGGGTTGCCGTGGCGCATCTGAGCGACCACGAATGGTCTCTGGAATCTGATGACCTCAACTTCCAGAAACTTCTGGCTGACAATCGCCGAGGATCTAGCGGCCACAATATCGCCACGTGTCCGATTCGCCACGAGGGCATCCGACGTGGCACCCCCCATTCGAGTGAAATTGATACGCAAATCGTTTTTATGGATCAACAGCGATTAGGGCACCTGCTCAGAGTCAAAGCGGGCCGAGAACTTTCTCTCTCCTGGGAGAAAAAAGCTGAGAAAATCGGAGAAAATTTTGAACTTTGATTTTTTGGTCGCTATAAT

>Chr24-69

TGCATCGGGTTTGACGTGGCGCATCTGAGCGACCACGAATGGTCTCTGGAATCTGATGACCTCAACTTCCAGAAACTTCTGGCTGACAATCGCCGAGGATCTAGCGGCCACAATATCGCCACGTGTCCGATTCGCCACGAGGGTATCCGACGTGGCACCCCCCGTTCGAGTGAAATTGATACGCAAATCGTTTTTATGGATCAACAGCGATTAGGGCACCTGCTCAGAGTCAAAGCGGGCCGAGAACTTTCTCTCTCCTGGGAGAAAAAAGCTGAGAAAATCGGAGAAAATTTTGAACTTTGATTTTTTGGTCGCTATAAT

>Chr24-70

CACGGGGTGCCGTGGCGCATCTGAGCGACCACGAATGGTCTCTGGAATCTGATGACCTCAACTTCCAGAAACTTCTGGCTGACAATCGCCGAGGATCTAGCGGCCACAATATCGCCACGTGTCCGATTCGCCACGAGGGTATCCGACGTGGCACCCCCCGTTCGAGTGAAATTGATACGCAAATCGTTTTTATGGATCAACAGCGATTAGGGCACCTGCTCAGAGTCAAAGCGGGCCGAGAACTTTCTCTCTCCTGGGAGAAAAAAGCTGAGAAAATCGGAGAAAATTTTGAACTTTGATTTTTTGGTCGCTTAAT

>Chr24-71

CGCATTGCCTTGACGTGGCGCATCTGAGCGACCACGAATGGTCTCTGGAATCTGATGACCTCAACTTCCAGAAACTTCTGGCTGACAATCGCCGAGGATCTAGCGGCCACAATATCGCCACGTGTCCGATTCGCCACGAGGGCATCCGACGTGGCACCCCCCATTCGAGTGAAATTGATACGCAAATCGTTTTTATGGATCAACAGCGATTAGGGCACCTGCTCAGAGTCAAAGCGGGCCGAGAACTTTCTCTCTCCTGGGAGAAAAAAGCTGAGAAAATCGGAGAAAATTTTGAACTTTGATTTTTTGGTCGCTATAATGG

>Chr24-72

CACGGGGTGCCGTGGCGCATCTGAGCGACCACGAATGGTCTCTGGAATCTGATGACCTCAACTTCCAGAAACTTCTGGCTGACAATCGCCGAGGATCTAGCGGCCACAATATCGCCACGTGTCCGATTCGCCACGAGGGTATCCGACGTGGCACCCCCCGTTCGAGTGAAATTGATACGCAAATCGTTTTTATGGATCAACAGCGATTAGGGCACCTGCTCAGAGTCAAAGCGGGCCGAGAACTTTCTCTCTCCTGGGAGAAAAAAGCTGAGAAAATCGGAGAAAATTTTGAACTTTGATTTTTTGGTCGCTTAAT

>Chr24-73

GGCATGCGATGACGTGGCGCATCTGAGCGACCACGAATGGTCTCTGGAATCTGATGACCTCAACTTCCAGAAACTTCTGGCTGACAATCGCCGAGGATCTAGCGGCCACAATATCGCCACGTGTCCGATTCGCCACGAGGGTATCCGACGTGGCACCCCCCGTTCGAGTGAAATTGATACGCAAATCGTTTTTATGGATCAACAGCGATTAGGGCACCTGCTCAGAGTCAAAGCGGGCCGAGAACTTTCTCTCTCCTGGGAGAAAAAAGCTGAGAAAATCGGAGAAAATTTTGAACTTTGATTTTTTGGTCGCTATAATTGGGTGATGGAC

>Chr24-74

TTTTGCCTTGCCGTGGCGCATCTGAGCGACCACGAATGGTCTCTGGAATCTGATGACCTCAACTTCCAGAAACTTCTGGCTGACAATCGCCGAGGATCTAGCGGCCACAATATCGCCACGTGTCCGATTCGCCACGAGGGCATCCGACGTGGCACCCCCCATTCGAGTGAAATTGATACGCAAATCGTTTTTATGGATCAACAGCGATTAGGGCACCTGCTCAGAGTCAAAGCGGGCCGAGAACTTTCTCTCTCCTGGGAGAAAAAAGCTGAGAAAATCGGAGAAAATTTTGAACTTTGATTTTTTGGTCGCTATGAATGGTGATGGATTTTT

>Chr24-75

AGCATTGATGCCGTGGCGCATCTGAGCGACCACGAATGGTCTCTGGAATCTGATGACCTCAACTTCCAGAAACTTCTGGCTGACAATCGCCGAGGATCTAGCGGCCACAATATCGCCACGTGTCCGATTCGCCACGAGGGCATCCGACGTGGCACCCCCCATTCGAGTGAAATTGATACGCAAATCGTTTTTATGGATCAACAGCGATTAGGGCACCTGCTCAGAGTCAAAGCGGGCCGAGAACTTTCTCTCTCCTGGGAGAAAAAAGCTGAGAAAATCGGAGAAAATTTTGAACTTTGATTTTTTGGTCGCTATATT

>Chr24-76

GCTGGGGTTGCCGTGGCGCATCTGAGCGACCACGAATGGTCTCTGGAATCTGATGACCTCAACTTCCAGAAACTTCTGGCTGACAATCGCCGAGGATCTAGCGGCCACAATATCGCCACGTGTCCGATTCGCCACGAGGGCATCCGACGTGGCACCCCCCATTCGAGTGAAATTGATACGCAAATCGTTTTTATGGATCAACAGCGATAAGGGCACCTGCTCAGAGTCAAAGCGGGCCGAGAACTTTCTCTCTCCTGGGAGAAAAAAGCTGAGAAAATCGGAGAAAATTTTGAACTTTGATTTTTTGGTCGCTTAATGGGGGGATGGACCC

>Chr24-77

TCATCTTGTTGACGTGGCGCATCTGAGCGACCACGAATGGTCTCTGGAATCTGATGACCTCAACTTCCAGAAACTTCTGGCTGACAATCGCCGAGGATCTAGCGGCCACAATATCGCCACGTGTCCGATTCGCCACGAGGGCATCCGACGTGGCACCCCCCATTCGAGTGAAATTGATACGCAAATCGTTTTTATGGATCAACAGCGATAAGGGCACCTGCTCAGAGTCAAAGCGGGCCGAGAACTTTCTCTCTCCTGGGAGAAAAAAGCTGAGAAAATCGGAGAAAATTTTGAACTTTGATTTTTTGGTCGCTATAAT

>Chr24-78

GCATGGTTTGACGTGGCGCATCTGAGCGACCACGAATGGTCTCTGGAATCTGATGACCTCAACTTCCAGAAACTTCTGGCTGACAATCGCCGAGGATCTAGCGGCCACAATATCGCCACGTGTCCGATTCGCCACGAGGGCATCCGACGTGGCACCCCCCATTCGAGTGAAATTGATACGCAAATCGTTTTTATGGATCAACAGCGATTAGGGCACCTGCTCAGAGTCAAAGCGGGCCGAGAACTTTCTCTCTCCTGGGAGAAAAAAGCTGAGAAAATCGGAGAAAATTTTGAACTTTGATTTTTTGGTCGCTATAAT

>Chr24-79

TGCATCGGGTTTGACGTGGCGCATCTGAGCGACCACGAATGGTCTCTGGAATCTGATGACCTCAACTTCCAGAAACTTCTGGCTGACAATCGCCGAGGATCTAGCGGCCACAATATCGCCACGTGTCCGATTCGCCACGAGGGTATCCGACGTGGCACCCCCCGTTCGAGTGAAATTGATACGCAAATCGTTTTTATGGATCAACAGCGATTAGGGCACCTGCTCAGAGTCAAAGCGGGCCGAGAACTTTCTCTCTCCTGGGAGAAAAAAGCTGAGAAAATCGGAGAAAATTTTGAACTTTGATTTTTTGGTCGCTATAAT

>Chr24-80

TCATTGGTTTGACGTGGCGCATCTGAGCGACCACGAATGGTCTCTGGAATCTGATGACCTCAACTTCCAGAAACTTCTGGCTGACAATCGCCGAGGATCTAGCGGCCACAATATCGCCACGTGTCCGATTCGCCACGAGGGCATCCGACGTGGCACCCCCCATTCGAGTGAAATTGATACGCAAATCGTTTTTATGGATCAACAGCGATTAGGGCACCTGCTCAGAGTCAAAGCGGGCCGAGAACTTTCTCTCTCCTGGGAGAAAAAAGCTGAGAAAATCGGAGAAAATTTTGAACTTTGATTTTTTGGTCGCTATATTGGGT

>Chr24-81

GCATCGTTGCGTGGCGCATCTGAGCGACCACGAATGGTCTCTGGAATCTGATGACCTCAACTTCCAGAAACTTCTGGCTGACAATCGCCGAGGATCTAGCGGCCACAATATCGCCACGTGTCCGATTCGCCACGAGGGCATCCGACGTGGCACCCCCCATTCGAGTGAAATTGATACGCAAATCGTTTTTATGGATCAACAGCGATTAGGGCACCTGCTCAGAGTCAAAGCGGGCCGAGAACTTTCTCTCTCCTGGGAGAAAAAAGCTGAGAAAATCGGAGAAAATTTTGAACTTTGATTTTTTGGTCGCTATAATGGGGT

>Chr24-82

GCATCGGGATGCCGTGGCGCATCTGAGCGACCACGAATGGTCTCTGGAATCTGATGACCTCAACTTCCAGAAACTTCTGGCTGACAATCGCCGAGGATCTAGCGGCCACAATATCGCCACGTGTCCGATTCGCCACGAGGGCATCCGACGTGGCACCCCCCATTCGAGTGAAATTGATACGCAAATCGTTTTTATGGATCAACAGCGATTAGGGCACCTGCTCAGAGTCAAAGCGGGCCGAGAACTTTCTCTCTCCTGGGAGAAAAAAGCTGAGAAAATCGGAGAAAATTTTGAACTTTGATTTTTTGGTCGCTATAATG

>Chr24-83

CCTTTGATGACGTGGCGCATCTGAGCGACCACGAATGGTCTCTGGAATCTGATGACCTCAACTTCCAGAAACTTCTGGCTGACAATCGCCGAGGATCTAGCGGCCACAATATCGCCACGTGTCCGATTCGCCACGAGGGCATCCGACGTGGCACCCCCCATTCGAGTGAAATTGATACGCAAATCGTTTTTATGGATCAACAGCGATTAGGGCACCTGCTCAGAGTCAAAGCGGGCCGAGAACTTTCTCTCTCCTGGGAGAAAAAAGCTGAGAAAATCGGAGAAAATTTTGAACTTTGATTTTTTGGTCGCTATAATGGGTGATGGACT

>Chr24-84

CGCATCGATCTGCCGTGGCGCATCTGAGCGACCACGAATGGTCTCTGGAATCTGATGACCTCAACTTCCAGAAACTTCTGGCTGACAATCGCCGAGGATCTAGCGGCCACAATATCGCCACGTGTCCGATTCGCCACGAGGGCATCCGACGTGGCACCCCCCATTCGAGTGAAATTGATACGCAAATCGTTTTTATGGATCAACAGCGATTAGGGCACCTGCTCAGAGTCAAAGCGGGCCGAGAACTTTCTCTCTCCTGGGAGAAAAAAGCTGAGAAAATCGGAGAAAATTTTGAACTTTGATTTTTTGGTCGCTATGAATGGTGATGGAG

>Chr24-85

CGCATGGGATGACGTGGCGCATCTGAGCGACCACGAATGGTCTCTGGAATCTGATGACCTCAACTTCCAGAAACTTCTGGCTGACAATCGCCGAGGATCTAGCGGCCACAATATCGCCACGTGTCCGATTCGCCACGAGGGCATCCGACGTGGCACCCCCCATTCGAGTGAAATTGATACGCAAATCGTTTTTATGGATCAACAGCGATTAGGGCACCTGCTCAGAGTCAAAGCGGGCCGAGAACTTTCTCTCTCCTGGGAGAAAAAAGCTGAGAAAATCGGAGAAAATTTTGAACTTTGATTTTTTGGTCGCTATAAT

>Chr24-86

GCATCTGGTTGCGTGGCGCATCTGAGCGACCACGAATGGTCTCTGGAATCTGATGACCTCAACTTCCAGAAACTTCTGGCTGACAATCGCCGAGGATCTAGCGGCCACAATATCGCCACGTGTCCGATTCGCCACGAGGGCATCCGACGTGGCACCCCCCATTCGAGTGAAATTGATACGCAAATCGTTTTTATGGATCAACAGCGATTAGGGCACCTGCTCAGAGTCAAAGCGGGCCGAGAACTTTCTCTCTCCTGGGAGAAAAAAGCTGAGAAAATCGGAGAAAATTTTGAACTTTGATTTTTTGGTCGCTTGATT

>Chr24-87

GCAGCGGTTGCCGTGGCGCATCTGAGCGACCACGAATGGTCTCTGGAATCTGATGACCTCAACTTCCAGAAACTTCTGGCTGACAATCGCCGAGGATCTAGCGGCCACAATATCGCCACGTGTCCGATTCGCCACGAGGGCATCCGACGTGGCACCCCCCATTCGAGTGAAATTGATACGCAAATCGTTTTTATGGATCAACAGCGATTAGGGCACCTGCTCAGAGTCAAAGCGGGCCGAGAACTTTCTCTCTCCTGGGAGAAAAAAGCTGAGAAAATCGGAGAAAATTTTGAACTTTGATTTTTTGGTCGCTTAATT

>Chr24-88

ACATTGTTGCGTGGCGCATCTGAGCGACCACGAATGGTCTCTGGAATCTGATGACCTCAACTTCCAGAAACTTCTGGCTGACAATCGCCGAGGATCTAGCGGCCACAATATCGCCACGTGTCCGATTCGCCACGAGGGCATCCGACGTGGCACCCCCCATTCGAGTGAAATTGATACGCAAATCGTTTTTATGGATCAACAGCGATTAGGGCACCTGCTCAGAGTCAAAGCGGGCCGAGAACTTTCTCTCTCCTGGGAGAAAAAAGCTGAGAAAATCGGAGAAAATTTTGAACTTTGATTTTTTGGTCGCTTAATT

>Chr24-89

ACATCGGTTGCCGTGGCGCATCTGAGCGACCACGAATGGTCTCTGGAATCTGATGACCTCAACTTCCAGAAACTTCTGGCTGACAATCGCCGAGGATCTAGCGGCCACAATATCGCCACGTGTCCGATTCGCCACGAGGGCATCCGACGTGGCACCCCCCATTCGAGTGAAATTGATACGCAAATCGTTTTTATGGATCAACAGCGATTAGGGCACCTGCTCAGAGTCAAAGCGGGCCGAGAACTTTCTCTCTCCTGGGAGAAAAAAGCTGAGAAAATCGGAGAAAATTTTGAACTTTGATTTTTTGGTCGCTATAAT

>Chr24-90

GCTTCTGTTGCCGTGGCGCATCTGAGCGACCACGAATGGTCTCTGGAATCTGATGACCTCAACTTCCAGAAACTTCTGGCTGACAATCGCCGAGGATCTAGCGGCCACAATATCGCCACGTGTCCGATTCGCCACGAGGGCATCCGACGTGGCACCCCCCATTCGAGTGAAATTGATACGCAAATCGTTTTTATGGATCAACAGCGATTAGGGCACCTGCTCAGAGTCAAAGCGGGCCGAGAACTTTCTCTCTCCTGGGAGAAAAAAGCTGAGAAAATCGGAGAAAATTTTGAACTTTGATTTTTTGGTCGCTATAATGGGGTGATGGAC

>Chr24-91

GGATCGGGTTGCGTGGCGCATCTGAGCGACCACGAATGGTCTCTGGAATCTGATGACCTCAACTTCCAGAAACTTCTGGCTGACAATCGCCGAGGATCTAGCGGCCACAATATCGCCACGTGTCCGATTCGCCACGAGGGCATCCGACGTGGCACCCCCCATTCGAGTGAAATTGATACGCAAATCGTTTTTATGGATCAACAGCGATTAGGGCACCTGCTCAGAGTCAAAGCGGGCCGAGAACTTTCTCTCTCCTGGGAGAAAAAAGCTGAGAAAATCGGAGAAAATTTTGAACTTTGATTTTTTGGTCGCTTAA

>Chr24-92

CCTTTGTTTGCGTGGCGCATCTGAGCGACCACGAATGGTCTCTGGAATCTGATGACCTCAACTTCCAGAAACTTCTGGCTGACAATCGCCGAGGATCTAGCGGCCACAATATCGCCACGTGTCCGATTCGCCACGAGGGCATCCGACGTGGCACCCCCCATTCGAGTGAAATTGATACGCAAATCGTTTTTATGGATCAACAGCGATAAGGGCACCTGCTCAGAGTCAAAGCGGGCCGAGAACTTTCTCTCTCCTGGGAGAAAAAAGCTGAGAAAATCGGAGAAAATTTTGAACTTTGATTTTTTGGTCGCTATGAATGG

>Chr24-93

GGGTTGGGGGCTGCCGTGGCGCATCTGAGCGACCACGAATGGTCTCTGGAATCTGATGACCTCAACTTCCAGAAACTTCTGGCTGACAATCGCCGAGGATCTAGCGGCCACAATATCGCCACGTGTCCGATTCGCCACGAGGGCATCCGACGTGGCACCCCCCATTCGAGTGAAATTGATACGCAAATCGTTTTTATGGATCAACAGCGATTAGGGCACCTGCTCAGAGTCAAAGCGGGCCGAGAACTTTCTCTCTCCTGGGAGAAAAAAGCTGAGAAAATCGGAGAAAATTTTGAACTTTGATTTTTTGGTCGCTATAATGGGGTGATGGA

>Chr24-94

GGCATCTCGTTGACGTGGCGCATCTGAGCGACCACGAATGGTCTCTGGAATCTGATGACCTCAACTTCCAGAAACTTCTGGCTGACAATCGCCGAGGATCTAGCGGCCACAATATCGCCACGTGTCCGATTCGCCACGAGGGCATCCGACGTGGCACCCCCCATTCGAGTGAAATTGATACGCAAATCGTTTTTATGGATCAACAGCGATTAGGGCACCTGCTCAGAGTCAAAGCGGGCCGAGAACTTTCTCTCTCCTGGGAGAAAAAAGCTGAGAAAATCGGAGAAAATTTTGAACTTTGATTTTTTGGTCGCTATAAT

>Chr24-95

GCCATTCGTTGCGTGGCGCATCTGAGCGACCACGAATGGTCTCTGGAATCTGATGACCTCAACTTCCAGAAACTTCTGGCTGACAATCGCCGAGGATCTAGCGGCCACAATATCGCCACGTGTCCGATTCGCCACGAGGGCATCCGACGTGGCACCCCCCATTCGAGTGAAATTGATACGCAAATCGTTTTTATGGATCAACAGCGATTAGGGCACCTGCTCAGAGTCAAAGCGGGCCGAGAACTTTCTCTCTCCTGGGAGAAAAAAGCTGAGAAAATCGGAGAAAATTTTGAACTTTGATTTTTTGGTCGCTTGAAT

>Chr24-96

GCATGGGGTTGCCGTGGCGCATCTGAGCGACCACGAATGGTCTCTGGAATCTGATGACCTCAACTTCCAGAAACTTCTGGCTGACAATCGCCGAGGATCTAGCGGCCACAATATCGCCACGTGTCCGATTCGCCACGAGGGCATCCGACGTGGCACCCCCCATTCGAGTGAAATTGATACGCAAATCGTTTTTATGGATCAACAGCGATTAGGGCACCTGCTCAGAGTCAAAGCGGGCCGAGAACTTTCTCTCTCCTGGGAGAAAAAAGCTGAGAAAATCGGAGAAAATTTTGAACTTTGATTTTTTGGTCGCTTGATGGGG

>Chr24-97

GCTTCGGGTTTGCCGTGGCGCATCTGAGCGACCACGAATGGTCTCTGGAATCTGATGACCTCAACTTCCAGAAACTTCTGGCTGACAATCGCCGAGGATCTAGCGGCCACAATATCGCCACGTGTCCGATTCGCCACGAGGGCATCCGACGTGGCACCCCCCATTCGAGTGAAATTGATACGCAAATCGTTTTTATGGATCAACAGCGATTAGGGCACCTGCTCAGAGTCAAAGCGGGCCGAGAACTTTCTCTCTCCTGGGAGAAAAAAGCTGAGAAAATCGGAGAAAATTTTGAACTTTGATTTTTTGGTCGCTATAATGGGT

>Chr24-98

CACTCCTGCCGTGGCGCATCTGAGCGACCACGAATGGTCTCTGGAATCTGATGACCTCAACTTCCAGAAACTTCTGGCTGACAATCGCCGAGGATCTAGCGGCCACAATATCGCCACGTGTCCGATTCGCCACGAGGGTATCCGACGTGGCACCCCCCGTTCGAGTGAAATTGATACGCAAATCGTTTTTATGGATCAACAGCGATTAGGGCACCTGCTCAGAGTCAAAGCGGGCCGAGAACTTTCTCTCTCCTGGGAGAAAAAAGCTGAGAAAATCGGAGAAAATTTTGAACTTTGATTTTTTGGTCGCTTAAT

>Chr24-99

GGCACTGGTTTGACGTGGCGCATCTGAGCGACCACGAATGGTCTCTGGAATCTGATGACCTCAACTTCCAGAAACTTCTGGCTGACAATCGCCGAGGATCTAGCGGCCACAATATCGCCACGTGTCCGATTCGCCACGAGGGTATCCGACGTGGCACCCCCCGTTCGAGTGAAATTGATACGCAAATCGTTTTTATGGATCAACAGCGATTAGGGCACCTGCTCAGAGTCAAAGCGGGCCGAGAACTTTCTCTCTCCTGGGAGAAAAAAGCTGAGAAAATCGGAGAAAATTTTGAACTTTGATTTTTTGGTCGCTTAATGGGGTGATGGAC

>Chr24-100

GACTTGGTTGACGTGGCGCATCTGAGCGACCACGAATGGTCTCTGGAATCTGATGACCTCAACTTCCAGAAACTTCTGGCTGACAATCGCCGAGGATCTAGCGGCCACAATATCGCCACGTGTCCGATTCGCCACGAGGGCATCCGACGTGGCACCCCCCATTCGAGTGAAATTGATACGCAAATCGTTTTTATGGATCAACAGCGATTAGGGCACCTGCTCAGAGTCAAAGCGGGCCGAGAACTTTCTCTCTCCTGGGAGAAAAAAGCTGAGAAAATCGGAGAAAATTTTGAACTTTGATTTTTTGGTCGCTATAATGG

>Chr24-101

CATCCCCTTGCCGTGGCGCATCTGAGCGACCACGAATGGTCTCTGGAATCTGATGACCTCAACTTCCAGAAACTTCTGGCTGACAATCGCCGAGGATCTAGCGGCCACAATATCGCCACGTGTCCGATTCGCCACGAGGGCATCCGACGTGGCACCCCCCATTCGAGTGAAATTGATACGCAAATCGTTTTTATGGATCAACAGCGATTAGGGCACCTGCTCAGAGTCAAAGCGGGCCGAGAACTTTCTCTCTCCTGGGAGAAAAAAGCTGAGAAAATCGGAGAAAATTTTGAACTTTGATTTTTTGGTCGCTTGAGGGGG

>Chr24-102

GGGGCACGGGTTGCCGTGGCGCATCTGAGCGACCACGAATGGTCTCTGGAATCTGATGACCTCAACTTCCAGAAACTTCTGGCTGACAATCGCCGAGGATCTAGCGGCCACAATATCGCCACGTGTCCGATTCGCCACGAGGGCATCCGACGTGGCACCCCCCATTCGAGTGAAATTGATACGCAAATCGTTTTTATGGATCAACAGCGATTAGGGCACCTGCTCAGAGTCAAAGCGGGCCGAGAACTTTCTCTCTCCTGGGAGAAAAAAGCTGAGAAAATCGGAGAAAATTTTGAACTTTGATTTTTTGGTCGCTTAATG

>Chr24-103

CCATTTGTTGCCGTGGCGCATCTGAGCGACCACGAATGGTCTCTGGAATCTGATGACCTCAACTTCCAGAAACTTCTGGCTGACAATCGCCGAGGATCTAGCGGCCACAATATCGCCACGTGTCCGATTCGCCACGAGGGCATCCGACGTGGCACCCCCCATTCGAGTGAAATTGATACGCAAATCGTTTTTATGGATCAACAGCGATTAGGGCACCTGCTCAGAGTCAAAGCGGGCCGAGAACTTTCTCTCTCCTGGGAGAAAAAAGCTGAGAAAATCGGAGAAAATTTTGAACTTTGATTTTTTGGTCGCTATATT

>Chr24-104

CCATGGGTTTGCCGTGGCGCATCTGAGCGACCACGAATGGTCTCTGGAATCTGATGACCTCAACTTCCAGAAACTTCTGGCTGACAATCGCCGAGGATCTAGCGGCCACAATATCGCCACGTGTCCGATTCGCCACGAGGGCATCCGACGTGGCACCCCCCATTCGAGTGAAATTGATACGCAAATCGTTTTTATGGATCAACAGCGATTAGGGCACCTGCTCAGAGTCAAAGCGGGCCGAGAACTTTCTCTCTCCTGGGAGAAAAAAGCTGAGAAAATCGGAGAAAATTTTGAACTTTGATTTTTTGGTCGCTATAATGG

>Chr24-105

TGCATCTGTTTGACGTGGCGCATCTGAGCGACCACGAATGGTCTCTGGAATCTGATGACCTCAACTTCCAGAAACTTCTGGCTGACAATCGCCGAGGATCTAGCGGCCACAATATCGCCACGTGTCCGATTCGCCACGAGGGCATCCGACGTGGCACCCCCCATTCGAGTGAAATTGATACGCAAATCGTTTTTATGGATCAACAGCGATTAGGGCACCTGCTCAGAGTCAAAGCGGGCCGAGAACTTTCTCTCTCCTGGGAGAAAAAAGCTGAGAAAATCGGAGAAAATTTTGAACTTTGATTTTTTGGTCGCTATATTGGGTGATGGACC

>Chr24-106

GGATCCGGTTGCGTGGCGCATCTGAGCGACCACGAATGGTCTCTGGAATCTGATGACCTCAACTTCCAGAAACTTCTGGCTGACAATCGCCGAGGATCTAGCGGCCACAATATCGCCACGTGTCCGATTCGCCACGAGGGCATCCGACGTGGCACCCCCCATTCGAGTGAAATTGATACGCAAATCGTTTTTATGGATCAACAGCGATAAGGGCACCTGCTCAGAGTCAAAGCGGGCCGAGAACTTTCTCTCTCCTGGGAGAAAAAAGCTGAGAAAATCGGAGAAAATTTTGAACTTTGATTTTTTGGTCGCTTGA

>Chr24-107

GCGTCGGGTTGCCGTGGCGCATCTGAGCGACCACGAATGGTCTCTGGAATCTGATGACCTCAACTTCCAGAAACTTCTGGCTGACAATCGCCGAGGATCTAGCGGCCACAATATCGCCACGTGTCCGATTCGCCACGAGGGCATCCGACGTGGCACCCCCCATTCGAGTGAAATTGATACGCAAATCGTTTTTATGGATCAACAGCGATTAGGGCACCTGCTCAGAGTCAAAGCGGGCCGAGAACTTTCTCTCTCCTGGGAGAAAAAAGCTGAGAAAATCGGAGAAAATTTTGAACTTTGATTTTTTGGTCGCTATAATGGGG

>Chr24-108

GGCACTGGTTGCCGTGGCGCATCTGAGCGACCACGAATGGTCTCTGGAATCTGATGACCTCAACTTCCAGAAACTTCTGGCTGACAATCGCCGAGGATCTAGCGGCCACAATATCGCCACGTGTCCGATTCGCCACGAGGGCATCCGACGTGGCACCCCCCATTCGAGTGAAATTGATACGCAAATCGTTTTTATGGATCAACAGCGATTAGGGCACCTGCTCAGAGTCAAAGCGGGCCGAGAACTTTCTCTCTCCTGGGAGAAAAAAGCTGAGAAAATCGGAGAAAATTTTGAACTTTGATTTTTTGGTCGCTATGAT

>Chr24-109

GGCATCTGGTTGACGTGGCGCATCTGAGCGACCACGAATGGTCTCTGGAATCTGATGACCTCAACTTCCAGAAACTTCTGGCTGACAATCGCCGAGGATCTAGCGGCCACAATATCGCCACGTGTCCGATTCGCCACGAGGGCATCCGACGTGGCACCCCCCATTCGAGTGAAATTGATACGCAAATCGTTTTTATGGATCAACAGCGATTAGGGCACCTGCTCAGAGTCAAAGCGGGCCGAGAACTTTCTCTCTCCTGGGAGAAAAAAGCTGAGAAAATCGGAGAAAATTTTGAACTTTGATTTTTTGGTCGCTTAATG

>Chr24-110

GCACTCGTTGACGTGGCGCATCTGAGCGACCACGAATGGTCTCTGGAATCTGATGACCTCAACTTCCAGAAACTTCTGGCTGACAATCGCCGAGGATCTAGCGGCCACAATATCGCCACGTGTCCGATTCGCCACGAGGGCATCCGACGTGGCACCCCCCATTCGAGTGAAATTGATACGCAAATCGTTTTTATGGATCAACAGCGATTAGGGCACCTGCTCAGAGTCAAAGCGGGCCGAGAACTTTCTCTCTCCTGGGAGAAAAAAGCTGAGAAAATCGGAGAAAATTTTGAACTTTGATTTTTTGGTCGCTTAG

>Chr24-111

CATCGCGTTTGCCGTGGCGCATCTGAGCGACCACGAATGGTCTCTGGAATCTGATGACCTCAACTTCCAGAAACTTCTGGCTGACAATCGCCGAGGATCTAGCGGCCACAATATCGCCACGTGTCCGATTCGCCACGAGGGCATCCGACGTGGCACCCCCCATTCGAGTGAAATTGATACGCAAATCGTTTTTATGGATCAACAGCGATTAGGGCACCTGCTCAGAGTCAAAGCGGGCCGAGAACTTTCTCTCTCCTGGGAGAAAAAAGCTGAGAAAATCGGAGAAAATTTTGAACTTTGATTTTTTGGTCGCTTAAGG

>Chr24-112

GGTTCTGGATGCGTGGCGCATCTGAGCGACCACGAATGGTCTCTGGAATCTGATGACCTCAACTTCCAGAAACTTCTGGCTGACAATCGCCGAGGATCTAGCGGCCACAATATCGCCACGTGTCCGATTCGCCACGAGGGCATCCGACGTGGCACCCCCCATTCGAGTGAAATTGATACGCAAATCGTTTTTATGGATCAACAGCGATTAGGGCACCTGCTCAGAGTCAAAGCGGGCCGAGAACTTTCTCTCTCCTGGGAGAAAAAAGCTGAGAAAATCGGAGAAAATTTTGAACTTTGATTTTTTGGTCGCTATAAT

>Chr24-113

GCCTCCCGTTGCCGTGGCGCATCTGAGCGACCACGAATGGTCTCTGGAATCTGATGACCTCAACTTCCAGAAACTTCTGGCTGACAATCGCCGAGGATCTAGCGGCCACAATATCGCCACGTGTCCGATTCGCCACGAGGGCATCCGACGTGGCACCCCCCATTCGAGTGAAATTGATACGCAAATCGTTTTTATGGATCAACAGCGATTAGGGCACCTGCTCAGAGTCAAAGCGGGCCGAGAACTTTCTCTCTCCTGGGAGAAAAAAGCTGAGAAAATCGGAGAAAATTTTGAACTTTGATTTTTTGGTCGCTATAAT

>Chr24-114

CACGGGGTGCCGTGGCGCATCTGAGCGACCACGAATGGTCTCTGGAATCTGATGACCTCAACTTCCAGAAACTTCTGGCTGACAATCGCCGAGGATCTAGCGGCCACAATATCGCCACGTGTCCGATTCGCCACGAGGGTATCCGACGTGGCACCCCCCGTTCGAGTGAAATTGATACGCAAATCGTTTTTATGGATCAACAGCGATTAGGGCACCTGCTCAGAGTCAAAGCGGGCCGAGAACTTTCTCTCTCCTGGGAGAAAAAAGCTGAGAAAATCGGAGAAAATTTTGAACTTTGATTTTTTGGTCGCTTAAT

>Chr24-115

GCGTTGTGGATGCCGTGGCGCATCTGAGCGACCACGAATGGTCTCTGGAATCTGATGACCTCAACTTCCAGAAACTTCTGGCTGACAATCGCCGAGGATCTAGCGGCCACAATATCGCCACGTGTCCGATTCGCCACGAGGGCATCCGACGTGGCACCCCCCATTCGAGTGAAATTGATACGCAAATCGTTTTTATGGATCAACAGCGATTAGGGCACCTGCTCAGAGTCAAAGCGGGCCGAGAACTTTCTCTCTCCTGGGAGAAAAAAGCTGAGAAAATCGGAGAAAATTTTGAACTTTGATTTTTTGGTCGCTATAATTGGGTGATGGA

>Chr24-116

GCACTGGGATGCCGTGGCGCATCTGAGCGACCACGAATGGTCTCTGGAATCTGATGACCTCAACTTCCAGAAACTTCTGGCTGACAATCGCCGAGGATCTAGCGGCCACAATATCGCCACGTGTCCGATTCGCCACGAGGGCATCCGACGTGGCACCCCCCATTCGAGTGAAATTGATACGCAAATCGTTTTTATGGATCAACAGCGATAAGGGCACCTGCTCAGAGTCAAAGCGGGCCGAGAACTTTCTCTCTCCTGGGAGAAAAAAGCTGAGAAAATCGGAGAAAATTTTGAACTTTGATTTTTTGGTCGCTTGATGGGG

>Chr24-117

GCATCTGGGTTGCCGTGGCGCATCTGAGCGACCACGAATGGTCTCTGGAATCTGATGACCTCAACTTCCAGAAACTTCTGGCTGACAATCGCCGAGGATCTAGCGGCCACAATATCGCCACGTGTCCGATTCGCCACGAGGGCATCCGACGTGGCACCCCCCATTCGAGTGAAATTGATACGCAAATCGTTTTTATGGATCAACAGCGATTAGGGCACCTGCTCAGAGTCAAAGCGGGCCGAGAACTTTCTCTCTCCTGGGAGAAAAAAGCTGAGAAAATCGGAGAAAATTTTGAACTTTGATTTTTTGGTCGCTATAAT

>Chr24-118

GCTTGGGTTGCGTGGCGCATCTGAGCGACCACGAATGGTCTCTGGAATCTGATGACCTCAACTTCCAGAAACTTCTGGCTGACAATCGCCGAGGATCTAGCGGCCACAATATCGCCACGTGTCCGATTCGCCACGAGGGCATCCGACGTGGCACCCCCCATTCGAGTGAAATTGATACGCAAATCGTTTTTATGGATCAACAGCGATTAGGGCACCTGCTCAGAGTCAAAGCGGGCCGAGAACTTTCTCTCTCCTGGGAGAAAAAAGCTGAGAAAATCGGAGAAAATTTTGAACTTTGATTTTTTGGTCGCTATAATGGG

>Chr24-119

GCATCTGTTTGACGTGGCGCATCTGAGCGACCACGAATGGTCTCTGGAATCTGATGACCTCAACTTCCAGAAACTTCTGGCTGACAATCGCCGAGGATCTAGCGGCCACAATATCGCCACGTGTCCGATTCGCCACGAGGGCATCCGACGTGGCACCCCCCATTCGAGTGAAATTGATACGCAAATCGTTTTTATGGATCAACAGCGATTAGGGCACCTGCTCAGAGTCAAAGCGGGCCGAGAACTTTCTCTCTCCTGGGAGAAAAAAGCTGAGAAAATCGGAGAAAATTTTGAACTTTGATTTTTTGGTCGCTTAATGGGGTGATGGACCC

>Chr24-120

GGCATTGGTATGACGTGGCGCATCTGAGCGACCACGAATGGTCTCTGGAATCTGATGACCTCAACTTCCAGAAACTTCTGGCTGACAATCGCCGAGGATCTAGCGGCCACAATATCGCCACGTGTCCGATTCGCCACGAGGGCATCCGACGTGGCACCCCCCATTCGAGTGAAATTGATACGCAAATCGTTTTTATGGATCAACAGCGATTAGGGCACCTGCTCAGAGTCAAAGCGGGCCGAGAACTTTCTCTCTCCTGGGAGAAAAAAGCTGAGAAAATCGGAGAAAATTTTGAACTTTGATTTTTTGGTCGCTATAAT

>Chr24-121

GGCACGGGGCTGACGTGGCGCATCTGAGCGACCACGAATGGTCTCTGGAATCTGATGACCTCAACTTCCAGAAACTTCTGGCTGACAATCGCCGAGGATCTAGCGGCCACAATATCGCCACGTGTCCGATTCGCCACGAGGGCATCCGACGTGGCACCCCCCATTCGAGTGAAATTGATACGCAAATCGTTTTTATGGATCAACAGCGATTAGGGCACCTGCTCAGAGTCAAAGCGGGCCGAGAACTTTCTCTCTCCTGGGAGAAAAAAGCTGAGAAAATCGGAGAAAATTTTGAACTTTGATTTTTTGGTCGCTTAATGG

>Chr24-122

CTTCGCCTTGACGTGGCGCATCTGAGCGACCACGAATGGTCTCTGGAATCTGATGACCTCAACTTCCAGAAACTTCTGGCTGACAATCGCCGAGGATCTAGCGGCCACAATATCGCCACGTGTCCGATTCGCCACGAGGGCATCCGACGTGGCACCCCCCATTCGAGTGAAATTGATACGCAAATCGTTTTTATGGATCAACAGCGATTAGGGCACCTGCTCAGAGTCAAAGCGGGCCGAGAACTTTCTCTCTCCTGGGAGAAAAAAGCTGAGAAAATCGGAGAAAATTTTGAACTTTGATTTTTTGGTCGCTTAGAGGG

>Chr24-123

GGCACTGGTTTGACGTGGCGCATCTGAGCGACCACGAATGGTCTCTGGAATCTGATGACCTCAACTTCCAGAAACTTCTGGCTGACAATCGCCGAGGATCTAGCGGCCACAATATCGCCACGTGTCCGATTCGCCACGAGGGTATCCGACGTGGCACCCCCCGTTCGAGTGAAATTGATACGCAAATCGTTTTTATGGATCAACAGCGATTAGGGCACCTGCTCAGAGTCAAAGCGGGCCGAGAACTTTCTCTCTCCTGGGAGAAAAAAGCTGAGAAAATCGGAGAAAATTTTGAACTTTGATTTTTTGGTCGCTTAATGGGGTGATGGAC

>Chr24-124

GCATCTGGGTTGCCGTGGCGCATCTGAGCGACCACGAATGGTCTCTGGAATCTGATGACCTCAACTTCCAGAAACTTCTGGCTGACAATCGCCGAGGATCTAGCGGCCACAATATCGCCACGTGTCCGATTCGCCACGAGGGCATCCGACGTGGCACCCCCCATTCGAGTGAAATTGATACGCAAATCGTTTTTATGGATCAACAGCGATTAGGGCACCTGCTCAGAGTCAAAGCGGGCCGAGAACTTTCTCTCTCCTGGGAGAAAAAAGCTGAGAAAATCGGAGAAAATTTTGAACTTTGATTTTTTGGTCGCTATAAT

>Chr24-125

CGCATCTGGTTGCGTGGCGCATCTGAGCGACCACGAATGGTCTCTGGAATCTGATGACCTCAACTTCCAGAAACTTCTGGCTGACAATCGCCGAGGATCTAGCGGCCACAATATCGCCACGTGTCCGATTCGCCACGAGGGCATCCGACGTGGCACCCCCCATTCGAGTGAAATTGATACGCAAATCGTTTTTATGGATCAACAGCGATTAGGGCACCTGCTCAGAGTCAAAGCGGGCCGAGAACTTTCTCTCTCCTGGGAGAAAAAAGCTGAGAAAATCGGAGAAAATTTTGAACTTTGATTTTTTGGTCGCTTGATGGGGG

>Chr24-126

GGCATTCGGTTGACGTGGCGCATCTGAGCGACCACGAATGGTCTCTGGAATCTGATGACCTCAACTTCCAGAAACTTCTGGCTGACAATCGCCGAGGATCTAGCGGCCACAATATCGCCACGTGTCCGATTCGCCACGAGGGCATCCGACGTGGCACCCCCCATTCGAGTGAAATTGATACGCAAATCGTTTTTATGGATCAACAGCGATTAGGGCACCTGCTCAGAGTCAAAGCGGGCCGAGAACTTTCTCTCTCCTGGGAGAAAAAAGCTGAGAAAATCGGAGAAAATTTTGAACTTTGATTTTTTGGTCGCTTAA

>Chr24-127

GGCTCTCGATGCCGTGGCGCATCTGAGCGACCACGAATGGTCTCTGGAATCTGATGACCTCAACTTCCAGAAACTTCTGGCTGACAATCGCCGAGGATCTAGCGGCCACAATATCGCCACGTGTCCGATTCGCCACGAGGGCATCCGACGTGGCACCCCCCATTCGAGTGAAATTGATACGCAAATCGTTTTTATGGATCAACAGCGATTAGGGCACCTGCTCAGAGTCAAAGCGGGCCGAGAACTTTCTCTCTCCTGGGAGAAAAAAGCTGAGAAAATCGGAGAAAATTTTGAACTTTGATTTTTTGGTCGCTATAAT

>Chr24-128

CCTTGTGTTGACGTGGCGCATCTGAGCGACCACGAATGGTCTCTGGAATCTGATGACCTCAACTTCCAGAAACTTCTGGCTGACAATCGCCGAGGATCTAGCGGCCACAATATCGCCACGTGTCCGATTCGCCACGAGGGCATCCGACGTGGCACCCCCCATTCGAGTGAAATTGATACGCAAATCGTTTTTATGGATCAACAGCGATTAGGGCACCTGCTCAGAGTCAAAGCGGGCCGAGAACTTTCTCTCTCCTGGGAGAAAAAAGCTGAGAAAATCGGAGAAAATTTTGAACTTTGATTTTTTGGTCGCTATATT

>Chr24-129

GCCTTTGGTTGCCGTGGCGCATCTGAGCGACCACGAATGGTCTCTGGAATCTGATGACCTCAACTTCCAGAAACTTCTGGCTGACAATCGCCGAGGATCTAGCGGCCACAATATCGCCACGTGTCCGATTCGCCACGAGGGCATCCGACGTGGCACCCCCCATTCGAGTGAAATTGATACGCAAATCGTTTTTATGGATCAACAGCGATAAGGGCACCTGCTCAGAGTCAAAGCGGGCCGAGAACTTTCTCTCTCCTGGGAGAAAAAAGCTGAGAAAATCGGAGAAAATTTTGAACTTTGATTTTTTGGTCGCTATAAT

>Chr24-130

GCATCGGTTGACGTGGCGCATCTGAGCGACCACGAATGGTCTCTGGAATCTGATGACCTCAACTTCCAGAAACTTCTGGCTGACAATCGCCGAGGATCTAGCGGCCACAATATCGCCACGTGTCCGATTCGCCACGAGGGCATCCGACGTGGCACCCCCCATTCGAGTGAAATTGATACGCAAATCGTTTTTATGGATCAACAGCGATAAGGGCACCTGCTCAGAGTCAAAGCGGGCCGAGAACTTTCTCTCTCCTGGGAGAAAAAAGCTGAGAAAATCGGAGAAAATTTTGAACTTTGATTTTTTGGTCGCTATAATGGGGTGATGGAA

>Chr24-131

CACGGGGTGCCGTGGCGCATCTGAGCGACCACGAATGGTCTCTGGAATCTGATGACCTCAACTTCCAGAAACTTCTGGCTGACAATCGCCGAGGATCTAGCGGCCACAATATCGCCACGTGTCCGATTCGCCACGAGGGTATCCGACGTGGCACCCCCCGTTCGAGTGAAATTGATACGCAAATCGTTTTTATGGATCAACAGCGATTAGGGCACCTGCTCAGAGTCAAAGCGGGCCGAGAACTTTCTCTCTCCTGGGAGAAAAAAGCTGAGAAAATCGGAGAAAATTTTGAACTTTGATTTTTTGGTCGCTTAAT

>Chr24-132

GCATCTGGATGACGTGGCGCATCTGAGCGACCACGAATGGTCTCTGGAATCTGATGACCTCAACTTCCAGAAACTTCTGGCTGACAATCGCCGAGGATCTAGCGGCCACAATATCGCCACGTGTCCGATTCGCCACGAGGGCATCCGACGTGGCACCCCCCATTCGAGTGAAATTGATACGCAAATCGTTTTTATGGATCAACAGCGATTAGGGCACCTGCTCAGAGTCAAAGCGGGCCGAGAACTTTCTCTCTCCTGGGAGAAAAAAGCTGAGAAAATCGGAGAAAATTTTGAACTTTGATTTTTTGGTCGCTATAATG

>Chr24-133

CTTCGCCTTGACGTGGCGCATCTGAGCGACCACGAATGGTCTCTGGAATCTGATGACCTCAACTTCCAGAAACTTCTGGCTGACAATCGCCGAGGATCTAGCGGCCACAATATCGCCACGTGTCCGATTCGCCACGAGGGCATCCGACGTGGCACCCCCCATTCGAGTGAAATTGATACGCAAATCGTTTTTATGGATCAACAGCGATTAGGGCACCTGCTCAGAGTCAAAGCGGGCCGAGAACTTTCTCTCTCCTGGGAGAAAAAAGCTGAGAAAATCGGAGAAAATTTTGAACTTTGATTTTTTGGTCGCTTAGAGGG

>Chr24-134

GGCTCTCGATGCCGTGGCGCATCTGAGCGACCACGAATGGTCTCTGGAATCTGATGACCTCAACTTCCAGAAACTTCTGGCTGACAATCGCCGAGGATCTAGCGGCCACAATATCGCCACGTGTCCGATTCGCCACGAGGGCATCCGACGTGGCACCCCCCATTCGAGTGAAATTGATACGCAAATCGTTTTTATGGATCAACAGCGATTAGGGCACCTGCTCAGAGTCAAAGCGGGCCGAGAACTTTCTCTCTCCTGGGAGAAAAAAGCTGAGAAAATCGGAGAAAATTTTGAACTTTGATTTTTTGGTCGCTATAAT

>Chr24-135

CGCACGGTATGACGTGGCGCATCTGAGCGACCACGAATGGTCTCTGGAATCTGATGACCTCAACTTCCAGAAACTTCTGGCTGACAATCGCCGAGGATCTAGCGGCCACAATATCGCCACGTGTCCGATTCGCCACGAGGGCATCCGACGTGGCACCCCCCATTCGAGTGAAATTGATACGCAAATCGTTTTTATGGATCAACAGCGATTAGGGCACCTGCTCAGAGTCAAAGCGGGCCGAGAACTTTCTCTCTCCTGGGAGAAAAAAGCTGAGAAAATCGGAGAAAATTTTGAACTTTGATTTTTTGGTCGCTATAATGGGTGATGGACC

>Chr24-136

CGCATGCGATGACGTGGCGCATCTGAGCGACCACGAATGGTCTCTGGAATCTGATGACCTCAACTTCCAGAAACTTCTGGCTGACAATCGCCGAGGATCTAGCGGCCACAATATCGCCACGTGTCCGATTCGCCACGAGGGTATCCGACGTGGCACCCCCCGTTCGAGTGAAATTGATACGCAAATCGTTTTTATGGATCAACAGCGATTAGGGCACCTGCTCAGAGTCAAAGCGGGCCGAGAACTTTCTCTCTCCTGGGAGAAAAAAGCTGAGAAAATCGGAGAAAATTTTGAACTTTGATTTTTTGGTCGCTATAATTGGGTGATGGAC

>Chr24-137

TCATTGTATGACGTGGCGCATCTGAGCGACCACGAATGGTCTCTGGAATCTGATGACCTCAACTTCCAGAAACTTCTGGCTGACAATCGCCGAGGATCTAGCGGCCACAATATCGCCACGTGTCCGATTCGCCACGAGGGCATCCGACGTGGCACCCCCCATTCGAGTGAAATTGATACGCAAATCGTTTTTATGGATCAACAGCGATTAGGGCACCTGCTCAGAGTCAAAGCGGGCCGAGAACTTTCTCTCTCCTGGGAGAAAAAAGCTGAGAAAATCGGAGAAAATTTTGAACTTTGATTTTTTGGTCGCTATAAT

>Chr24-138

TCTTCTGGCTGACGTGGCGCATCTGAGCGACCACGAATGGTCTCTGGAATCTGATGACCTCAACTTCCAGAAACTTCTGGCTGACAATCGCCGAGGATCTAGCGGCCACAATATCGCCACGTGTCCGATTCGCCACGAGGGCATCCGACGTGGCACCCCCCATTCGAGTGAAATTGATACGCAAATCGTTTTTATGGATCAACAGCGATTAGGGCACCTGCTCAGAGTCAAAGCGGGCCGAGAACTTTCTCTCTCCTGGGAGAAAAAAGCTGAGAAAATCGGAGAAAATTTTGAACTTTGATTTTTTGGTCGCTTAATGGGGTGATGGACC

>Chr24-139

GCACGGTTGACGTGGCGCATCTGAGCGACCACGAATGGTCTCTGGAATCTGATGACCTCAACTTCCAGAAACTTCTGGCTGACAATCGCCGAGGATCTAGCGGCCACAATATCGCCACGTGTCCGATTCGCCACGAGGGCATCCGACGTGGCACCCCCCATTCGAGTGAAATTGATACGCAAATCGTTTTTATGGATCAACAGCGATTAGGGCACCTGCTCAGAGTCAAAGCGGGCCGAGAACTTTCTCTCTCCTGGGAGAAAAAAGCTGAGAAAATCGGAGAAAATTTTGAACTTTGATTTTTTGGTCGCTATAATGGGGGGGATGGA

>Chr24-140

CTCTTGGTTGCGTGGCGCATCTGAGCGACCACGAATGGTCTCTGGAATCTGATGACCTCAACTTCCAGAAACTTCTGGCTGACAATCGCCGAGGATCTAGCGGCCACAATATCGCCACGTGTCCGATTCGCCACGAGGGCATCCGACGTGGCACCCCCCATTCGAGTGAAATTGATACGCAAATCGTTTTTATGGATCAACAGCGATTAGGGCACCTGCTCAGAGTCAAAGCGGGCCGAGAACTTTCTCTCTCCTGGGAGAAAAAAGCTGAGAAAATCGGAGAAAATTTTGAACTTTGATTTTTTGGTCGCTATAATGGGTGATGGACC

>Chr24-141

ACTTTGTCTGCCGTGGCGCATCTGAGCGACCACGAATGGTCTCTGGAATCTGATGACCTCAACTTCCAGAAACTTCTGGCTGACAATCGCCGAGGATCTAGCGGCCACAATATCGCCACGTGTCCGATTCGCCACGAGGGCATCCGACGTGGCACCCCCCATTCGAGTGAAATTGATACGCAAATCGTTTTTATGGATCAACAGCGATTAGGGCACCTGCTCAGAGTCAAAGCGGGCCGAGAACTTTCTCTCTCCTGGGAGAAAAAAGCTGAGAAAATCGGAGAAAATTTTGAACTTTGATTTTTTGGTCGCTATAAT

>Chr24-142

GGCAGGGGCTGACGTGGCGCATCTGAGCGACCACGAATGGTCTCTGGAATCTGATGACCTCAACTTCCAGAAACTTCTGGCTGACAATCGCCGAGGATCTAGCGGCCACAATATCGCCACGTGTCCGATTCGCCACGAGGGTATCCGACGTGGCACCCCCCGTTCGAGTGAAATTGATACGCAAATCGTTTTTATGGATCAACAGCGATTAGGGCACCTGCTCAGAGTCAAAGCGGGCCGAGAACTTTCTCTCTCCTGGGAGAAAAAAGCTGAGAAAATCGGAGAAAATTTTGAACTTTGATTTTTTGGTCGCTATAATGGG

>Chr24-143

CGCATGGGGAGCCGTGGCGCATCTGAGCGACCACGAATGGTCTCTGGAATCTGATGACCTCAACTTCCAGAAACTTCTGGCTGACAATCGCCGAGGATCTAGCGGCCACAATATCGCCACGTGTCCGATTCGCCACGAGGGCATCCGACGTGGCACCCCCCATTCGAGTGAAATTGATACGCAAATCGTTTTTATGGATCAACAGCGATTAGGGCACCTGCTCAGAGTCAAAGCGGGCCGAGAACTTTCTCTCTCCTGGGAGAAAAAAGCTGAGAAAATCGGAGAAAATTTTGAACTTTGATTTTTTGGTCGCTATAAT
